# Atrial Fibroblasts‐Derived Extracellular Vesicles Exacerbate Atrial Arrhythmogenesis

**DOI:** 10.1002/advs.202507627

**Published:** 2025-07-03

**Authors:** Yue Yuan, Xinbo Zhao, Xuejie Han, Yukai Cao, Xuexin Jin, Ling Shi, Xin Bi, Desheng Li, Yun Zhang, Wenbo Ma, Jiahui Song, Zhenwei Pan, Zhiren Zhang, Yue Li

**Affiliations:** ^1^ Department of Cardiology The First Affiliated Hospital Harbin Medical University Harbin 150001 China; ^2^ Department of Pharmacology Harbin Medical University Harbin 150086 China; ^3^ Department of Pharmacology Key Laboratory of Cardiovascular Research Ministry of Education College of Pharmacy Harbin Medical University Harbin 150086 China; ^4^ NHC Key Laboratory of Cell Transplantation The First Affiliated Hospital Harbin Medical University Harbin 150001 China; ^5^ Key Laboratories of Education Ministry for Myocardial Ischemia Mechanism and Treatment The First Affiliated Hospital Harbin Medical University Harbin 150001 China; ^6^ Key Laboratory of Cardiac Diseases and Heart Failure Harbin Medical University Harbin 150001 China; ^7^ Heilongjiang Key Laboratory for Metabolic Disorder & Cancer Related Cardiovascular Diseases Harbin 150081 China

**Keywords:** atrial fibrillation, atrial fibroblasts, CACNA1C, exosomes, microRNA‐224‐5p

## Abstract

Cardiac fibroblasts (CFs) secrete exosomes, and their cargo represents a new means of cellular communication in cardiovascular diseases, including atrial fibrillation (AF). We aimed to explore the contribution of atial CFs (ACFs)‐derived exosomes to AF development. Cultured primary human ACFs (hACFs) and rat ACFs are treated with angiotensin II, and the secreted exosomes are transferred to rats. Action potential duration and L‐type calcium current (*I*_Ca_) are tested. Global microRNA‐224‐5p knock‐in and fibroblast‐specific microRNA‐224‐5p knock‐in (*FMKI*) mice underwent an inducible AF test. Transferred exosomes of Ang II‐induced hACFs and primary adult rat ACFs increased AF incidence and prolonged AF duration. The inhibitor of exosomes and knockdown of Dicer rescued the AF phenotype. MicroRNA array suggested upregulated microRNA‐224‐5p level in both primary adult rat ACFs and ACFs‐secreted exosomes. microRNA‐224‐5p agonist shortened atrial effective refractory period (AERP) and promoted AF. Mechanistically, microRNA‐224‐5p bound to CACNA1C and inhibited its transcription. Moreover, global microRNA‐224‐5p knock‐in and *FMKI* mice exhibited increased inducible AF incidence, accompanied by diminished *I*_Ca_ current in ACMs. Exosome microRNA‐224‐5p is enhanced in ACFs isolated from atria and plasma of AF patients, and positively correlated with recurrence after radiofrequency ablation. In summary, ACFs‐derived exosome microRNA‐224‐5p contributes to AF by inhibiting CACNA1C to drive atrial electrical remodeling.

## Graphical abstract

Exosome miR‐224‐5p derived from angiotensin II‐treated atrial fibroblasts creates a substrate for AF by promoting atrial electrical remodeling. Increased exosome miR‐224‐5p enhances AF susceptibility by inhibiting CACNA1c expression and decreasing *I*_Ca_ current of atrial cardiomyocytes. The main findings provide new pathophysiological insights that atrial fibroblasts‐secreted exosomes lead to shortened action potential duration of atrial cardiomyocytes and cause AF development.

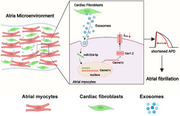

## 1 Introduction

Atrial fibrillation (AF) is a global epidemic arrhythmia in adults with substantial morbidity, mortality, and societal healthcare burden.^[^ ^1^, ^2^ ^]^ Current clinical strategies for AF mainly focus on acute restoration and long‐term maintenance of sinus rhythm by electrical cardioversion, radiofrequency ablation, and/or anti‐arrhythmic drugs.^[^ ^3^, ^4^ ^]^ However, it appears the overall therapeutic effect of these interventions on AF is far from satisfactory and that the AF exhibits a high recurrence rate, ranging from 50% to 63% within 4 weeks.^[^ ^5^ ^]^ Therefore, deciphering the potential mechanisms of AF is urgently needed for developing novel therapeutic interventions.

The pathogenesis of AF is closely related to the molecular and functional disturbances of atrial cardiomyocytes (ACMs) and atrial cardiac fibroblasts (ACFs), the two main cell types in the atrial microenvironment.^[^ ^6^ ^]^ The crosstalk between ACFs and ACMs by paracrine signaling, the most common route of communication, exhibits important effects on AF development. On one hand, ACMs can produce the hormone mediators, which lead to proliferation and further secretion of extracellular matrix (ECM) proteins, triggering fibrosis via stimulating neighboring collagen‐producing ACFs.^[^ ^7^, ^8^ ^]^ On the other hand, ACFs have a high capacity to activate inflammation, hypertrophy, or cell death of ACMs via paracrine interaction as a response to stress stimuli.^[^ ^9^, ^10^ ^]^ However, few studies elucidate the effect of cellular communication between atrial fibroblasts and cardiomyocytes in modulating the electrical physiology of AF arrhythmogenesis.

Exosomes are small endogenous extracellular vesicles (EVs) containing diverse bioactive substances, including miRNAs, mRNAs, DNAs, and proteins etc.^[^ ^11^ ^]^ They are secreted by a variety of cells and are the critical factors contributing to the pathophysiological processes of diverse diseases by mediating intercellular communication.^[^ ^12^ ^]^ Tumor exosome RNAs initiate neutrophil recruitment and lung metastatic niche formation via interacting with Toll‐like receptor 3 in lung epithelial cells.^[^ ^13^ ^]^ The inducible nitric oxide synthase, in the brown adipocytes that secreted exosomes, can be inhibited by the activation of β3‐adrenergic receptors, thereby reducing angiotensin II (Ang II) infusion‐induced cardiac hypertrophy and fibrosis.^[^ ^14^ ^]^ Our previous work also showed that epididymal white adipose tissue (eWAT) promoted Ang II‐treated cardiac fibrosis and subsequent heart dysfunction via secreting miR‐23a‐3p‐enriched exosomes.^[^ ^15^ ^]^

CF is a critical source of exosomes in the heart microenvironment. CFs‐derived exosomes represent a new means of cellular crosstalk, which executes biological functions through trafficking to local or distant cells/organs.^[^ ^16^ ^]^ Indeed, CFs‐derived exosomes are proven to cause cardiac hypertrophy, angiogenesis, and myocardial infarction (MI), and can be the potential biomarkers to predict disease progression.^[^ ^17^ ^]^ Recently, CFs carrying exosomes (Exo‐CFs) have been reported to act as a paracrine mediator to address cardiomyocyte hypertrophy by silencing sorbin and SH3 domain containing (SORBS2) and PDZ and LIM domain 5 (PDLIM5).^[^ ^18^ ^]^ To date, whether and how a specific component of ACFs‐released exosomes plays a critical role in AF pathogenesis remains unexplored.

In this study, we sought to determine whether exosomes from angiotensin II‐induced atrial fibroblasts were involved in the occurrence and maintenance of AF and the potential mechanisms. We found that ACFs‐derived exosome miR‐224‐5p directly bound CACNA1C, a gene encoding Cav1.2 protein and attenuated L‐type calcium current, thereby exacerbating susceptibility to AF via shortening atrial effective refractory period (AERP) in vivo. Besides, the upregulated plasma exosomes microRNA‐224‐5p in AF patients were positively associated with recurrence after radiofrequency ablation. Our study highlights the new mechanism of ACFs secreted EVs triggering atrial arrhythmia and provides a promising therapeutic target for AF.

## 2 Results

### 2.1 Exosomes Derived from Ang II‐Induced Atrial Fibroblasts Increased AF Susceptibility In Vivo

**Figure 1:**
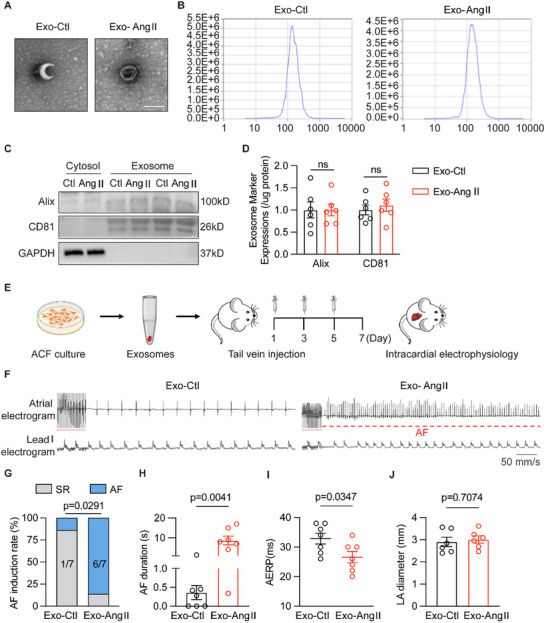
Exosomes derived from Ang II‐treated fibroblasts promoted AF development. A) Representative transmission electron microscopy images of exosomes derived from hACFs (*n* = 3 per group). Scale bar: 100 nm. B) Particle size distribution detected by NanoSight tracking analysis (*n* = 2 per group). C and D) Western blot analysis of the exosome markers Alix (p>0.9999) and CD81 (*p* = 0.849) in cytosol lysis and exosomes of hACFs, GAPDH as control (*n* = 6 per group). E) Scheme for hACFs exosome preparation, injection, and AF induction test in rats. F) Representative simultaneous recordings of surface ECG (lead I) and intracardiac electrograms in Exo‐Ctl and Exo‐Ang II rats after programmed intracardiac stimulation (red line). G) Incidence of pacing inducible AF in two groups of rats (*n* = 7 per group, *p* = 0.0291). H) AF duration in Exo‐Ctl and Exo‐Ang II rats (*n* = 7 per group, *p* = 0.0041). I) AERP in Exo‐Ctl and Exo‐Ang II rats (*n* = 7 per group, *p* = 0.0347). J) LA area in Exo‐Ctl and Exo‐Ang II rats (*n* = 6 per group, *p* = 0.7074). Exo‐Ctl, control hACFs‐derived exosomes; Exo‐Ang II, angiotensin II‐treated hACFs‐derived exosomes; Exo, exosomes; Cyto, cytosol lysis; ECG, electrocardiography; SR, sinus rhythm; AF, atrial fibrillation. The bar graph data are mean±SEM with individual values. *p*‐values were determined with Fisher's exact test in G, and the Mann‐Whitney test in H. *p*‐values were determined with two‐tailed unpaired Student's *t*‐test in D, I, and J.

To clarify the role of ACFs‐secreted exosomes in atrial fibrillation, we first investigated the effects of exosomes from cultured primary human atrial fibroblasts (hACFs) on AF susceptibility in rats. Cultured hACFs were subjected to Ang II treatment, and the secreted exosomes from the cultured medium were extracted. The exosomes were extracted and purified according to the “guidelines of EV”.^[^ ^19^ ^]^ The morphology and the diameter of hACFs‐secreted exosomes were identified by transmission electron microscope (TEM) and Nanosight tracking analysis (**Figure** **1**A,B). Exosome surface markers, including Alix and CD81, were expressed in exosomes, but not in cyto‐lysates (Figure 1C,D). We conducted two different doses of exosomes (low dose, 1×10^8 exosomes/time, and high dose (5×10^8 to 1×10^9) exosomes/time) with tail‐vein injection to test which one was efficient in inducing AF phenotype (Figure S1A, Supporting Information). Both of the injected exosomes were taken up by atrial cardiomyocytes with PKH26 label staining (Figure S1B, Supporting Information). The higher dose of exosomes created a significant AF phenotype compared to the low dose and control group (Figure S1C, Supporting Information). Thus, we performed this dose of exosome injection (5×10^8 to 1×10^9) in the further study (Figure 1E). Then, cardiac echography was performed to evaluate the structure and systolic function of the left ventricle in rats. The left ventricular ejection fraction (LVEF), systolic and diastolic ventricular diameters were comparable in the two groups of animals (Figure S1D–F, Supporting Information). The programmed atrial burst‐pacing test showed that the incidence of reproducible pacing‐induced AF was significantly higher in rats treated with exosomes from Ang II‐treated hACFs (Exo‐Ang II, 85.7%, *p* = 0.0291) than those from control hACFs (Exo‐Ctl, 14.3%) (Figure 1F,G). The duration of AF was longer in Exo‐Ang II than Exo‐Ctl group (Figure 1H), while Exo‐Ang II rats performed shortened AERP (Figure 1I). Besides, the diameter of the left atria (LA) and mitral valve flow velocity had no changes between Exo‐Ang II and Exo‐Ctl rats (Figure 1J; Figure S1G, Supporting Information). Histology did not initiate any inflammation or cell death of atrial cardiomyocytes with comparable fibrosis level in the rat atria (Figure S1H,I, Supporting Information). The above data demonstrated that increased AF susceptibility by exposure to Ang II‐induced hACFs secreted exosomes independent of LV dysfunction and structural remodeling of atria.

To explore the effect of other potential derived components of Ang II‐induced ACFs in promoting AF, we treated control rats with isolated primary adult rat ACFs‐derived exosomes and their left supernatant, then performed a programmed electrical stimulation (PES) experiment. The results showed that only Exo‐Ang II rats suffered from enhanced AF incidence and prolonged AF duration along with attenuated AERP compared to Exo‐Ctl, Supernatant‐Ctl, and Supernatant‐Ang II rats (Figure S2A‐D, Supporting Information). Moreover, we administered control rats with exosome inhibitor, GW4869, to prevent the internal generation of exosomes before transferring ACFs‐derived exosomes. We found comparable AF incidence and AF duration between transferred Exo‐Ang II and GW4869+Exo‐Ang II rat (Figure S2A–C, Supporting Information), which suggested that exogenous Ang II‐treated ACFs secreted exosomes were sufficient to exacerbate AF susceptibility.

### 2.2 miR‐224‐5p Upregulates in the Exosomes from Ang II‐Induced ACFs

**Figure 2:**
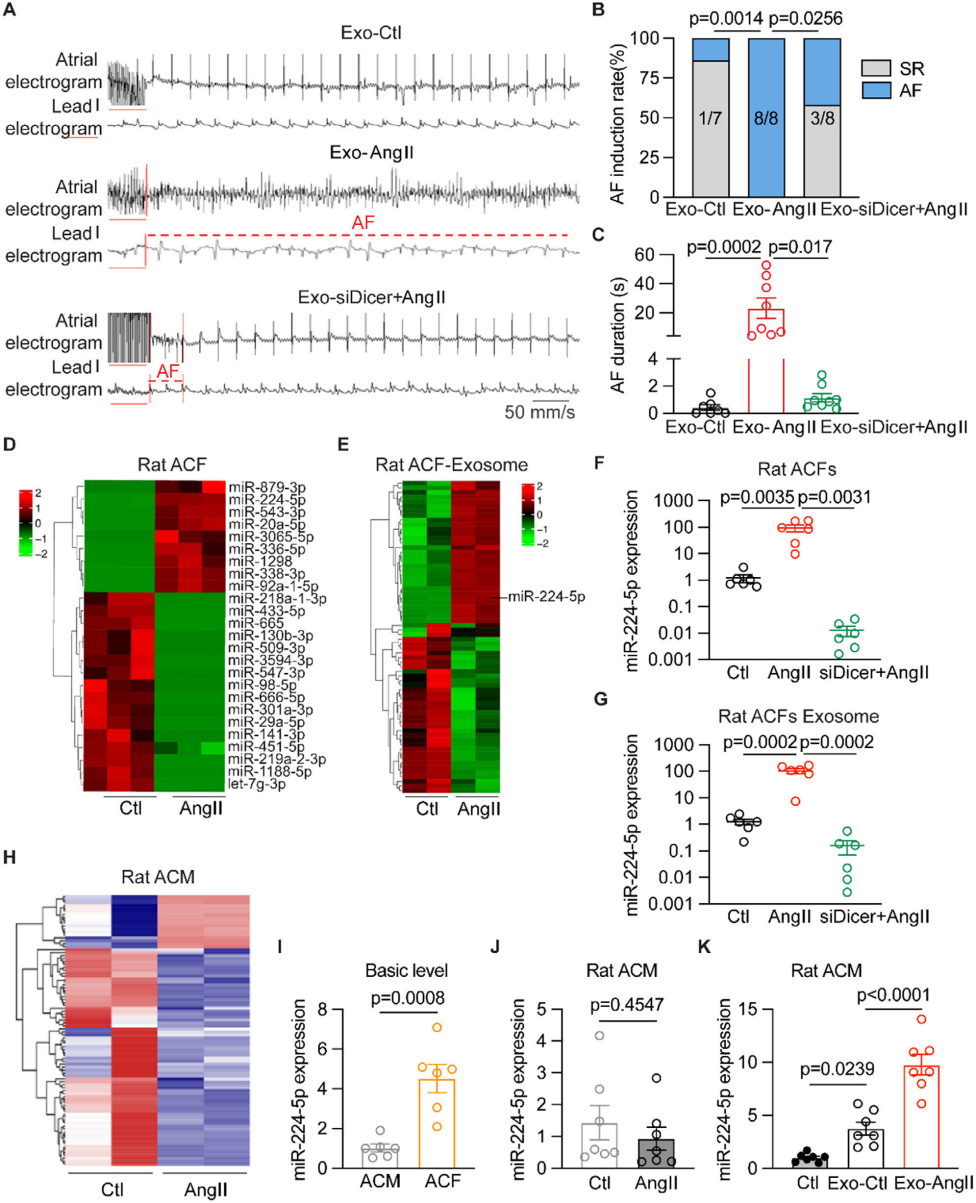
Exosome miR‐224‐5p derived from atrial fibroblasts is a potential molecule enhancing AF inducibility. A) Representative simultaneous recordings of surface ECG (lead I) and intracardiac electrograms in Exo‐Ctl, Exo‐Ang II, and Exo‐siDicer+Ang II rats after programmed intracardiac stimulation (red line). B) Incidence of pacing inducible AF in three groups of rats (*n* = 7 or 8 per group, Exo‐Ctl versus Exo‐Ang II *p* = 0.0014, Exo‐Ang II vs Exo‐siDicer+Ang II *p* = 0.0256). C) AF duration in Exo‐Ctl, Exo‐Ang II, and Exo‐siDicer+Ang II rats (*n* = 7 or 8 per group, Exo‐Ctl vs Exo‐Ang II *p* = 0.0002, Exo‐Ang II vs Exo‐siDicer+Ang II *p* = 0.017). D) Heatmap of differential miRNAs between control and Ang II‐treated cultured primary adult rat ACFs (*n* = 3 per group). E) Heatmap of differential exosome miRNAs secreted from control and Ang II‐treated cultured primary adult rat ACFs (*n* = 2 per group). F and G) miR‐224‐5p expression in both primary adult rat ACFs (*n* = 6 per group, Exo‐Ctl versus Exo‐Ang II *p* = 0.0035, Exo‐Ang II vs Exo‐siDicer+AngII *p* = 0.0031) and ACFs‐derived exosomes by using qRT‐PCR test (*n* = 6 per group, Exo‐Ctl vs Exo‐Ang II *p* = 0.0002, Exo‐Ang II vs Exo‐siDicer+Ang II *p* = 0.0002). H) Heatmap of differential miRNAs between control and Ang II‐treated primary neonatal rat atrial myocytes (*n* = 2 per group). I) miR‐224‐5p in normal primary neonatal rat atrial myocytes and fibroblasts by using qRT‐PCR (*n* = 6 per group, *p* = 0.0008). J) miR‐224‐5p in control and Ang II‐treated primary neonatal rat atrial myocytes by using qRT‐PCR (*n* = 7 per group, *p* = 0.4547). K) miR‐224‐5p level in primary neonatal rat atrial myocytes co‐cultured with ACFs‐derived exosomes by using qRT‐PCR (*n* = 7 per group, Ctl vs Exo‐Ctl *p* = 0.0239, Exo‐Ctl vs Exo‐Ang II p<0.0001). Exo‐Ctl, control cultured primary rat ACFs‐derived exosomes; Exo‐Ang II, angiotensin II‐treated cultured primary rat ACFs‐derived exosomes; Exo‐Ang II+siDicer, knockdown Dicer and angiotensin II‐treated cultured primary rat ACFs‐derived exosomes. The bar graph data are mean±SEM with individual values. *p*‐values were determined with two‐tailed Fisher's exact test in B, and Mann‐Whitney test in C. *p*‐values were determined with one‐way ANOVA and Turkey's multiple comparisons test in F, G, and K. *p*‐values were determined with two‐tailed unpaired Student's *t*‐test in I and J.

MicroRNA (miRNA) is one of the major molecular cargos of EVs to affect the biological communication in different cell types. To address whether miRNA of Ang II‐induced ACFs secreted exosomes exacerbate the AF vulnerability, we knocked down Dicer in Ang II‐subjected primary adult rat ACFs to inhibit its miRNAs generation and transferred the exosomes to control rats. The PES results showed an attenuated inducible AF incidence and duration in Exo‐siDicer+Ang II rats compared to Exo‐Ang II rats (37.5% vs 100%, *p* = 0.0256, **Figure** **2**A–C). No structure change in the atrium was observed in three groups of rats by HE staining (Figure S3A, Supporting Information). These data demonstrated that exosomes secreted by Ang II‐treated ACFs can promote AF development, which is likely to be mediated by miRNAs.

To investigate which exosome miRNA derived from Ang II‐induced ACFs exacerbates AF inducibility, we conducted miRNA sequencing of primary adult rat ACFs and their exosomes in the control and Ang II groups. A total of 9 miRNAs were upregulated, and 16 were downregulated in ACFs after Ang II treatment. Meanwhile, 31 upregulated miRNAs and 37 downregulated miRNAs were found in the exosomes of ACFs (ACFs‐Exo) (Figure 2D,E). There were three differentially expressed miRNAs shown in both ACFs and ACFs‐Exo, including miR‐224‐5p, miR‐92a‐1‐5p, and miR‐29a‐5p (Figure S3C, Supporting Information). miR‐224‐5p was verified to be one of the most differentially expressed miRNAs in ACFs‐Exo after Ang II treatment by qRT‐PCR (Figure 2F,G; Figure S3D,E, Supporting Information). Furthermore, GO analysis indicated that miR‐224‐5p is involved in regulating the biological function of ion channels (Figure S3F, Supporting Information). However, the level of miR‐224‐3p was unaltered in both rat ACFs and their derived exosomes under Ang II stress (Figure S3G,H, Supporting Information). Interestingly, the level of miR‐224‐5p was comparable in both control and Ang II‐treated atrial cardiomyocytes (ACMs) (Figure 2H,J), but the basic level of miR‐224‐5p in ACFs was much higher than ACMs (Figure 2I). The level of miR‐224‐5p increased by nearly five‐fold in ACMs when co‐cultured with control ACFs‐Exo, and 10‐fold with Ang II‐induced ACFs‐Exo (Figure 2K). These data suggested that miR‐224‐5p was enriched in the exosomes of ACFs, which was increased by Ang II and led to a striking change in ACMs.

### 2.3 ACFs‐Derived Exosome miR‐224‐5p Exacerbates AF Susceptibility in Rat

**Figure 3:**
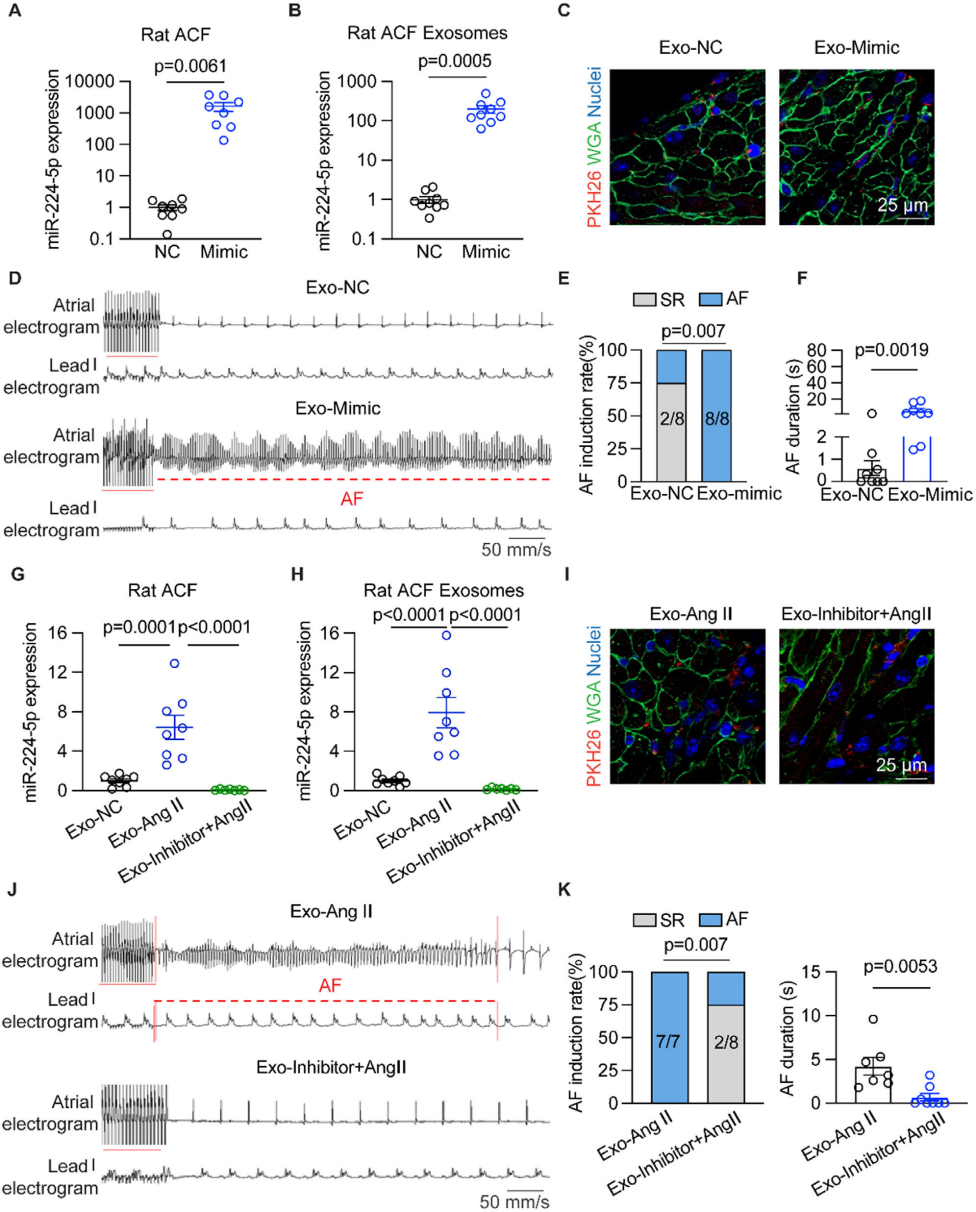
Rat ACFs‐derived exosome miR‐224‐5p exacerbates AF susceptibility in vivo. A) Validation of cellular (*n* = 8 per group, *p* = 0.0061) and B) exosome miR‐224‐5p in cultured primary adult rat ACFs transfected with negative control or miR‐224‐5p mimics (*n* = 9 per group, *p* = 0.0005). C) WGA and PKH‐26 staining in atrial tissues after exosomes were transferred in rats (*n* = 3 per group). Scale bar: 25 µm. D) Representative traces of simultaneous recordings of surface ECG (lead I) and intracardiac electrograms in Exo‐NC and Exo‐Mimic injected rats after programmed intracardiac stimulation (red line). E and F) Incidence rate (*n* = 8 per group, *p* = 0.007) and duration of AF in Exo‐NC and Exo‐Mimic rats (*n* = 8 per group, *p* = 0.0019). G and H) Validation of cellular (*n* = 7 or 8 per group, Exo‐NC vs Exo‐Ang II p = 0.0001, Exo‐Ang II vs Exo‐Inhibitor+Ang II *p* < 0.0001) and exosome miR‐224‐5p expressions in cultured primary adult rat ACFs transfected with negative control or miR‐224‐5p inhibitor (*n* = 7 or 8 per group, Exo‐NC vs Exo‐Ang II *p* < 0.0001, Exo‐Ang II vs Exo‐Inhibitor+Ang II *p* < 0.0001). I) WGA and PKH‐26 label staining in atrial tissues after exosomes were transferred in rats (*n* = 3 per group). Scale bar: 25 µm. J) Representative traces of simultaneous recordings of surface ECG (lead I) and intracardiac electrograms in Exo‐Ang II and Exo‐Inhibitor +Ang II transplant rats after programmed intracardiac stimulation (red line). (K and L) Incidence rate (*n* = 7 or 8 per group, *p* = 0.007) and duration of AF in Exo‐Ang II and Exo‐Inhibitor +Ang II rats (*n* = 7 or 8 per group, *p* = 0.0053). ECG, electrocardiography; SR, sinus rhythm; AF, atrial fibrillation. The bar graph data are mean±SEM with individual values. *p*‐values were determined with Fisher's exact test in E and K, and the Mann‐Whitney test in F and L. *p*‐values were determined with a two‐tailed unpaired Student's *t*‐test in A and B. *p*‐values were determined with one‐way ANOVA and Turkey's multiple comparisons test in G and H.

To determine whether ACF‐restricted exosome miR‐224‐5p promotes atrial arrhythmia, the exosomes collected from negative control and miR‐224‐5p mimics‐transfected primary adult rat ACFs were administered to rats intravenously. Incubation of ACFs with miR‐224‐5p mimics significantly increased miR‐224‐5p levels in both cultured ACFs and derived exosomes (**Figure** **3**A,B). However, miR‐224‐5p showed negative effects on the proliferation and fibrogenesis of ACFs themselves (Figure S4A,B, Supporting Information). PKH‐26 and WGA staining confirmed the entry of ACFs‐derived exosomes into rat atria (Figure 3C). The incidence of pacing‐inducible AF was significantly higher in rats that received exosomes from miR‐224‐5p mimics‐treated ACFs (Exo‐Mimic, 100%, *n* = 8, *p* = 0.007) than those from negative control‐treated ACFs (Exo‐NC, 25%, *n* = 8) (Figure 3D,E). The AF duration was longer (Figure 3F) with shortened AERP (Figure S4D, Supporting Information) in Exo‐Mimic than Exo‐NC rats. The levels of miR‐224‐5p in both plasma exosomes and atria were increased in rats that received exosomes from miR‐224‐5p mimics‐treated ACFs (Figure S4C, Supporting Information). The LA diameter and blood flow of the mitral valve were comparable between the two groups of rats (Figure S4E,F, Supporting Information). HE staining suggested no markable recruited inflammatory cells and fraction of skeletal muscles in the atria of Exo‐Mimic and Exo‐NC rats (Figure S4G, Supporting Information).

Then, we investigated whether inhibition of exosome miR‐224‐5p in ACFs can alleviate AF susceptibility. miR‐224‐5p inhibitor was transfected into ACFs and then subjected to Ang II, and its exosomes were concentrated to be administered into rats. The inhibitor of miR‐224‐5p dramatically reduced its levels in both cultured ACFs and exosomes extracted from the medium (Figure 3G,H). PKH‐26 and WGA staining verified that ACFs‐derived exosomes entered atrial tissues (Figure 3I). Co‐incubation with the miR‐224‐5p inhibitor of ACFs significantly reduced AF occurrence and shortened AF duration (Figure 3J,K) with restored AERP (Figure S4I, Supporting Information). Besides, the levels of miR‐224‐5p in plasma exosomes and atrial tissues were both decreased in rats receiving exosomes from Ang II‐subjected ACFs co‐incubated with miR‐224‐5p inhibitors (Figure S4H, Supporting Information). There were no differences in LA structure and function change between the two groups of rats based on LA diameter, E and A velocity, and HE staining (Figure S4J–L, Supporting Information). The above results implied that miR‐224‐5p was one of the critical molecules of exosomes derived from Ang II‐treated ACFs to promote AF independent of inducing atrial structural remodeling.

### 2.4 miR‐224‐5p Promotes AF Pathogenesis and Abbreviates APD of ACMs

**Figure 4:**
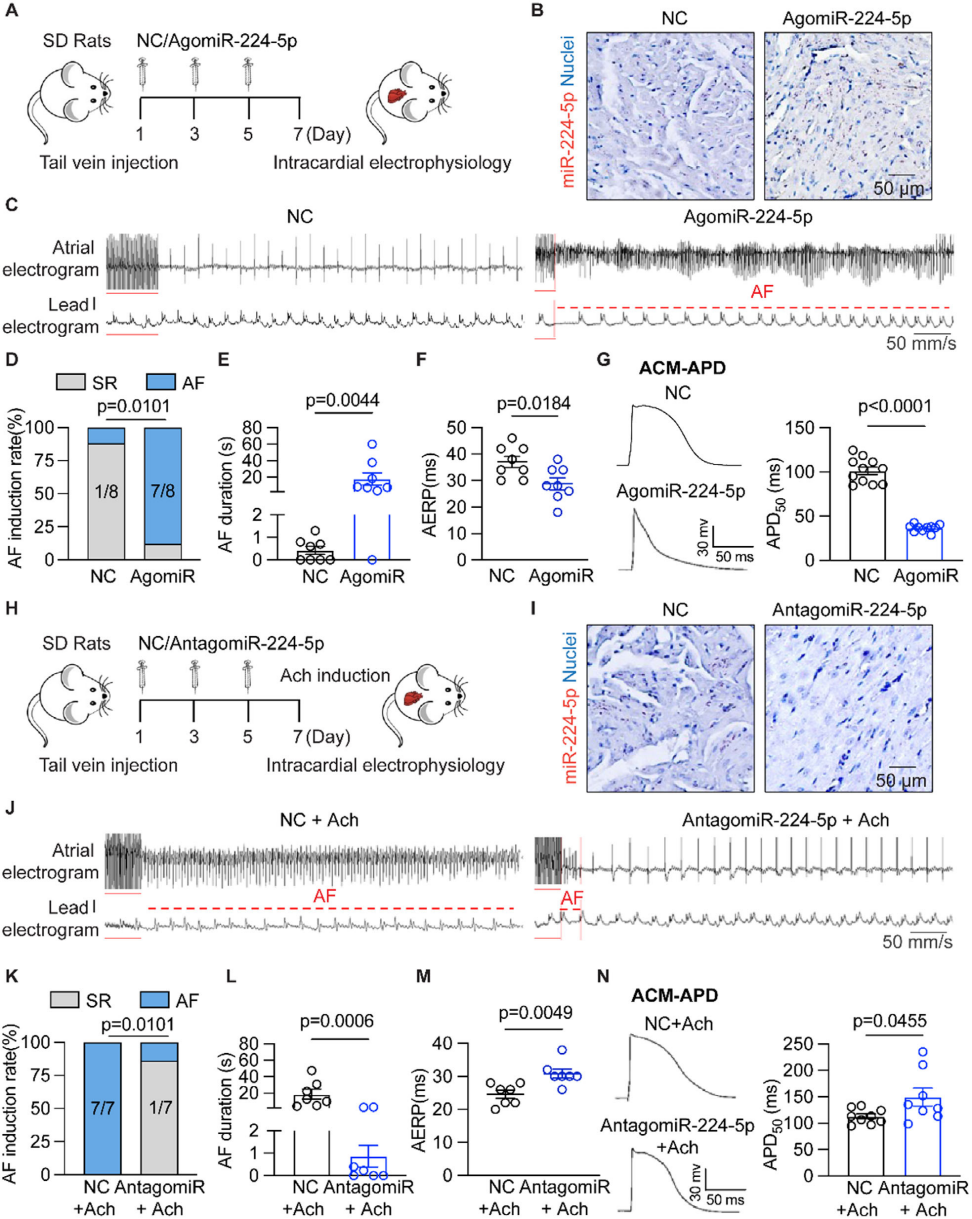
miR‐224‐5p promotes AF pathogenesis and abbreviated APD of atrial cardiomyocytes in rats. A) Scheme for negative control and agomiR‐224‐5p injection to SD rats, and AF induction test. B) Atrial samples labeled with miR‐224‐5p‐probe (red) and Nuclei probe (Blue) respectively (*n* = 3 per group). Scale bar: 50 µm. C–E) Representative traces of simultaneous recordings of surface ECG (lead I) and intracardiac electrograms in NC and AgomiR‐224‐5p rats after programmed intracardiac stimulation (red line), and incidence rate (*n* = 8 per group, *p* = 0.0101) and duration of AF (*n* = 8 per group, *p* = 0.0044). F) Quantification of AERP in NC and AgomiR‐224‐5p rats (*n* = 8 per group, *p* = 0.0184). G) Representative recordings of APD in isolated ACMs of NC and AgomiR‐224‐5p rats, and quantification of APD_50_ (*n* = 10 or 11 per group, *p* < 0.0001). H) Scheme for negative control and antagomiR‐224‐5p injection to SD rats, and AF induction test. I) Atrial samples labeled with miR‐224‐5p‐probe (red) and Nuclei probe (Blue) respectively (*n* = 3 per group). Scale bar: 50 µm. J–L) Representative simultaneous recordings of surface ECG (lead I) and intracardiac electrograms in NC and AntagomiR‐224‐5p rats with acetylcholine after programmed intracardiac stimulation (red line), and incidence rate (*n* = 7 per group, *p* = 0.0101) and duration of AF (*n* = 7 per group, *p* = 0.0006). M) Quantification of AERP in NC and AntagomiR‐224‐5p rats (*n* = 7 per group, *p* = 0.0049). (N) Representative recordings of APD in isolated atrial myocytes of NC and AntagomiR‐224‐5p rats and quantification of APD_50_ (*n* = 8 or 9 per group, *p* = 0.0455). NC, negative control; Ach, acetylcholine; AERP, atrial effective refractory period; APD, action potential duration. The bar graph data are mean±SEM with individual values. *p*‐values were determined with Fisher's exact test in D and K, and the Mann‐Whitney test in E and L. p‐values were determined with two‐tailed unpaired Student's *t*‐test in F, G, M, and N.

To explore the major effect of miR‐224‐5p on atrial arrhythmogenesis, we administered miR‐224‐5p agonist and agomiR‐224‐5p (50 mg kg^−1^ every other day, three times) to SD rats and performed the electrophysiological test (**Figure** **4**A). miR‐224‐5p expression was upregulated in atria and plasma of agomiR‐224‐5p treated rats (Figure S5A,B, Supporting Information). miR‐224‐5p accumulated in atrial myocytes of agomiR‐224‐5p treated rats as detected by rno‐miR‐224‐5p probe assay (Figure 4B). AgomiR‐224‐5p significantly increased AF incidence rate (87.5% versus 12.5%, *n* = 8, *p* = 0.0101, Figure 4C,D) and prolonged AF duration (*p* = 0.0044, Figure 4E). In addition, agomiR‐224‐5p treated rats performed an abbreviated atrial effective refractory period (AERP) (Figure 4F). Furthermore, action potential duration at 50% recovery (APD_50_) and 90% recovery (APD_90_) of isolated ACMs were markedly reduced in agomiR‐224‐5p treated rats compared with the NC group, as examined by whole‐cell patch clamp technique (Figure 4G; Figure S5D, Supporting Information).

We also evaluated the effects of miR‐224‐5p inhibitor on AF development by injecting antagomiR‐224‐5p (50 mg kg^−1^ every other day, three times) to 8‐week‐old rats. One week after injection, AF susceptibility of rats was examined by tachypacing in the presence of acetylcholine (Ach) (Figure 4H). The levels of miR‐224‐5p were reduced in atrial tissues and plasma of antagomiR‐224‐5p‐treated rats (Figure S5E,F, Supporting Information). The immunochemical study confirmed the reduction of miR‐224‐5p in the atria (Figure 4I). AntagomiR‐224‐5p attenuated the incidence of Ach‐induced AF (14.3% antagomiR‐224‐5p versus 100.0% NC, *p* = 0.0101, Figure 4J,K) and shortened AF duration (Figure 4L). AntagomiR‐224‐5p‐treated rats exhibited restored AERP compared with the NC group (Figure 4M). Moreover, the APD_50_ and APD_90_ of isolated ACMs were mildly prolonged in antagomiR‐224‐5p‐treated rats than NC controls (Figure 4N; Figure S5H, Supporting Information). These data indicated that miR‐224‐5p can contribute to AF development by inducing atrial electrical remodeling.

### 2.5 miR‐224‐5p Directly Targets CACNA1C in Atrial Myocytes

**Figure 5:**
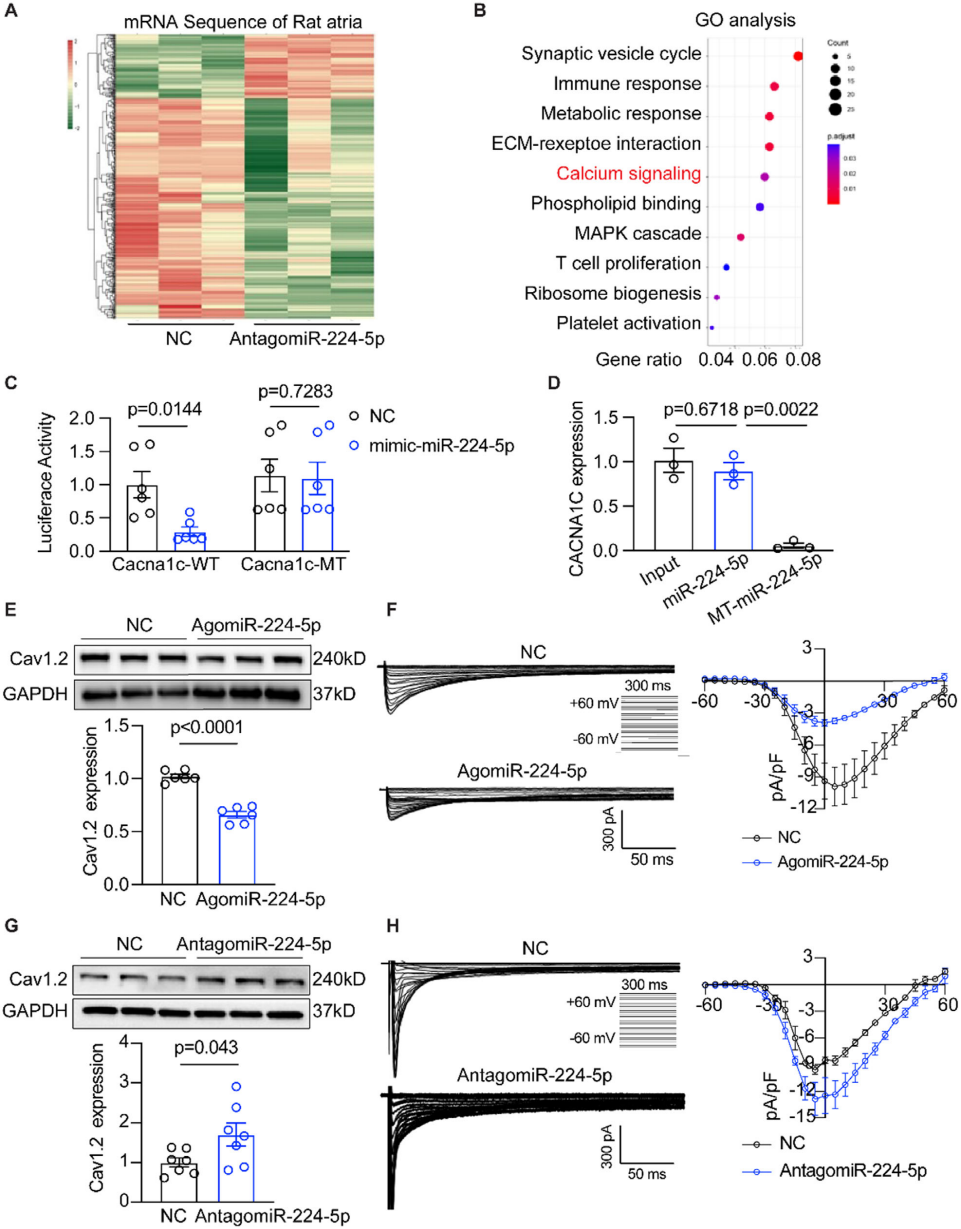
miR‐224‐5p directly targets CACNA1c mRNA in atrial cardiomyocytes. A) Heatmap of differential mRNAs in atria of NC and AntagomiR‐224‐5p rats (*n* = 3 per group). B) GO analysis of differentially expressed mRNAs. C) Luciferase activities of WT and MT after treatment with NC and mimics of miR‐224‐5p in cultured rat neonatal ACMs (*n* = 6 per group, NC vs mimic‐miR‐224‐5p *p* = 0.0144). D) RISK pulldown assay showing CACNA1C level in cultured rat neonatal ACMs treated with WT and MT miR‐224‐5p by qRT‐PCR test (*n* = 3 per group, Input vs miR‐224‐5p *p* = 0.6718, miR‐224‐5p vs MT‐miR‐224‐5p p = 0.0022). E) Representative Western blot and quantification of whole tissue Cav1.2 protein level in NC and AgomiR‐224‐5p rats (n = 6 per group, p<0.0001). F) Representative recordings and I‐V curve of *I*_Ca,L_ in isolated atrial cardiomyocytes of NC and AgomiR‐224‐5p rats (*n* = 4 or 7 per group). G) Representative Western blot and quantification of total whole tissue Cav1.2 protein level in NC and AntagomiR‐224‐5p rats (*n* = 7 per group, *p* = 0.043). H) Representative recordings and I‐V curve of *I*_Ca,L_ in isolated atrial myocytes of NC and AntagomiR‐224‐5p rats (*n* = 4 per group). The bar graph data are mean±SEM with individual values. *p*‐values were determined with two‐tailed unpaired Student's *t*‐test in C, E, F, G, and H. p‐values were determined with one‐way ANOVA and Turkey's multiple comparisons test in D.

To clarify the molecular mechanism by how miR‐224‐5p drives atrial electrophysiological disorders, we conducted mRNA sequencing of atria from rats treated with negative control or antagomiR‐224‐5p. The mRNA sequence showed that 449 genes were differentially expressed in atria between NC and antagomiR‐224‐5p groups (**Figure** **5**A). GO and KEGG analyses indicated that these differentially expressed genes were involved in the calcium signaling pathway (Figure 5B and Figure S6A, Supporting Information). As is known, L‐type calcium channel is a central molecule to affects APD of atrial cardiomyocytes. Hence, we blasted the nucleic acid sequence between mature miR‐224‐5p and CACNA1C, and found there were two matched binding sites in 3′UTR of *homo sapiens* and *rattus norvegicus* CACNA1C (Figure S6B, Supporting Information). Considering the score and duplicates of the binding region, we chose MRE2 and made a mutant plasmid to assess the direct combination between miR‐224‐5p and CACNA1C. We performed Luciferase reporter assay and found that miR‐224‐5p mimics significantly reduced the activity of primary rat neonatal cardiomyocytes transfected with wildtype CACNA1C plasmid but not in mutant Cacna1c plasmid, implying the binding ability of miR‐224‐5p on CACNA1C (Figure 5C). Moreover, we established biotin‐labeled rno‐miR‐224‐5p and mutant rno‐miR‐224‐5p and incubated them with rat cardiomyocyte extracts and captured them with streptavidin‐coated beads. qRT‐PCR results showed that native miR‐224‐5p successfully pulled down CACNA1C mRNA in rat cardiomyocyte extracts, while mutant miR‐224‐5p failed to target CACNA1C mRNA (Figure 5D). In the atria of the agomiR‐224‐5p rat, the CACNA1C mRNA and Cav1.2 protein were decreased compared to the NC rat (Figure 5E; Figure S6C, Supporting Information). Furthermore, agomiR‐224‐5p treatment resulted in a pronounced reduction of *I*_Ca,L_ current in the atrial cardiomyocytes (Figure 5F). In contrast, CACNA1C mRNA and protein levels were upregulated in the atria of antagomiR‐224‐5p‐treated rats (Figure 5G; Figure S6C, Supporting Information). Consistently, *I*_Ca,L_ current of atrial myocytes isolated from antagomiR‐224‐5p‐treated rats was larger than from control rats (Figure 5H). We also measured the mRNA levels of *HCN1*, *TRPC7*, *KCTD21*, *KCTD12*, and *KCNH1*, which are predicted as target genes of miR‐224‐5p in NC and agomiR‐224‐5p rat atria. qRT‐PCR results showed no significant changes in the above ion channels, though a decreased trend of *KCTD21* and *KCTD12* levels in agomiR‐224‐5p rat atria (Figure S6D, Supporting Information). These results explained that miR‐224‐5p can directly target CACNA1C and suppress *I*_Ca,L_ current to shorten APD of atrial cardiomyocytes.

### 2.6 Global miR‐224‐5p Overexpression Increases AF Susceptibility in Mice

To further clarify the effect of miR‐224‐5p on AF susceptibility, we generated global miR‐224‐5p knock‐in (*miR‐224‐5p^+/+^*) mice (Figure S7A, Supporting Information). qRT‐PCR data showed upregulated miR‐224‐5p level while unaltered pre‐miR‐224‐5p and miR‐224‐3p expression in *miR‐224‐5p^+/+^* mouse atria compared to the wild type (WT) group (Figure S7B–D, Supporting Information). The miR‐224‐5p probe assay verified that miR‐224‐5p was expressed in atrial tissues of *miR‐224‐5p^+/+^* mice than in WT mice (Figure S7E, Supporting Information) with no structural remodeling (Figure S7F, Supporting Information). Global miR‐224‐5p knock‐in mice exhibited significantly increased AF occurrence rate and maintenance duration (Figure S8A–C, Supporting Information), and shortened AERP (Figure S8D, Supporting Information) at 3‐month‐old age. The APD_50_ and APD_90_ of isolated ACMs were shortened in miR‐224‐5p knock‐in mice compared with WT control (Figure S8E–G, Supporting Information). Furthermore, miR‐224‐5p overexpression attenuated *I*_Ca,L_ current of isolated atrial myocytes, and Cav1.2 protein level in atrial tissues compared to WT (Figure S8H,I, Supporting Information).

### 2.7 Cardiac Fibroblasts Specific miR‐224‐5p Knock‐In Enhances AF Inducibility In Vivo

**Figure 6:**
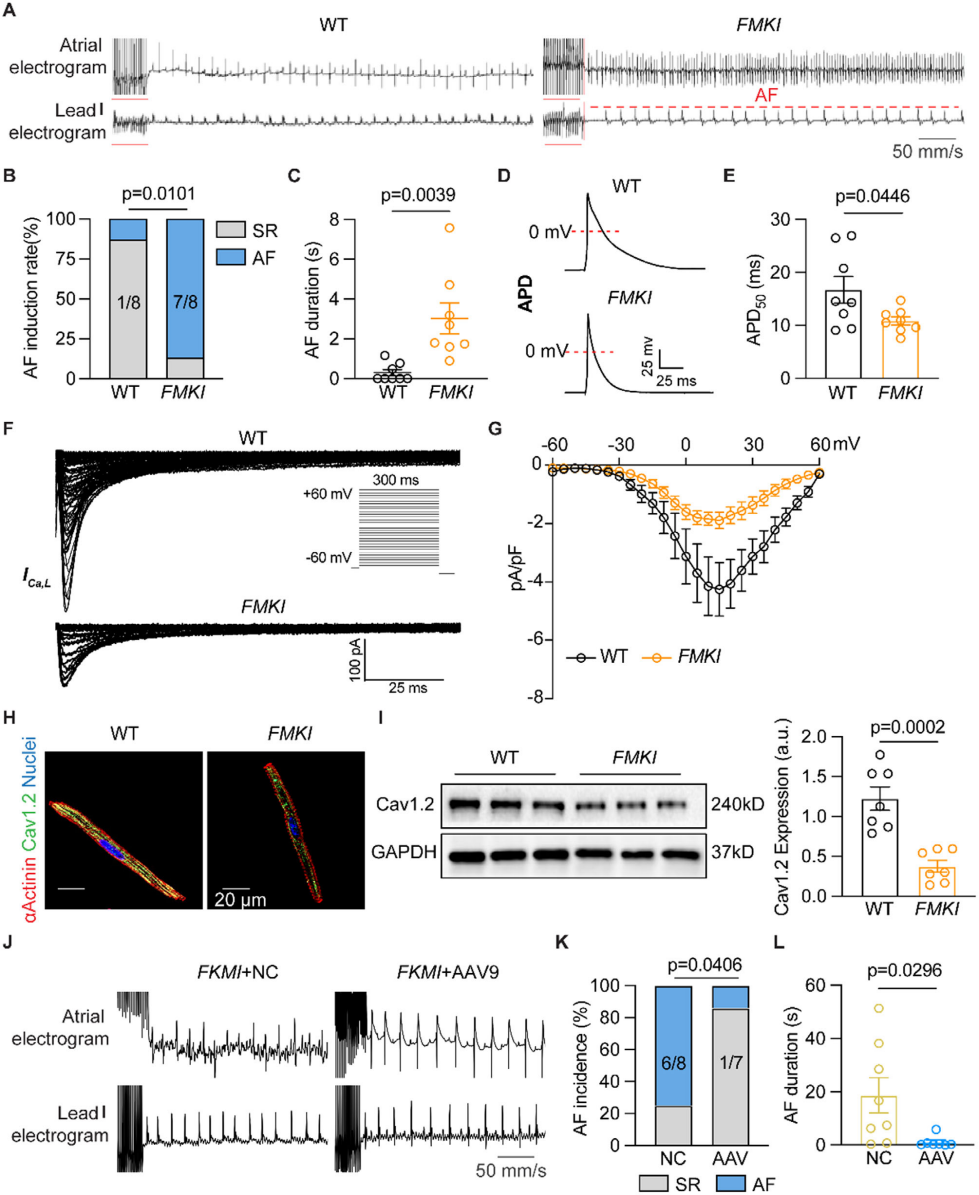
Fibroblasts‐specific miR‐224‐5p knock‐in increases AF susceptibility in mice. A–C) Representative traces of simultaneous recordings of surface ECG (lead I) and intracardiac electrograms in WT and *FMKI* mice after programmed intracardiac stimulation (red line), and incidence rate (*n* = 8 per group, *p* = 0.0101) and AF duration (*n* = 8 per group, *p* = 0.0039). D and E) Representative recordings of APD and quantification of APD_50_ in isolated atrial cardiomyocytes of WT and *FMKI* mice (*n* = 8 per group, *p* = 0.0446). F and G) Representative recordings and I‐V curve of *I*_Ca,L_ in isolated atrial cardiomyocytes of WT and *FMKI* mice (*n* = 8 per group). H) Isolated atrial cardiomyocytes stained with Cav1.2 (green), α‐Actinin (red), and DAPI (Blue) respectively (*n* = 3 per group), Scale bar: 20 µm. I) Western blot testing Cav1.2 protein level in the atrium of WT and *FMKI* mice (*n* = 7 per group, *p* = 0.0002). J–L) Representative traces of simultaneous recordings of surface ECG (lead I) and intracardiac electrograms in *FMKI* treated AAV9 virus mice after programmed intracardiac stimulation (red line), and incidence rate (*n* = 7 or 8 per group, *p* = 0.0406) and AF duration (*n* = 7 or 8 per group, *p* = 0.0296). The bar graph data are mean±SEM with individual values. *p*‐values were determined with Fisher's exact test in D and K, and the Mann‐Whitney test in E and L. p‐values were determined with a two‐tailed unpaired Student's *t*‐test in E and I.

To further clarify the effects of fibroblast‐derived exosome miR‐224‐5p on atrial electrophysiology, we established fibroblast‐specific miR‐224‐5p knock‐in (*FMKI*) mice by crossbreeding miR‐224‐5p flox/flox mice with TCF21‐Cre mice (MEF2C) (Figure S9A, Supporting Information). The miR‐224‐5p probe assay and qRT‐PCR verified that the level of mature miR‐224‐5p was higher in isolated ACFs, not ACMs, in *FMKI* mice than in WT with unchanged expressions of pre‐miR‐224‐5p and miR‐224‐3p (Figure S9B,F–H, Supporting Information). There was no significant change in the atrial myocytes fraction and fibrosis accumulation between the two groups of mice (Figure S9C,D, Supporting Information). Echocardiography data suggested comparable LVEF and LA area between WT and *FMKI* mice (Figure S9E, Supporting Information). However, the AF incidence was higher and AF duration was longer in *FMKI* mice than WT mice (**Figure** **6**A–C). Moreover, the AERP of mice atria, and APD_50_ and APD_90_ of isolated atrial cardiomyocytes in *FMKI* mice were markedly shortened (Figure 6D,E; Figure S9I,J, Supporting Information). Enhanced fibroblast‐specific miR‐224‐5p expression also diminished *I*_Ca,L_ current in isolated ACMs in *FMKI* mice (Figure 6F,G). We found that the expression of Cav1.2 protein was reduced in isolated ACMs of *FMKI* mice compared to the WT group via immunofluorescence staining and western blot test (Figure 6H,I).

Then we subjected *FMKI* mice either to AAV9 negative control (*FMKI*+NC) or AAV9‐miR‐224‐5p sponge (*FMKI+*AAV9) to observe whether suppressing miR‐224‐5p only in cardiac fibroblasts can affect AF pathogenesis (Figure S10A, Supporting Information). Echocardiography was performed to show no dysfunction of left ventricle ejection fraction and unchanged LA size between NC and AAV9‐miR‐224‐5p sponge injected mice (Figure S10B–E, Supporting Information). The weight of the atria and ventricles was comparable between *FMKI*+NC and *FMKI+*AAV9 mice (Figure S10F, Table S19, Supporting Information). qRT‐PCR results demonstrated that the level of miR‐224‐5p was downregulated in both isolated ACFs and their secreted exosomes from *FMKI+*AAV9 mice compared to *FMKI*+NC group (Figure S10G, Supporting Information). Surprisingly, AAV9‐miR‐224‐5p sponge virus significantly reduced PES‐induced AF incidence and AF duration with restored AERP compared to *FMKI*+NC mice (Figure 6J–L; Figure S10H, Supporting Information). Besides, we found upregulated CACNA1C and Cav1.2 protein levels in the isolated ACMs from *FMKI+*AAV9 mice (Figure S10I,J, Supporting Information). These findings revealed that cardiac fibroblasts‐specific miR‐224‐5p participates in the development of atrial fibrillation via inhibiting L‐type calcium channel of atrial cardiomyocytes, independent of ventricular and atrial structure change.

### 2.8 Silencing CFs‐Specific miR‐224‐5p Ameliorated AF Pathogenesis in Multiple Chronic AF Models

**Figure 7:**
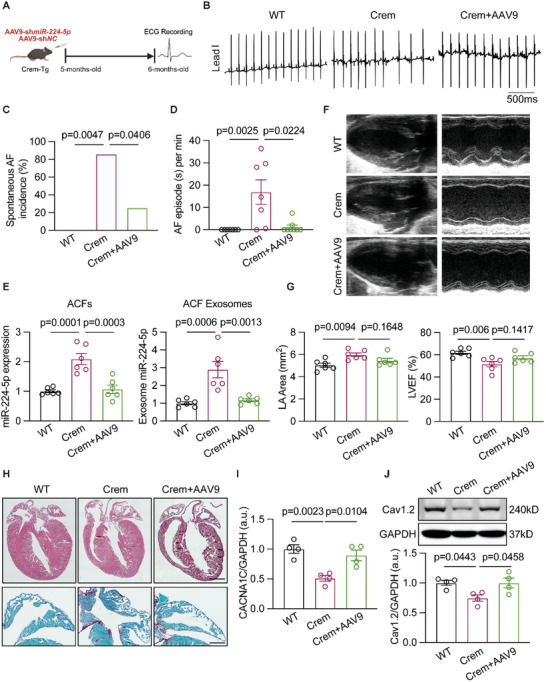
Knockdown of CFs‐miR‐224‐5p attenuated spontaneous AF incidence in Crem mice. A) Study design for Crem mice injected with NC or AAV9‐sh*miR‐224‐5p*. B) Representative surface ECG recordings in WT, Crem, and Crem+AAV9 mice. C and D) Spontaneous AF incidence rate (*n* = 7 or 8 per group, WT vs Crem *p* = 0.0047, Crem vs Crem+AAV9 *p* = 0.0406) and AF episode duration (*n* = 7 or 8 per group, WT vs Crem *p* = 0.0025, Crem vs Crem+AAV9 *p* = 0.0224). E) miR‐224‐5p expression of isolated ACFs (*n* = 6 per group, WT vs Crem *p* = 0.0001, Crem vs Crem+AAV9 *p* = 0.0003) and ACFs‐derived exosomes from mice (*n* = 6 per group, WT vs Crem *p* = 0.0006, Crem vs Crem+AAV9 *p* = 0.0013). F and G) Representative echocardiography images, quantification of LA area (*n* = 6 per group, WT vs Crem *p* = 0.0094, Crem vs Crem+AAV9 *p* = 0.1648) and LVEF (*n* = 6 per group, WT vs Crem *p* = 0.006, Crem vs Crem+AAV9 *p* = 0.1417) in WT, Crem and Crem+AAV9 mice. H) HE (up) and Picrosirius staining (down) of the whole heart in three groups of mice, Scale bar: 1 mm; Scale bar: 0.5 mm. I) qRT‐PCR testing CACNA1C expression in the left atrium of WT, Crem, and Crem+AAV9 mice (*n* = 4 per group, WT vs Crem *p* = 0.0023, Crem vs Crem+AAV9 *p* = 0.0104). J) Western blot testing Cav1.2 protein level in the left atrium of WT Crem and Crem+AAV9 mice (*n* = 4 per group, WT vs Crem *p* = 0.0443, Crem vs Crem+AAV9 *p* = 0.0458). The bar graph data are mean±SEM with individual values. *p*‐values were determined with Fisher's exact test in C, and the Mann‐Whitney test in D. p‐values were determined with two‐tailed unpaired Student's *t*‐test in E, G, I, and J.

To verify the exact role of CFs‐specific miR‐224‐5p in the development of chronic AF, we conducted a spontaneous AF mouse model, CREM‐IbΔC‐X transgenic (Crem) with injection of adeno‐associated virus 9 (AAV9)‐hTCF21 system encoding miR‐224‐5p sponge to silence its expression in a cardiac fibroblast manner (**Figure** **7**A). Surface ECG recording for mice under 36.0 to 36.5 °C body temperature was performed to analyze spontaneous AF episodes. The incidence of spontaneous AF in Crem mice was much higher (Figure 7B,C), accompanied by longer AF episodes (Figure 7D) than the WT and Crem+AAV9 groups of mice. The expressions of miR‐224‐5p in ACFs and its derived exosomes from Crem mice were elevated compared to WT mice, which was reversed by AAV9‐hTCF21 miR‐224‐5p sponge (Figure 7E). Then, echocardiography data demonstrated a larger LA area with decreased LVEF of Crem mice compared to WT mice, which showed a mild trend of improvement in Crem+AAV9 mice (Figure 7F,G). Picrosirius staining images represented more fibrotic accumulation in the atria of Crem mice than WT and Crem+AAV9 mice (Figure 7H). Furthermore, CACNA1C and Cav1.2 protein expressions were decreased in the atria of Crem mice compared to WT, which was restored by the treatment of AAV9‐hTCF21 miR‐224‐5p sponge (Figure 7I,J).

Then, we established a 4‐week treatment of Ang II‐induced AF mouse model with either AAV9‐NC or AAV9‐hTCF21 system encoding miR‐224‐5p sponge (Figure S11A, Supporting Information). Echocardiography results confirmed a larger LA area of Ang II mice compared to WT mice, which showed a mild trend of improvement in Ang II+AAV9 mice (Figure 11B,C, Supporting Information). Ang II‐treated mice suffered an enhanced incidence of inducible AF with longer AF duration compared to WT and Ang II+AAV9 group (Figure S11D–F, Supporting Information). qRT‐PCR assay verified elevated miR‐224‐5p expression in both ACFs and their derived exosomes from Ang II‐treated mice compared to WT mice, which was reversed by AAV9‐hTCF21 miR‐224‐5p sponge (Figure S11G, Supporting Information). Masson staining revealed atrial fibrosis in Ang II‐treated mice, which was mildly alleviated by inhibiting miR‐224‐5p of ACFs (Figure S11H, Supporting Information). We also observed a restored Cav1.2 protein level in the atria of Ang II+AAV9 mice compared to Ang II‐treated mice (Figure S11I, Supporting Information).

Additionally, we subjected antagomiR‐224‐5p to a spontaneous hypertension rat (SHR) for 4 weeks to observe whether inhibition of miR‐224‐5p could attenuate chronic hypertension‐associated AF pathogenesis (Figure S12A, Supporting Information). The blood pressure increased in SHR compared to control rats, but was comparable to SHR+AntagomiR‐224‐5p rats (Figure S12B, Supporting Information). The incidence of inducible AF in the SHR group significantly enhanced with longer AF duration compared to control and antagomiR‐224‐5p‐treated rats (Figure S12C–E, Supporting Information). qRT‐PCR assay confirmed upregulated miR‐224‐5p expression in both isolated ACFs and their secreted exosomes from SHR rats, which was attenuated by antagomiR‐224‐5p (Figure S12F, Supporting Information). Masson staining revealed more atrial fibrosis in SHR rats, which was not reversed by antagomiR‐224‐5p treatment (FigureS12G, Supporting Information). Besides, we observed decreased CACNA1C mRNA and Cav1.2 protein expression in the atria of the SHR group, which was restored by antagomiR‐224‐5p treatment (Figure S12H,I, Supporting Information). All these data suggested that effectively suppressing CFs‐specific miR‐224‐5p attenuates both spontaneous and inducible AF phenotype in the different chronic mouse models.

### 2.9 Plasma Exosomes from AF Patients were Enriched with miR‐224‐5p and Exacerbated AF Susceptibility in Rats

**Figure 8:**
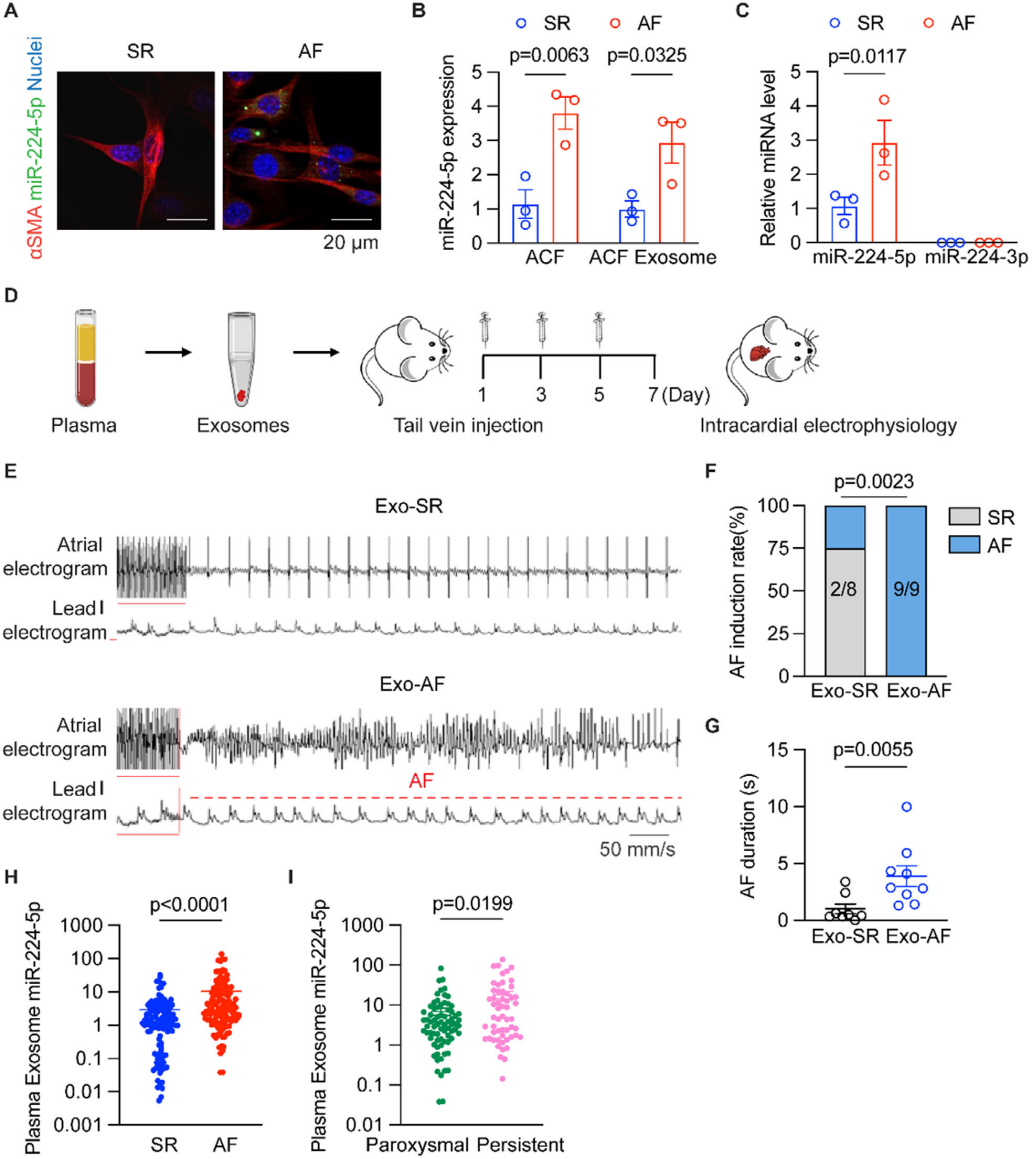
Plasma exosomes from AF patients are enriched with miR‐224‐5p and exacerbate AF susceptibility in rats. A) Representative IF images of isolated primary atrial fibroblasts from patient right atria staining with miR‐224‐5p (green), α‐SMA (red), and DAPI (Blue), respectively (*n* = 3 per group), Scale bar: 20 µm. B) qRT‐PCR test of miR‐224‐5p expressions in isolated primary human ACFs (*n* = 3 per group, *p* = 0.0063) and their released exosomes (*n* = 3 per group, *p* = 0.0325). (C) qRT‐PCR test of miR‐224‐5p and miR‐224‐3p expressions in the circulating exosomes obtained from SR and AF patients (*n* = 3 per group, *p* = 0.0117). D) Scheme for Exo‐SR and Exo‐AF transferred SD rats, and AF induction test. E) Representative traces of simultaneous recordings of surface ECG (lead I) and intracardiac electrograms in Exo‐SR and Exo‐AF rats after programmed intracardiac stimulation (red line). F) Incidence rate of pacing‐induced AF occurrence and AF duration in Exo‐SR and Exo‐AF rats (*n* = 8 or 9 per group, *p* = 0.0023). G) AF duration in Exo‐SR and Exo‐AF rats (*n* = 8 or 9 per group, *p* = 0.0055). H) Circulating levels of exosome miR‐224‐5p in SR and AF patients (SR *n* = 125, AF *n* = 130, *p* < 0.0001). I) Plasma levels of exosome miR‐224‐5p in paroxysmal and persistent AF patients (paroxysmal AF *n* = 74, persistent AF *n* = 56, *p* = 0.0199). The bar graph data are mean±SEM with individual values. *p*‐values were determined with Fisher's exact test in F, and the Mann‐Whitney test in G and I. p‐values were determined with two‐tailed unpaired Student's *t*‐test in B, C, and H.

We further obtained atrium and plasma samples from AF and sinus rhythm (SR) patients to examine miR‐224‐5p levels in circulating exosomes, isolated ACFs, and their secreted exosomes. The miR‐224‐5p probe assay suggested more miR‐224‐5p expression in the isolated ACFs from AF patients than SR controls (**Figure** **8**A), not accumulating in either CD64^+^ macrophages or CD31^+^ endothelial cells of AF patient atria (Figure S13A–D, Supporting Information). qRT‐PCR results demonstrated a higher level of miR‐224‐5p in both ACFs and exosomes derived from ACFs of the AF group compared to the SR group, while unaltered miR‐224‐3p level (Figure 8B; Figure S14A, Supporting Information). We also found parallel increased miR‐224‐5p expression, not miR‐224‐3p, of circulating exosomes in the above AF patients (Figure 8C). Then we explored the effect of plasma exosomes from AF patients on AF susceptibility in rats. All these donors’ information was shown in Table S2 (Supporting Information). There were no substantial differences in age, BMI, other healthy history, and cardiac function between the two groups of donors. The extracted exosomes (5×10^8^–1×10^9^ particles) from sinus rhythm patients (Exo‐SR) and AF patients (Exo‐AF) were transfused into rats via tail injection every other day for three times. Intracardiac AF induction was performed by right atrial burst pacing (Figure 8D). PKH‐26 label assay showed that transferred exosomes entered into atrial tissue (Figure S14B, Supporting Information). Circulating exosomes from AF patients significantly increased the occurrence of AF and prolonged AF duration (Figure 8E–G), and shortened AERP (Figure S14C, Supporting Information). The expression of Cav1.2 protein decreased in Exo‐AF rat atria (Figure S14D,E, Supporting Information).

To validate whether the miR‐224‐5p level of plasma exosomes correlates with AF occurrence and maintenance, we collected peripheral blood samples from SR and AF patients (Table S1, Supporting Information). qRT‐PCR analysis confirmed upregulated exosome miR‐224‐5p in the AF group compared to the SR group (Figure 8H). We also compared circulating exosome miR‐224‐5p levels between paroxysmal and persistent AF patients and observed higher miR‐224‐5p levels of circulating exosomes in the persistent AF group (Figure 8I). Moreover, the expression of miR‐224‐5p in plasma exosomes was upregulated in AF recurrence patients with radiofrequency catheter ablation at baseline (Figure S14F, Supporting Information) and performed higher level after 1 year follow‐up compared to non‐AF recurrence patients (Figure S14G, Supporting Information). These data clarified that circulating exosome miR‐224‐5p was able to increase AF susceptibility in vivo and was correlated with AF progression in the patients.

## 3 Discussion

In this study, we discovered that upregulated exosomes miR‐224‐5p derived from Ang II‐induced ACFs play a causal role in the development of atrial arrhythmogenesis. Enhanced miR‐224‐5p leads to atrial electrical remodeling and increases the incidence of AF by inhibiting CACNA1c expression, which diminishes *I*_Ca,L_ current in atrial cardiomyocytes. The above findings provide new pathophysiological insights that CF‐secreted exosomes can induce shortened APD in atrial cardiomyocytes and contribute to AF pathogenesis.

CFs account for 45%–55% of the cell population in atria and are responsible for maintaining the extracellular matrix production.^[^ ^20^ ^]^ In addition to mechanical interaction and electrical coupling between CFs and CMs, emerging studies have clarified the effects of paracrine signaling within the local extracellular environment.^[^ ^7^, ^21^ ^]^ Among these, exosomes are well recognized as a crucial mediator without cell‐to‐cell contact by transferring biological contents between these two neighboring cells.^[^ ^22^, ^23^, ^24^ ^]^ CFs‐secreted exosomes can augment the production of Ang II by regulating MAPK and Akt pathway, which lead to pathological cardiac hypertrophy.^[^ ^15^ ^]^ Another independent study has identified a specific role of miR‐21*‐enriched CF exosomes can be taken up by neighboring cardiomyocytes, resulting in the reduced expression of SORBS2 and PDLIM5, and eventually contributing to cardiac hypertrophy.^[^ ^17^ ^]^ However, it is unknown whether exosomes derived from ACFs play a role in AF pathogenesis. Our study revealed that exosomes secreted from Ang II induced primary human and adult rat ACFs significantly enhanced AF vulnerability, suggesting an essential role for ACF‐exosomes in promoting AF pathogenesis. Our data clarified the association between ACF‐derived exosomes and the development of atrial arrhythmia.

Extracellular vesicles, especially for exosome content, vary a lot according to various cell types and disease status.^[^ ^25^ ^]^ Under stress conditions, cardiac fibroblasts can produce dangerous molecular patterns such as cytokines, damaged DNAs, RNAs, and ncRNAs.^[^ ^26^, ^27^ ^]^ miRNAs are the most common biological cargoes loaded into exosomes. Much literature has addressed the arrhythmogenic role of miRNAs such as miR‐1, miR‐26, miR‐21 and etc. in the pathophysiological contribution of AF and their potential therapeutic implications.^[^ ^28^, ^29^ ^]^ Here, we found that ACF‐derived exosome miRNA is the major molecular component promoting AF development via performing a knockdown Dicer assay. Then microRNA sequencing assay identified that miR‐224‐5p increased in both ACFs and ACF‐derived exosomes under Ang II stimuli. Previous studies also supported an upregulation of miR‐224‐5p in atrial appendages of AF patients.^[^ ^30^ ^]^In the current study, a much lower basic level of miR‐224‐5p in ACMs than ACFs and unaltered miR‐224‐3p expression in Ang II‐treated atrial cardiomyocytes were observed. The above data suggested that upregulated miR‐224‐5p of atrial myocytes should originate from ACFs during different stress conditions. There is some evidence showing that cancer‐associated fibroblasts‐derived exosomes loaded with miR‐224‐5p can enter and induce the malignant behaviors of different cancer cells.^[^ ^31^, ^32^ ^]^ These findings are consistent with our results that ACFs‐derived miR‐224‐5p serves as a paracrine factor to modulate the physiological function of neighboring cells. Nevertheless, it is not excluded that other miRNAs contained in ACFs‐derived exosomes have effects on atrial arrhythmogenesis.^[^ ^33^ ^]^ Future study is needed to examine the potential function of other miRNAs in the context of atrial arrhythmogenesis. Besides, there are other types of cells, such as endothelial cells, immune cells, and epicardium in the atria microenvironment, which might secrete miR‐224‐5p to affect cardiomyocyte function. Though our data did not show differential expression of miR‐224‐5p in macrophages and endothelial cells under AF conditions, it would be better to perform single‐cell miRNA sequencing of AF patient atria to clarify miR‐224‐5p expression in different cell populations.

miR‐224‐5p has been reported to facilitate cell death by regulating non‐exciting cells, such as immune cells, tumor cells, and epithelial cells.^[^ ^34^, ^35^, ^36^ ^]^ We for the first time provided knowledge of the important role of miR‐224‐5p in regulating the electrophysiological activity of atrial myocytes. Our work clarified that miR‐224‐5p secreted from Ang II‐induced ACFs can trigger AF in a paracrine manner. Furthermore, we found that miR‐224‐5p directly bound to CACNA1C and inhibited its expression. The amplitude of *I*_Ca,L_ current attenuated in atrial myocytes of both global and fibroblast‐specific miR‐224‐5p knock‐in mice. ACFs‐derived exosome miR‐224‐5p suppressed the mRNA expression of L‐type calcium channel subunit, CACNA1C. In consistent, previous research has revealed that exposure of cardiomyocytes to myofibroblast‐conditioned medium contributed to abnormal ion channel protein expression, reduced amplitude of calcium transients, and shortened APD in atrial cardiomyocytes.^[^ ^37^ ^]^ Our data prove that upregulated miR‐224‐5p leads to shortened AERP and initiates AF maintenance mainly dependent on suppressing CACNA1C without causing atrial structure remodeling in both acute exosome transferred and multiple chronic AF (including spontaneous AF, angiotensin II, and chronic hypertension‐associated) animal models. Emerging evidence has reported that miR‐224‐5p inhibits the NLRP3 signaling pathway to alleviate inflammatory responses.^[^ ^38^, ^39^ ^]^ In the current study, there was a lack of inflammatory foci in the atria of the acute exosome transferred rat model and fibroblasts specific miR‐224‐5p knock‐in (*FMKI*) mice, which was consistent with previous studies. This may explain why miR‐224‐5p has negative effects on inflammation and causes programmed cell death, such as pyroptosis, in cardiomyocytes.

A variety of research reveals that several dysregulated potassium channels can result in shortening of APD associated with AF pathogenesis.^[^ ^40^ ^]^ In the current study, we examined the mRNA levels of SCN5A, KCNA5, KCNJ2, and KCNJ5 in the atria of acute agomiR‐224‐5p treated rats and found no significant change in these ion channels compared to negative control rats. However, the indirect effect of miR‐224‐5p on other ion channels cannot be excluded in a chronic animal model. It is of great interest to investigate other potential mechanisms of ACFs‐derived exosome miR‐224‐5p involved in AF‐related electrical remodeling. Based on these clues, it is supposed that specifically interfering with ACFs‐derived exosome miR‐224‐5p might be a potential therapeutic target for some AF patients without severe atrial structural alteration. It is of great interest to evaluate whether miR‐224‐5p only affects L‐type calcium channels without leading to any changes in sodium and potassium ion channels in a future study to validate the safety of this approach.

Finally, we evaluated the expressions of plasma and ACF‐derived exosome miR‐224‐5p in patients with or without AF. Increased exosome miR‐224‐5p expressions of atrial fibroblasts and plasma from AF individuals compared to SR individuals indicated a paralleled correlation. A recent study reported that miR‐224‐5p was increased in circulating EVs of patients with reduced coronary flow reserve.^[^ ^41^ ^]^ Though we cannot totally confirm that atrial fibroblast is the only origin of miR‐224‐5p in circulating EVs in AF patients, our findings suggest a potential generation of exosome miR‐224‐5p that came from atrial fibroblasts. Another research indicates a decreased profile of miRNAs in the left atria of AF patients exhibiting the transition from paroxysmal AF to permanent AF.^[^ ^42^ ^]^ However, we observed a trend of enhanced levels during the transition from paroxysmal AF to persistent AF, which could be attributed to a potential secretion of miR‐224‐5p from activated fibroblasts. It would be better to perform further studies to compare the cell proportion of subtype fibroblasts and their miR‐224‐5p expressions in the atria of paroxysmal, persistent, and permanent AF patients. In addition, we observed increased miR‐224‐5p in circulating exosomes from patients who suffered recurrent AF compared to nonrecurrent patients who underwent catheter ablation and showed a still higher miR‐224‐5p level after ≈1 year of follow‐up. This data provides potential translational value of circulating miR‐224‐5p in monitoring AF recurrence after catheter ablation.^[^ ^43^, ^44^ ^]^ Afterward, we transferred human plasma exosomes into rat model as a conserved binding region between miR‐224‐5p and CACNA1C in *homo sapiens* and *rattus norvegicus* to examine whether enhanced plasma exosome miR‐224‐5p from AF patients can trigger AF. Whereas other organs such as the liver, kidney, and colon can also secrete miR‐224‐5p carried exosomes into circulation, which may have an effect on the exact value of circulating exosome miR‐224‐5p, our data indicated that CFs‐derived exosome miR‐224‐5p is at least one of the major origins and regulators. Overall, our findings proved that plasma exosomes from AF patients exacerbated atrial arrhythmia susceptibility in vivo as well and pointed out a potential direction that the circulating exosome miR‐224‐5p level is associated with the prognosis of AF patients and recurrence after catheter ablation.

The study has certain limitations. First, as MEF‐Cre is expressed in global fibroblasts,^[^ ^45^ ^]^ increased miR‐224‐5p expression may impact both atrial and ventricular functions in the MEF2C‐Cre miR‐224‐5p knock‐in mice. Based on our findings, the atrial structure and systolic function of the left ventricle were not affected by MEF2C‐Cre miR‐224‐5p knock‐in. Though the adeno‐associated virus 9 (AAV9)‐hTCF21 system encoding miR‐224‐5p sponge for cardiac fibroblasts was used in our current study, an atrial fibroblast‐specific adeno‐associated virus would be more optimal to be explored and performed to assess the role of miR‐224‐5p in AF development. Second, our present work used commercial primary human ACFs to establish vitro model and transferred the exosomes to the rat model. The clinical characteristics of the donors cannot be provided. Though we tried to isolate ACFs from clinical human right atria and found upregulated miR‐224‐5p expression of ACFs in AF patients. Due to the limited number of patient atrial samples available for collecting enough secreted exosomes, a follow‐up study is needed to determine whether ACF‐derived exosomes obtained from AF patients can induce AF in vivo. Third, despite restored Cav1.2 protein level and *I*_Ca_ current shown in antagomiR‐224‐5p‐treated rat atria, APD_90_ did not exhibit total rescue in rat ACMs. This could be attributed to the changes in other APD‐related ion channels, which were caused by pre‐Ach treatment.^[^ ^46^ ^]^ Finally, we do not think that our current finding is a very universal pathophysiological mechanism that can explain the arrhythmogenesis of every risk factor associated with AF. It would be better to confirm the correlation between different risk factors associated with AF and ACFs‐derived exosome miR‐224‐5p level.

In conclusion, our work establishes a crucial role for atrial fibroblast‐derived exosome miR‐224‐5p in atrial arrhythmogenesis. The paracrine manner of atrial fibroblasts related signaling through exosomes contributes to atrial electrical remodeling and exacerbates AF susceptibility. These new findings may provide a potential therapeutic approach of exosomes to attenuate atrial arrhythmogenesis.

## 4 Experimental Section

### Data Availability

All the original data and methods in this study are available from the corresponding authors upon reasonable request. Detailed information and descriptions of all materials are provided in the Supporting Information.

### Statistical Analysis

All data sets were tested for normality with the D'Agostino test and the Shapiro‐Wilk test. The quantitative data were expressed as the mean±SEM. The sample size (*n*) for each statistical analysis was provided in the *Figure Legends*. Two‐tailed unpaired Student's *t* tests were applied for comparisons in two groups, maintaining normal distributions. Mann‐Whitney and Kruskal‐Wallis tests followed by Dunn's test, were used to compare groups when the data were not normally distributed, and the quantitative data were expressed as median with IQR. Fisher's exact or Chi‐squared tests were used to compare categorical data. Multiple group comparisons were assessed through one‐way ANOVA and Tukey's multiple comparison tests. Two‐way ANOVA analysis with multiple group comparisons (Bonferroni‐corrected Student *t* tests) was used to evaluate two or more main effect factors. Statistical analysis was performed with software GraphPad Prism 10. *p‐*value < 0.05 was considered statistically significant.

### Patients Enrolled in the Study

All patients in this study were recruited from the Department of Cardiology, the First Affiliated Hospital in Harbin Medical University, as a single institution from September 2019 to March 2024. This study has been approved and registered by the Ethics Committee in the First Affiliated Hospital of Harbin Medical University (No. 201868). Informed consent was obtained from all participants. AF was defined as the occurrence of atrial arrhythmia with proven electrogram with the age of more than 18‐years‐old. The AF group contained paroxysmal and persistent AF patients, but no permanent AF subtype. Patients attending the cardiology department for non‐AF‐related conditions were screened and selected to create a sinus rhythm (SR) group. SR controls were age‐ and sex‐matched with the diseased group among consecutive participants in the same time period. Basic clinical information, including age, gender, body mass index (BMI), cardiovascular disease risk factors, medical history, smoking status, comorbid conditions, and drugs, was recorded. There were some patients excluded from the SR group when they had any previous AF or other arrhythmia (including supraventricular tachycardia and ventricular arrhythmia) history. The individuals were excluded if they had blood disease, chronic infection, severe chronic kidney disease (eGFR<30 mL/min^−1^), recent or active tumor, thyroid disease, acute myocardial infarction, acute heart failure, heart transplant, coronary artery bypass surgery, cardiomyopathy (arrhythmogenic right ventricular dysplasia, hypertrophic cardiomyopathy, and dilated cardiomyopathy and takotsubo cardiomyopathy), pericarditis and myocarditis. Venous blood samples of all participants with 12‐h fasting were collected on the next morning from admission to the hospital by various tubes. Hematological parameters, including blood routine examination, biochemical indicators, and blood coagulation function, were measured via an automatic analyzer, respectively. Besides, the level of plasma exosome miRNA‐224‐5p was detected by qRT‐PCR. The characteristics of all enrolled individuals were listed in Table S1 (Supporting Information). Left atrial tissues of 10 SR and 10 AF patients were harvested during the cardiac surgery and digested with Liberase TH (Roche, Basel, Switzerland) to obtain the isolated human atrial fibroblasts to perform further experiments. The characteristics of above above‐enrolled individuals were listed in Table S2 (Supporting Information).

### Experimental Animals

All animal experiments were approved by the Institutional Animal Care and Use Committee at the First Affiliated Hospital of Harbin Medical University. Male Sprague Dawley (SD) rats (10–12 weeks old) were housed in separate cages in a temperature‐controlled room (22–24 °C) with free access to water and food. All SD rats were obtained from Beijing Charles River Laboratory Animal Co. Ltd. Control Wistar Kyoto (WKY) and spontaneous hypertension rats (SHR) were purchased from Beijing Charles River Laboratory Animal Co. Ltd. Male rats of matched age were included and administered with agomiR‐224‐5p/antagomiR‐224‐5p and negative control in the study. miR‐224‐5p homozygous knock‐in mice (*miR‐224‐5p^+/+^*) and fibroblast‐specific miR‐224‐5p knock‐in (*FMKI*) mice with C57/BL6 mouse background were established by Cyagen Biosciences Inc. CREM‐IbΔC‐X transgenic (Crem) mice with C57/BL6 mouse background were established. C57/BL6 wild‐type (WT) mice and genetically modified mice were housed in a specific pathogen‐free (SPF) environment. Male and female WT, *miR‐224‐5p^+/+,^* and *FMKI* mice with matched age (10–12 weeks old) were included in the current study. Male and female WT and Crem mice of matched age (6 months old) were used in the current study.

### Isolation, Purification, and Identification of Exosomes

The methods of isolation, separation, and purification in exosomes were guided by recent protocols.^[^ ^47^ ^]^ Briefly, medium and plasma samples were centrifuged at 300×g for 10 min, 2 000×g for 30 min, and 10 000×g for 60 min. Then the supernatant was followed by filtration through a 0.22 µm filter to eliminate cellular debris. For further purification, the supernatant was ultracentrifuged at 120 000×g for 90 min twice. The procedures of centrifugation were performed at 4 °C. The exosome pellet was resuspended in 50 µL DPBS, which was also filtered through a 0.22 µm filter to remove other large EVs.^[^ ^48^, ^49^ ^]^ Then, the morphology of the exosome was observed by transmission electron microscope (TEM, HT‐7700, Hitachi, Japan). The diameter of exosomes was detected via Nanosight tracking analysis. The expressions of exosome‐specific surface markers, including CD81 and Alix, were measured via Western blot.

### Exosome Systemic Delivery

Exosomes (5×10^8^–1×10^9^ diluted in 200 µl PBS) were transferred into the rat via tail vein injection every other day for three times as our preliminary experiments and previous studies.^[^ ^15^, ^50^, ^51^ ^]^ Plasma exosomes were separately purified from SR and AF patient blood samples, and all obtained exosomes from the same group were merged. Then, exosomes were transferred into rats via tail vein injection. ACFs‐derived exosomes were extracted from the cultured media and then injected into rats. For exosome inhibition, rats were intraperitoneally injected with GW4869 (2 mg/kg/day) for 3 days before the exosome‐transferred experiment.

The miR‐224‐5p mimics (RiboBio Co., Guangzhou, China), miR‐224‐5p inhibitors (RiboBio Co., Guangzhou, China), and their corresponding negative controls (NC) were mixed with TransMessenger Transfection Reagent (Qiagen, 301525), administered to cells for 6 h per the manufacturer's protocol. After that, the medium was changed to complete culture media without antibiotics. 24 h after miRNA mimics transfection, the cells were challenged with Ang II treatment at a dose of 1 µm for another 24 h (Sigma‐Aldrich, St. Louis, MO, USA). 24 h after cells were transfected with miRNA inhibitors, the cells were treated with the same volume of culture medium with exosome‐free FBS for another 24 h, and then were challenged with Ang II treatment.

## Conflict of Interest

The authors have no conflict of interests to declare.

## Author Contributions

Y.Y. and Y.L. designed the study. Y.Y., Z.P., and Z.Z. performed the data analysis and wrote the manuscript. X.Z. and X.H. performed the electrophysiological experiments. Y.C. and X.J. performed the molecular experiments. Y.Z. and X.B. performed the genetic mouse model. L.S., D.L., W.M., and J.S. performed patch clamp and analysis. Y.Y., X.Z., X.H., and Y.C. contributed equally to this work.

## Supporting information



Supporting Information

## Data Availability

The data that support the findings of this study are available on request from the corresponding author. The data are not publicly available due to privacy or ethical restrictions.
