# Supplementary material for: Atrial Fibroblasts‐Derived Extracellular Vesicles Exacerbate Atrial Arrhythmogenesis
Source: Adv Sci (Weinh). 2025 Jul 3;12(37):e07627. doi: 10.1002/advs.202507627 (PMC12499491; doi:10.1002/advs.202507627)
Supplement: Supplementary file 1 — Supporting Information [file ADVS-12-e07627-s001.docx]

**Supplementary Materials**

**Atrial fibroblasts-derived extracellular vesicles exacerbate atrial arrhythmogenesis**

**Running title: Atrial fibroblasts-derived extracellular vesicles promote atrial fibrillation**

Yue Yuan^1*^, Xinbo Zhao^1*^, Xuejie Han^1*^, Yukai Cao^1*^, Xuexin Jin^1^, Ling Shi^2^, Xin Bi^1^, Desheng Li^2^, Yun Zhang^1^, Wenbo Ma^2^, Jiahui Song^2^, Zhenwei Pan^2,3†^, Zhiren Zhang^1,3,7†^, Yue Li^1,4,5,6,7†^

^1^Department of Cardiology, the First Affiliated Hospital, Harbin Medical University, Harbin 150001, China

^2^Department of Pharmacology, Harbin Medical University, Harbin 150086, China
^3^Department of Pharmacology, Key Laboratory of Cardiovascular Research, Ministry of Education, College of Pharmacy, Harbin Medical University, Harbin 150086, China

^4^NHC Key Laboratory of Cell Transplantation, Harbin Medical University, Heilongjiang 150001, China

^5^Key Laboratory of Hepatosplenic Surgery, Harbin Medical University, Ministry of Education, Harbin 150001, China

^6^Key Laboratory of Cardiac Diseases and Heart Failure, Harbin Medical University, Harbin 150001, China

^7^Heilongjiang Key Laboratory for Metabolic Disorder & Cancer Related Cardiovascular Diseases, Harbin 150081, China

**Methods**

**Human primary atrial cardiac fibroblasts**

Human primary cardiac fibroblasts-atrial (HCF-aa, Catalog #6320) were purchased from ScienCell Research Laboratories. HCF-aa are isolated from the human atrium. HCF-aa from ScienCell are cryopreserved at passage one and delivered to our lab for further experiments. The cells are guaranteed to further expand less than 8 cell passages as the provided protocols. Fibroblast Medium-2 (FM-2, Cat. #2331) with Fibroblast Growth Supplement-2 (FGS-2, Cat. #2382) and fetal bovine serum was used to provide a nutritional environment that supports the growth of normal human atrial cardiac fibroblasts (ACFs). The cells are cultured in a 37°C, 5% CO_2_ incubator. Change the medium every three days, until the culture is approximately 70% confluent. Once the culture is approximately 70% confluency, change medium every other day until the culture is approximately 90% confluent. Then use 0.05% trypsin/EDTA solution (T/E, Cat. #0183) and T/E neutralization solution (TNS, Cat. #0113) to harvest cells. Centrifuge the tube at 1000 rpm for 5 minutes at room temperature. Gently resuspend cells and plate cells in a new poly-L-lysine-coated culture vessel.

**Primary cardiac fibroblasts and atrial myocytes**

Primary adult atrial myocytes (ACMs) and fibroblasts (ACFs) were isolated from the atria of (8-10)-weeks-old SD rat. Briefly, adult rat atria were minced and digested with Liberase TH (Roche, Basel, Switzerland) for 30 minutes and neutralized with KHB buffer. After being filtered through a 40-μm nylon cell strainer (BD Falcon, Franklin Lakes, NJ), the collected cells were centrifuged at 400g for 5 minutes (4°C) and resuspended with fresh culture medium (DMEM/F12 with 10% fetal bovine serum) at 37°C and 5% CO_2_ for 2-4 hours to separate aCFs from other cells via differential plating. The cells were then cultured separately in DMEM/F12 containing 10% FBS and 1% penicillin and streptomycin. ACFs were used in experiments after the cells formed a confluent monolayer and contracted in synchrony after 48 hours of incubation. Both rat/mouse atrial myocytes were isolated via Langendorff perfusion and preserved in KB buffer to perform further experiments.

Primary human atrial fibroblasts (hACFs) were isolated from the right atria of patients. Human atria were minced and digested with Liberase TH (Roche, Basel, Switzerland) and filtered through a 40-μm nylon cell strainer, the collected cells were incubated in fresh culture medium (DMEM/F12 with 10% fetal bovine serum) at 37°C and 5% CO_2_ for 2-4 hours to separate CFs from other cells via differential plating. hACFs were used in experiments after getting a confluent monolayer and contracted in synchrony after 48 hours of incubation.

**Exosome labeling**

For exosome uptake experiments, exosomes were labeled with PKH26 Fluorescent Cell Linker Kit (Sigma-Aldrich, USA) according to the protocol. Resuspended exosomes or equal volumes of PBS (control; 100 μl) were added to 150 μl of Diluent C (Sigma-Aldrich, USA). In parallel, 2 μl of PKH26 dye was added to 500 μl of Diluent C and incubated with the exosome-Diluent C solution or the PBS-Diluent C solution for 5 minutes at room temperature. To bind excess dye, 500 μl of 1% BSA in PBS was added. The labeled exosomes and PBS control were purified using differential centrifugation to remove the excess dye and resuspended in 100 μl of PBS for uptake experiments. Cardiomyocytes were stained for α-actinin. The uptake of labeled exosomes by cardiomyocytes was visualized by confocal microscopy (Zeiss, Germany).

**Transfection of miRNA agomiR and antagomir into rats**

Mature rno-miR-224-5p agomir (RiboBio Co., Guangzhou, China), mature miR-224-5p antagomir (RiboBio Co., Guangzhou, China) and their respective negative control (RiboBio Co., Guangzhou, China) were used in animal experiments following the manufacturer’s protocol. Rats were injected with 50 nmol mature miR-224-5p agomir/antagomir or a negative control in 200 μl of saline buffer every other day for 3 times as previous paper ^52^. The solution was delivered via tail vein injection into the circulating blood.

**Adeno associated virus-9 (AAV9) system**

Adeno-associated virus 9 (AAV9) was used to express negative control or miR-224-5p in an atrial myocyte-specific manner. Mice were injected with a single dose (1*10^12^GC/time) of AAV9-hTCF21-NC or AAV9-hTCF21 encoding miR-224-5p sponge virus via the retro-orbit route^53^.

**Programmed Intracardiac Stimulation**

Atrial fibrillation (AF) was induced with essentially the same protocol as described previously in detail^54^. Briefly, rats underwent open-chest electrophysiological programmed stimulation under 1% sodium pentobarbital (30 mg/kg) anesthesia. The 1.9-F octapolar catheter (Transonic Systems Inc, New York, USA) was placed on the right atrium for programmed stimulation. To assess AF inducibility, 50-Hz burst pacing was applied for 3-second with 12 bursts separated by a 2-second interval. AF was defined as >1 second of irregular atrial electrograms (>800 beats/min) with irregular ventricular response. AF duration was defined as the mean duration of all AF episodes within 60 second s in each rat.

Programmed intracardiac stimulation in mouse was performed to assess AF inducibility as described ^55^. A 1.1-F octapolar EP catheter (FTS-1113A-1018, Transonic Scisense lnc., Ontario, Canada) was inserted into the right atrium to perform inducible AF testing. AF was induced by an overdrive pacing protocol, starting with 2 seconds burst pacing at a cycle length of 40 ms and decreasing in each successive burst by a 2-ms decrement to a cycle length of 10 ms. AF was defined as lasting ≥1 second of irregular atrial electrograms. To determine whether AF inducibility was reproducible, mice were subjected to the same protocols 3 times, and when the mice that exhibited 2 or 3 times the AF evoked by burst pacing were considered AF-positive. The experimenter was blinded to genotype or viral gene-transfer status of mice.

**Echocardiography**

Echocardiography was performed to evaluate cardiac function. Ultrasound gel was applied to the chest of isoflurane-anesthetized animals, and echocardiographic monitoring was tested by Vevo 2100 (FUJIFILMS VisualSonics, Canada). The images were collected in the parasternal short-axis view.

**Doppler**

Doppler was used to measure the mitral flow. Mice were imaged under anesthesia (1.5% isoflurane in 100%, 1.0-1.5 L/min) using a 10 Hz linear probe, and the mitral Doppler inflow spectrum was recorded from the parasternal long axis^53^.

**Surface ECG recording**

Surface ECG was conducted to record spontaneous AF incidence and duration of mice under anesthesia (1.5% isoflurane in 100%, 1.0-1.5 L/min). Body temperature was controlled between 36.0°C and 36.5°C for 20-30 min recordings. Data were recorded continuously and analyzed by iWorx software.

**miRNA and mRNA Sequence and bioinformatic analysis**

The cell and exosome samples were lysed, and the microRNA content was extracted by kit (Axygen, USA). Then, miRNA and mRNA sequencing were performed by a commercial service at Guangzhou RiboBio Co., Ltd. with the Illumina HiSeq 3000. Differential expression analysis of the miRNA-seq and mRNA-seq were examined according to a fold-change (FC) ≥1.5 and p value <0.05. The bioinformatic analysis was performed through Gene Ontology (GO) categories and Kyoto Encyclopedia of Genes and Genomes (KEGG) database.

**RNA Isolation and Quantitative Real Time-PCR (qRT-PCR)**

Total RNAs of exosome miRNAs were isolated by kits (Axygen, USA). Then the miRNAs were transferred into cDNAs by kit (Tyobo, No. FSQ-**101 and** FSQ-**301**). qRT-PCR was performed on Applied Bio-system. The relative levels of miRNAs and mRNAs were calculated and quantified using 2 ^−ΔΔCT^ method after normalization with U6 or GAPDH. Bulge-loop^TM^ miRNA qRT-PCR Primer Sets (one RT primer and a pair of qPCR primers for each set) specific for miR-224-5p and U6 are designed by RiboBio (Guangzhou, China). The sequences of CACNA1C and GAPDH primers:

CACAN1C Rat: Forward primer (5’-3’) GTCGGCATAAGCCTCAGAGC

CACAN1C Rat: Reverse primer (5’-3’) GCCAGGCTCAGACAGAACAT

CACAN1C Mouse: Forward primer (5’-3’) TGAGCGAGATCGATGACCCA

CACAN1C Mouse: Reverse primer (5’-3’) ACGATCAGCAAAGCCACGTA

GAPDH Rat: Forward primer (5’-3’) CCTCTGACTTCAACAGCGACCAC

GAPDH Rat: Reverse primer (5’-3’) TGGTCCAGGGGTCTTACTCC

GAPDH Mouse: Forward primer (5’-3’) TGGAGTCTACTGGCGTCTT

GAPDH Mouse: Reverse primer (5’-3’) TGTCATATTTCTCGTGGTTCA

**Luciferase Reporter Assay**

Primary rat neonatal ACMs were co-transfected with CACNA1C 3'UTR/Mut (human mutant plasmid nucleic sequence, 12473-ACCAAGG-12467; rat mutant CACNA1C nucleic sequence, 10349-ACGAAGGCA-10341) or CACNA1C 3'UTR/WT and 50nmol/L miR-224-5p mimic or negative control (RiboBio, Guangzhou, China) using Lipofectamine 3000 (Invitrogen) following the instructions. Cells were incubated for 6 hours with the transfection complex before it was replaced by fresh media. After 48 hours, the cells were harvested, and fireﬂy and renilla luciferase activities were analyzed with the Dual-Luciferase Reporter Assay System (Promega, USA) according to protocol. Luciferase activity was normalized by the Renilla/Firefly luciferase signal in HEK293T cells and rat native atrial myocytes.

**RISK Pulldown Assay**

To investigate the direct between miR-224-5p and CACNA1C mRNA, we conducted RNA-RNA pull-down assay using the biotin pull-down assay, followed by qRT-PCR analysis. In brief, miR-224-5p and a mutant miR-224-5p were synthesized in vitro and labeled with biotin. Subsequently, the biotinylated RNAs were incubated with rat cardiomyocyte extracts or rat cardiac fibroblast extracts and then captured using streptavidin-coated beads. Then qRT-PCR was performed to measure the level of CACNA1C mRNA in rat neonatal ACMs.

**Western blot**

The detailed method of Western blot was referred to our previous study ^56^. Whole protein of exosomes and cell lysate were extracted and resolved on 10% SDS-PAGE and then transferred on 0.45 μm polyvinylidene fluoride membrane. The membrane was blocked with 5% BSA for 1 hour at room temperature, then incubated with primary antibody at 4 °C overnight. The membrane was incubated with related secondary antibody for 1 hour. Expressions of protein were analysis by ChemiDoc XRS gel documentation system via ECL kit (Thermo). Primary antibody applicated in western blot as follow: Alix, Abcam (ab275377), Rabbit, 1:800; CD81, Proteintech (27855-1-AP), Rabbit, 1:1000; Cav1.2, Abcam (ab84814), Mouse, 1:500; GAPDH, CST (#5174), Rabbit, 1:1000.

**Histological staining**

Rat and mouse atrial tissues were fixed in 4% paraformaldehyde and then embedded with paraffin. The sections were continuously cut into 7 mm and stained with hematoxylin and eosin (HE) (Cat. G1120, Solarbio, Beijing, China), Masson (Cat. HT10516, Sigma, USA) and Picrosirius staining. Histopathological changes were studied through light microscopy. For immunofluorescent staining, paraffin embedded heart tissues were cut into 8 μm sections. The slides were treated with citrate buffer and stained with primary antibodies (αActinin, Abcam, ab68194; Cav1.2, Abcam, ab84814; αSMA, Abcam, ab7817; TnT, Abcam, ab8295; CD64, Thermo, MA-29706; CD31, Thermo, 48-0319-42) at 4°C overnight. The sections were then stained with an Alexa Fluor 488 or 568 conjugated secondary antibodies. Nuclei were stained by DAPI, extracellular matrix was using WGA (Thermo Fisher, W11261) ^56^.

**EdU kit**

The cellular viability was detected by an EdU assay kit (C10310−1, RiboBio, China). Atrial fibroblasts were cultured with EdU reagents (10 μmol/L) for 4 h at 37 °C. The cells were washed with PBS then fixed with 4% paraformaldehyde for 30 min and incubated with 0.5% Triton X-100 for 15 min. 100 μL of 1× Apollo reaction cocktail was exposed for 30 min and stained with Hoechst for 30 min to cells. Images were taken via fluorescent microscope (Zeiss, Germany). ImageJ software was used to analyze.

**Whole-Cell Patch Clamp**

The L-type calcium current (*I*_CaL_) and APD of isolated rat or mouse atrial cardiomyocytes were recorded using the whole-cell patch clamp technique, according to the procedures described previously with minor modifications ^57^. The pipette of patch electrodes had the tip resistance of 2.5-4 MΩ when filled with pipette solution. Whole-cell recording was performed using an amplifier (Axopatch 700B, Axon instrument, USA). Signals were filtered at 1 kHz and data were acquired by A/D conversion (Digidata 1440, Axon Instrument). Ion currents were recorded in the voltage-clamp mode. For the recording of *I*_CaL_ current, the external solution for *I*_CaL_ recording contained (in mM): 120 tetraethylammonium (TEA), 10 HEPES, 1.0 MgCl_2_, 10 CsCl, 10 glucose, and 1.8 CaCl_2_, and the pH was adjusted to 7.3 with CsOH. The pipette solution contained (in mM): 120 CsCl, 40 CsOH, 1 MgCl_2_, 11 EGTA, 5 Mg-ATP, and 10 HEPES and the pH was adjusted to 7.3 with CsOH. Individual currents were normalized to the membrane capacity to control for differences in cell size (current density, pA/pF). Action potentials were recorded in current clamp mode at a pacing rate of 1 Hz. For the recording of APD, the external solution for APD recording contained (in mM): 136 NaCl, 5.4 KCl, 1.0 MgCl_2_·6H_2_O, 1.8 CaCl_2_, 10.0 HEPES, 1.0 glucose; pH adjusted to 7.4 with NaOH. The pipette solution contained (mM): 120 K-aspartate, 30 KCl, 1 MgCl_2_, 1 CaCl_2_ and 10 EGTA, and the pH was adjusted to 7.2 with KOH.

**References**

52. Hou Z, Qin X, Hu Y, Zhang X, Li G, Wu J, Li J, Sha J, Chen J, Xia J, Wang L and Gao F. Longterm Exercise-Derived Exosomal miR-342-5p: A Novel Exerkine for Cardioprotection. *Circulation research*. 2019;124:1386-1400.

53. Yuan Y, Martsch P, Chen X, Martinez E, Li L, Song J, Poppenborg T, Bruns F, Kim JH, Kamler M, Martin JF, Abu-Taha I, Dobrev D and Li N. Atrial cardiomyocyte-restricted cleavage of gasdermin D promotes atrial arrhythmogenesis. *European heart journal*. 2025.

54. Luo Y, Zhang Y, Han X, Yuan Y, Zhou Y, Gao Y, Yu H, Zhang J, Shi Y, Duan Y, Zhao X, Yan S, Hao H, Dai C, Zhao S, Shi J, Li W, Zhang S, Xu W, Fang N, Gong Y and Li Y. Akkermansia muciniphila prevents cold-related atrial fibrillation in rats by modulation of TMAO induced cardiac pyroptosis. *EBioMedicine*. 2022;82:104087.

55. Li N and Wehrens XH. Programmed electrical stimulation in mice. *Journal of visualized experiments : JoVE*. 2010.

56. Yuan Y, Zhao J, Gong Y, Wang D, Wang X, Yun F, Liu Z, Zhang S, Li W, Zhao X, Sun L, Sheng L, Pan Z and Li Y. Autophagy exacerbates electrical remodeling in atrial fibrillation by ubiquitin-dependent degradation of L-type calcium channel. *Cell death & disease*. 2018;9:873.

57. Zhang Z, He Y, Tuteja D, Xu D, Timofeyev V, Zhang Q, Glatter KA, Xu Y, Shin HS, Low R and Chiamvimonvat N. Functional roles of Cav1.3(alpha1D) calcium channels in atria: insights gained from gene-targeted null mutant mice. *Circulation*. 2005;112:1936-44.

**Supplementary Figure**

**Supplementary Figure 1**


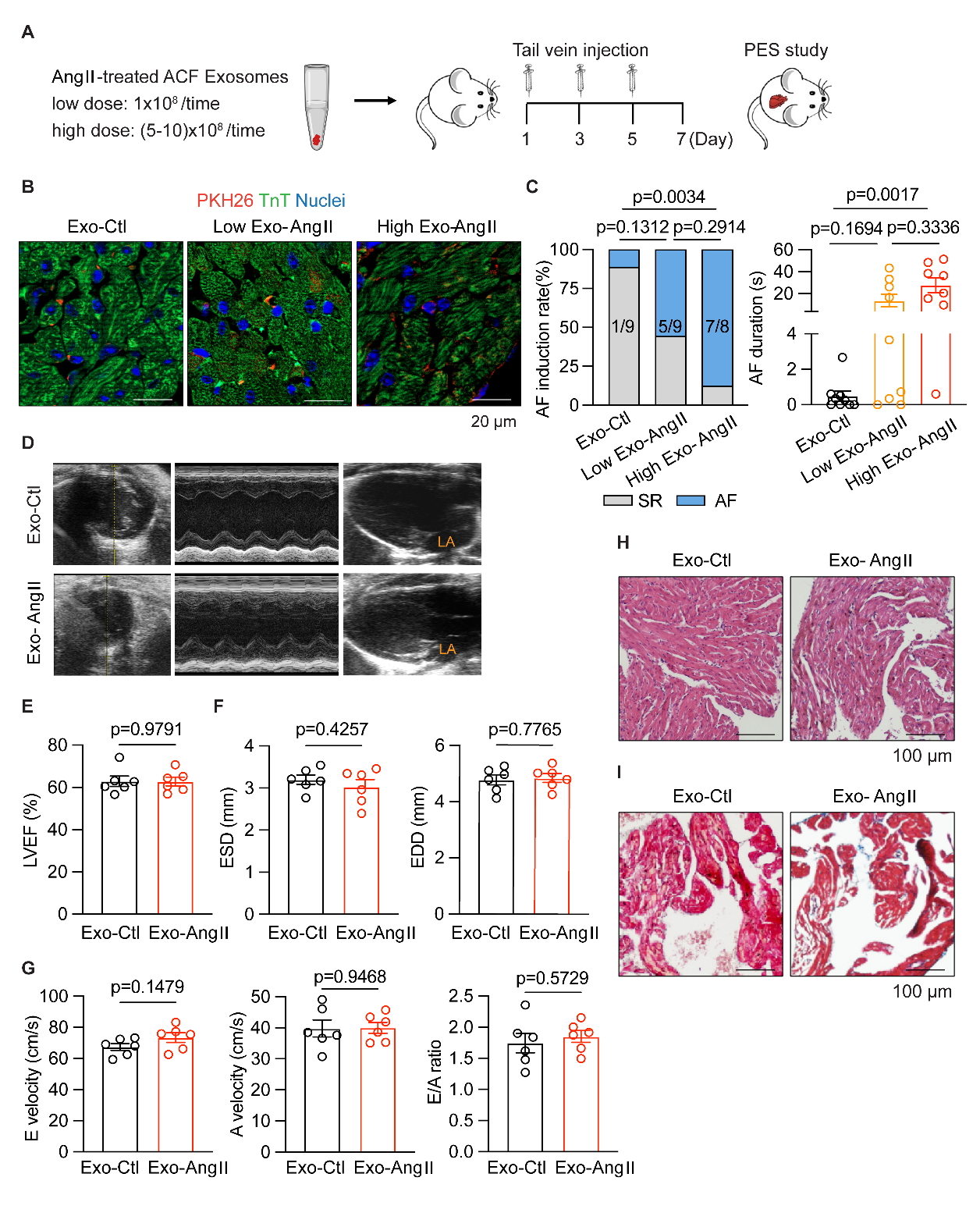


**Supplementary Figure 1. Comparable atrial and ventricular function and structure of exosomes transferred rat model.**

(**A**) Scheme for the lower and higher Ang II-treated hACFs exosome preparation, injection and AF induction test in rats. (**B**) Representative images of PKH26 (red), TnT (green) and DAPI (blue) staining in Exo-Ctl, low and high Exo-Ang II rat atria (n=3 per group). (**C**) AF incidence (n=8 or 9 per group, Exo-Ctl vs Low Exo-Ang II p=0.1312, Low Exo-Ang II vs High Exo-Ang II p=0.2914, Exo-Ctl vs High Exo-Ang II p=0.0034) and AF duration in three groups of rats (n=8 or 9 per group, Exo-Ctl vs Low Exo-Ang II p=0.1694, Low Exo-Ang II vs High Exo-Ang II p=0.3336, Exo-Ctl vs High Exo-Ang II p=0.0017). (**D**) Representative cardiac echography images of Exo-Ctl and high Exo-Ang II rats. (**E**) Quantification of LVEF (n=6 per group, p=0.9791) and left ventricular diameters (**F**) in all groups of rats (n=6 per group, ESD p=0.4257, EDD p=0.7765). (**G**) Quantification of E (n=6 per group, p=0.1479) and A velocity of mitral valve in Exo-Ctl and high Exo-Ang II rats (n=6 per group, p=0.9468). (**H**) Representative HE images of atria from Exo-Ctl and Exo-Ang II rats (n=3 per group). Scale bar: 100 μm. (**F**) Representative Masson images of atria from Exo-Ctl and Exo-Ang II rats (n=3 per group). Scale bar: 100 μm. Exo-Ctl, control hACFs-derived exosomes; Exo-Ang II, angiotensin II treated hACFs-derived exosomes; LA, left atria; LVEF, left ventricular ejection fraction; ESD, end-systolic diameter; EDD, end-diastolic diameter. The bar graph data are mean±SEM with individual values. p values were determined with Fisher’s exact test in **C**, and Mann-Whitney test in **C**. p-values are determined with two-tailed unpaired Student’s *t*-test in **E-G**. p<0.05 was determined as significance.

**Supplementary Figure 2**


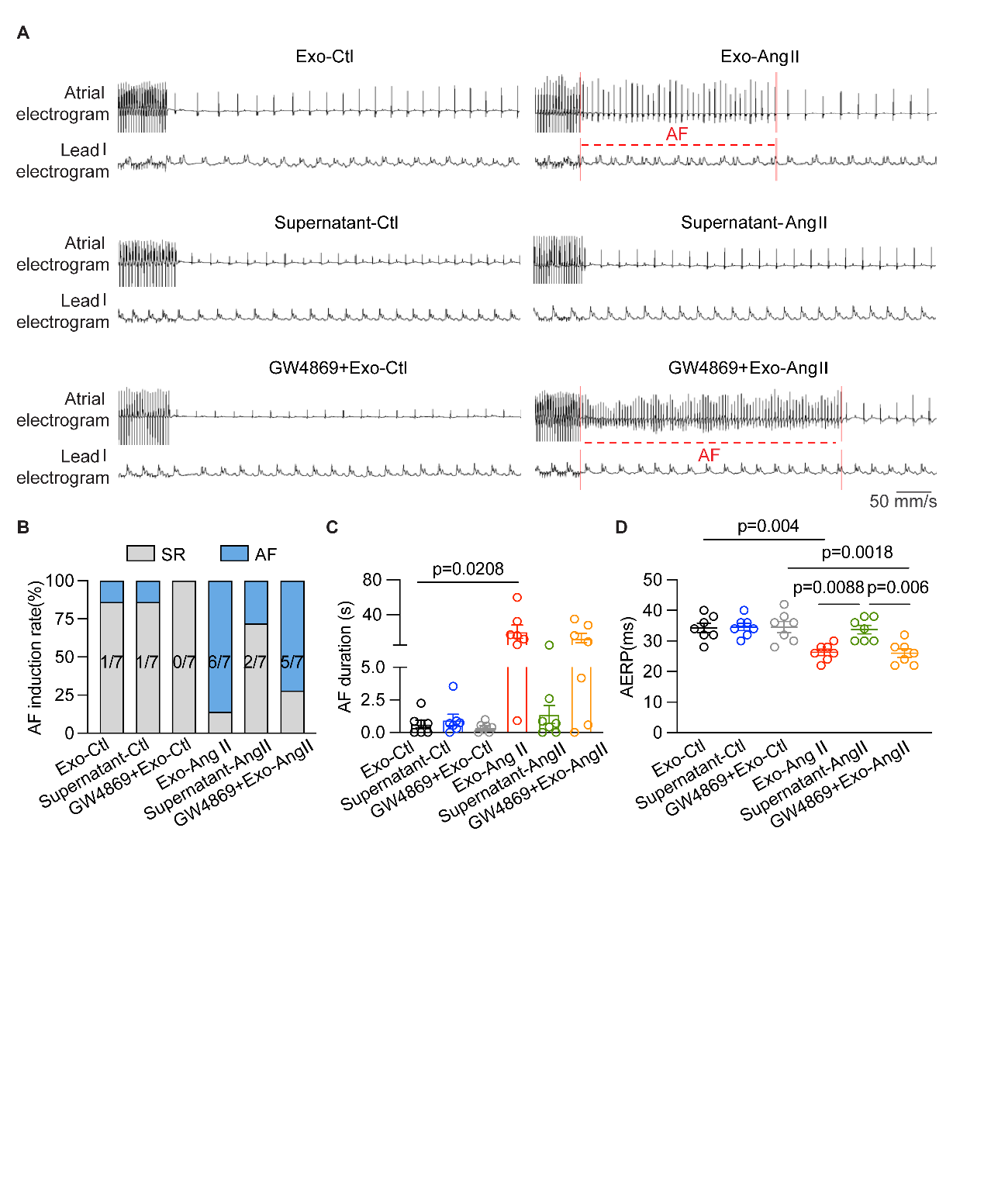


**Supplementary Figure 2. AF incidence of rat transferred with ACFs-derived supernatant.**

(**A**) Representative traces of simultaneous recordings of surface ECG (lead I) and intracardiac electrograms in rat model after programmed intracardiac stimulation (red line), and (**B**) incidence rate of AF (n=7 per group). (**C**) Quantification of AF duration in different groups of rats (n=7 per group, Exo-Ctl vs Exo-Ang II, p=0.0208). (**D**) Quantification of AERP in different group of rats (n=7 per group Exo-Ctl vs Exo-Ang II, p=0.004). The bar graph data are mean±SEM with individual values. p values were determined with two-tailed Fisher’s exact test in **B**, and Mann-Whitney test in **C**. p-values were determined with one-way ANOVA and Turkey’s multiple comparisons test in **D**.

**Supplementary Figure 3**


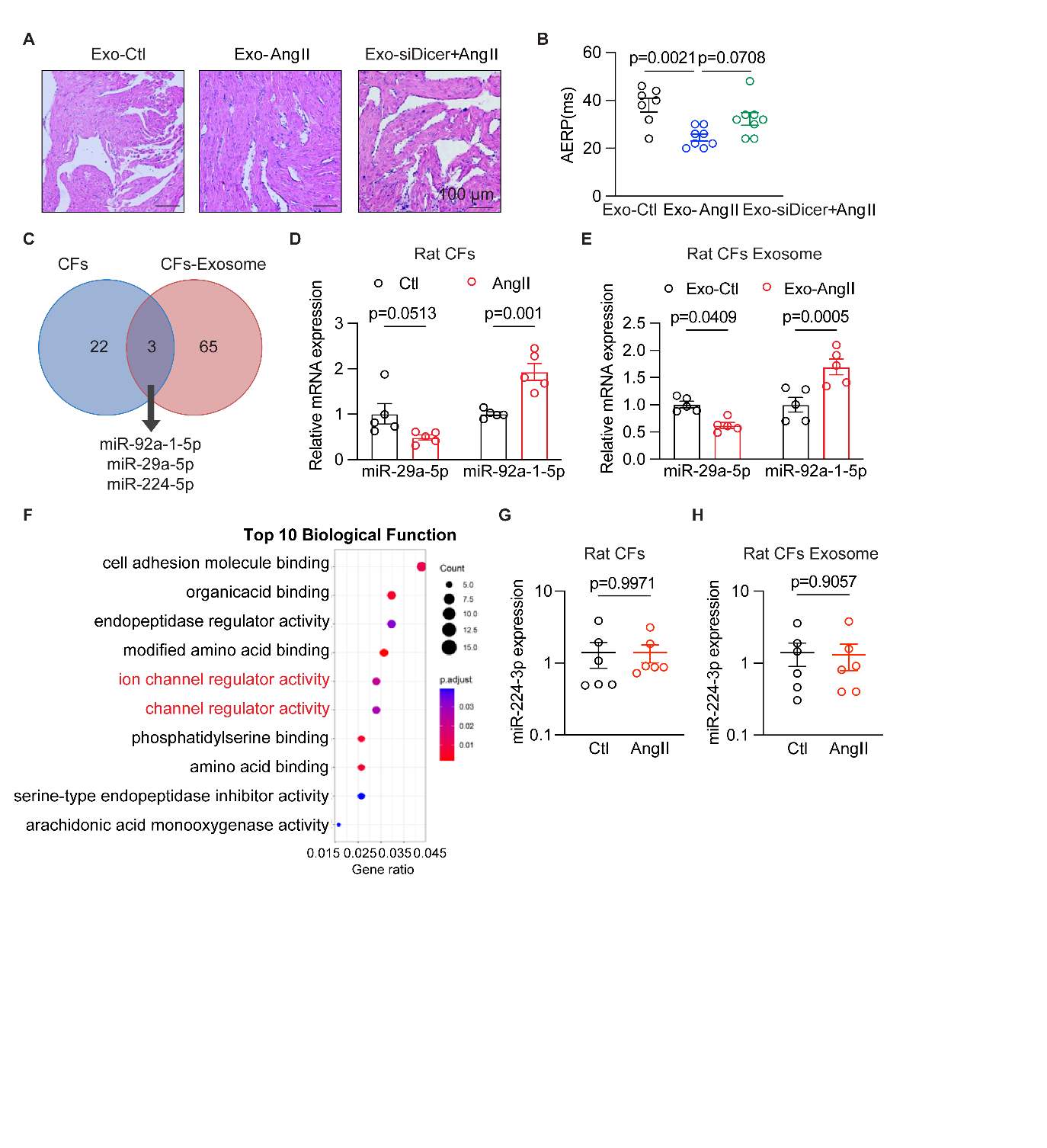


**Supplementary Figure 3. The expressions and biological function of differential miRNAs in ACFs-derived exosome.**

(**A**) Representative HE images of atria from Exo-Ctl, Exo-Ang II and Exo-siDicer+Ang II rats (n=3 per group). Scale bar: 100 μm. (**B**) Quantification of AERP in Exo-Ctl, Exo-Ang II and Exo-siDicer+Ang II rats (n=7 or 8 per group, Exo-Ctl vs Exo-Ang II p=0.0021, Exo-Ang II vs Exo-siDicer + Ang II p=0.0708). (**C**) miRNAs expressed in both primary adult rat ACFs and ACFs-derived exosomes. (**D**) miR-29a-5p expression in Ctl and Ang II-treated ACFs (n=5 per group, p=0.0513) and miR-92a-1-5p expression tested by qRT-PCR (n=5 per group, p=0.001). (**E**) miR-92a-1-5p (n=5 per group, p=0.0409) and miR-92a-1-5p (n=5 per group, p=0.0005) expression in Ctl and Ang II-treated ACFs secreted exosomes tested by qRT-PCR. (**F**) GO analysis of differential ACFs-derived exosome miRNAs. (**G** and **H**) miR-224-3p expressions in Ctl and Ang II-treated ACFs (n=6 per group, p=0.9971) and their secreted exosomes tested by qRT-PCR (n=6 per group, p=0.9057). Exo-Ctl, control ACFs-derived exosomes; Exo-Ang II, Ang II induced ACFs-derived exosomes and Exo-siDicer + Ang II, knocking down siRNA and Ang II induced ACFs-derived exosomes. The bar graph data are mean±SEM with individual values. p values were determined with two-tailed unpaired Student’s *t*-test in **D**, **E**, **G** and **H**. p-values were determined with one-way ANOVA and Turkey’s multiple comparisons test in **B**.

**Supplementary Figure 4**


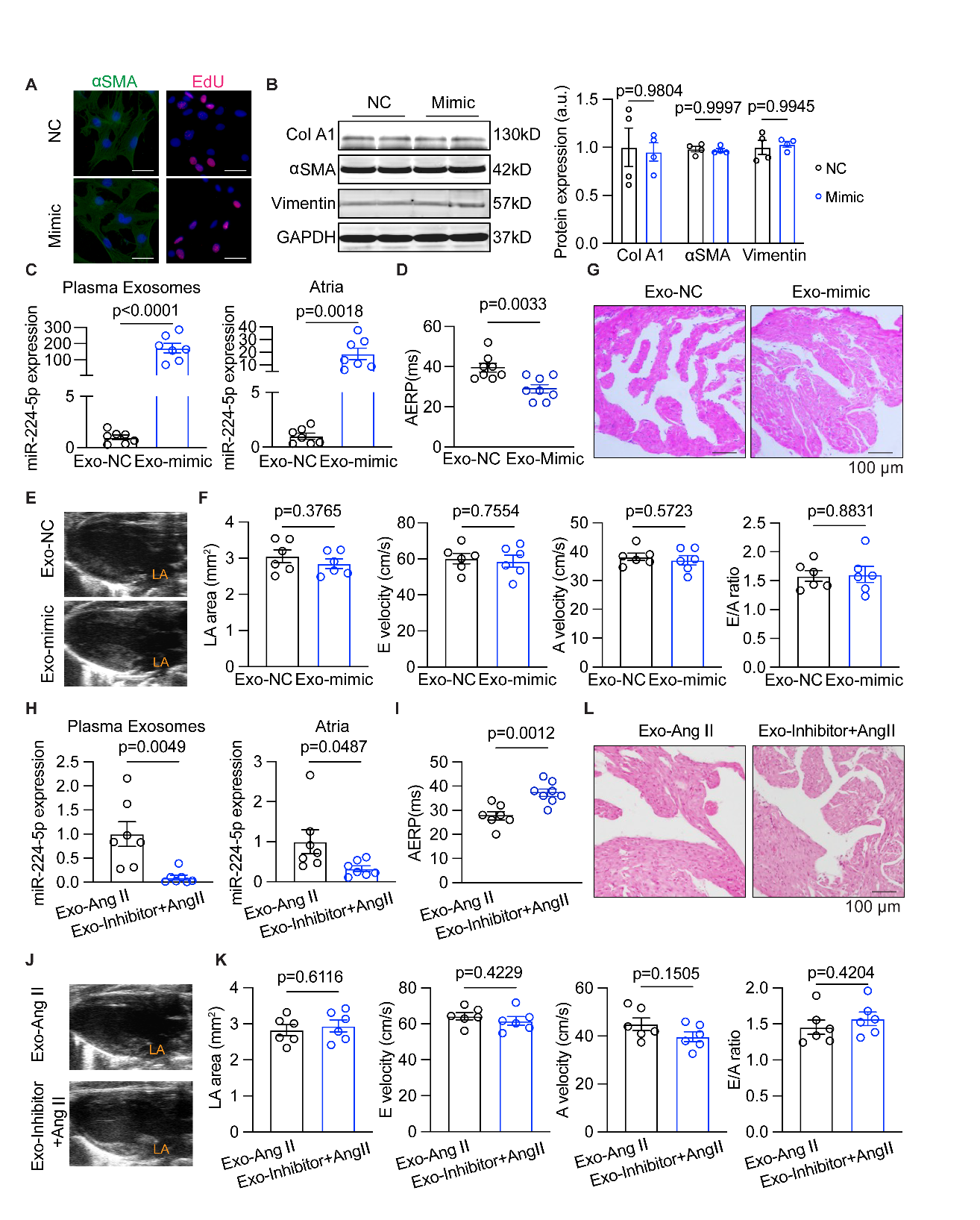


**Supplementary Figure 4. Atrial function and structure of miR-224-5p mimics or inhibitor treated-ACFs exosomes transferred rats.**

(**A**) αSMA and EdU staining images of negative control and mimic miR-224-5p transfected rat ACFs (n=4 per group). Scale bar: 20 μm. (**B**) Western blot of ColA1, αSMA and Vimentin protein expressions in NC and Mimic transfected rat ACFs (n=4 per group, Col A1 p=0.9804, αSMA p=0.9997, Vimentin p=0.9945). (**C**) miR-224-5p expressions in plasma exosomes (n=7 per group, p<0.0001) and atrial tissues in Exo-NC and Exo-mimic transferred rats (n=7 per group, p=0.0018). (**D**) Quantification of AERP in Exo-NC and Exo-Mimic rat (n=8 per group, p=0.0033). (**E** and **F**) Representative echocardiography images of left atria (n=6 per group, p=0.3765) and, quantification of E (n=6 per group, p=0.7554) and A velocity (n=6 per group, p=0.5723) of mitral valve in Exo-NC and Exo-Mimic rats. (**G**) Representative of HE images of atria from Exo-NC and Exo-mimic rats (n=3 per group). Scale bar: 100 μm. (**H**) miR-224-5p expressions in plasma exosomes (n=7 per group, p=0.0049) and atrial tissues in Exo-Ang II and Exo-Inhibitor+Ang II transferred rats (n=7 per group, p=0.0487). (**I**) Quantification of AERP in Exo-Ang II and Exo-Inhibitor+Ang II rats (n=7 or 8 per group, p=0.0012). (**J** and **K**) Representative echocardiography images of left atria (n=6 per group, p=0.6116) and, quantification of E (n=6 per group, p=0.4229) and A velocity (n=6 per group, p=0.1505) of mitral valve in Exo-Ang II and Exo-Inhibitor+Ang II rats (n=6 per group). (**L**) Representative of HE images of atrial tissues from Exo-Ang II and Exo-Inhibitor+Ang II rats (n=3 per group). Scale bar: 100 μm. Exo-NC, exosomes of CFs transfection with negative control; Exo-mimic-miR-224-5p, exosomes of CFs transfection with mimics of miR-224-5p. Exo- Ang II, exosomes of Ang II treated ACFs; Exo-Inhibitor+Ang II, exosomes of transfection with miR-224-5p inhibitor and Ang II treated ACFs; NC, negative control; mimic, mimic of miR-224-5p. The bar graph data are mean±SEM with individual values. p values were determined with two-tailed unpaired Student’s *t*-test in **B, C, D, F, H, I** and **K**.

**Supplementary Figure 5**


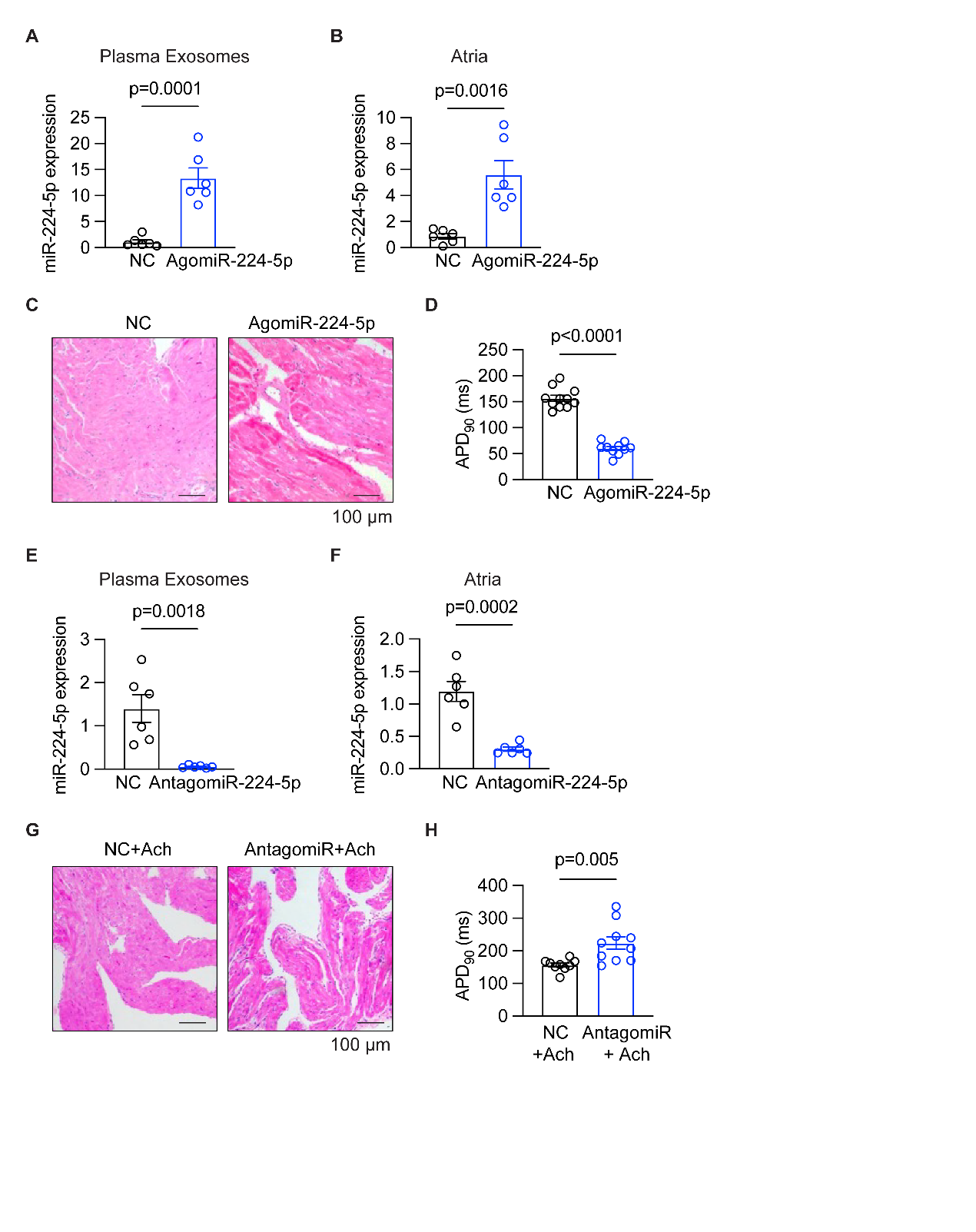


**Supplementary Figure 5. Atrial structure and APD_90_ of miR-224-5p agomiR and antagomiR treated rats.**

(**A** and **B**) miR-224-5p expressions in plasma exosomes (n=6 per group, p=0.0001) and atrial tissues in NC and AgomiR-224-5p rats (n=6 per group, p=0.0016). (**C**) Representative of HE images of atrial tissues from NC and AgomiR-224-5p rats (n=3 per group). Scale bar: 100 μm. (**D**) The quantification of APD_90_ in isolated ACMs (n=10 or 11 per group, p<0.0001). (**E** and **F**) miR-224-5p expressions in plasma exosomes (n=6 per group, p=0.0018) and atrial tissues in NC and AntagomiR-224-5p rats (n=6 per group, p=0.0002). (**G**) Representative of HE images of atrial tissues from NC and AntagomiR-224-5p rats (n=3 per group). Scale bar: 100 μm. (**H**) The quantification of APD_90_ in isolated atrial myocytes (n=9 or 10 per group, p=0.005). NC, negative control; AgomiR-224-5p, agomiR of miR-224-5p; AntagomiR-224-5p, antagomiR of miR-224-5p. The bar graph data are mean±SEM with individual values. p values were determined with two-tailed unpaired Student’s *t*-test in **A, B, D, E, F** and **H**.

**Supplementary Figure 6**


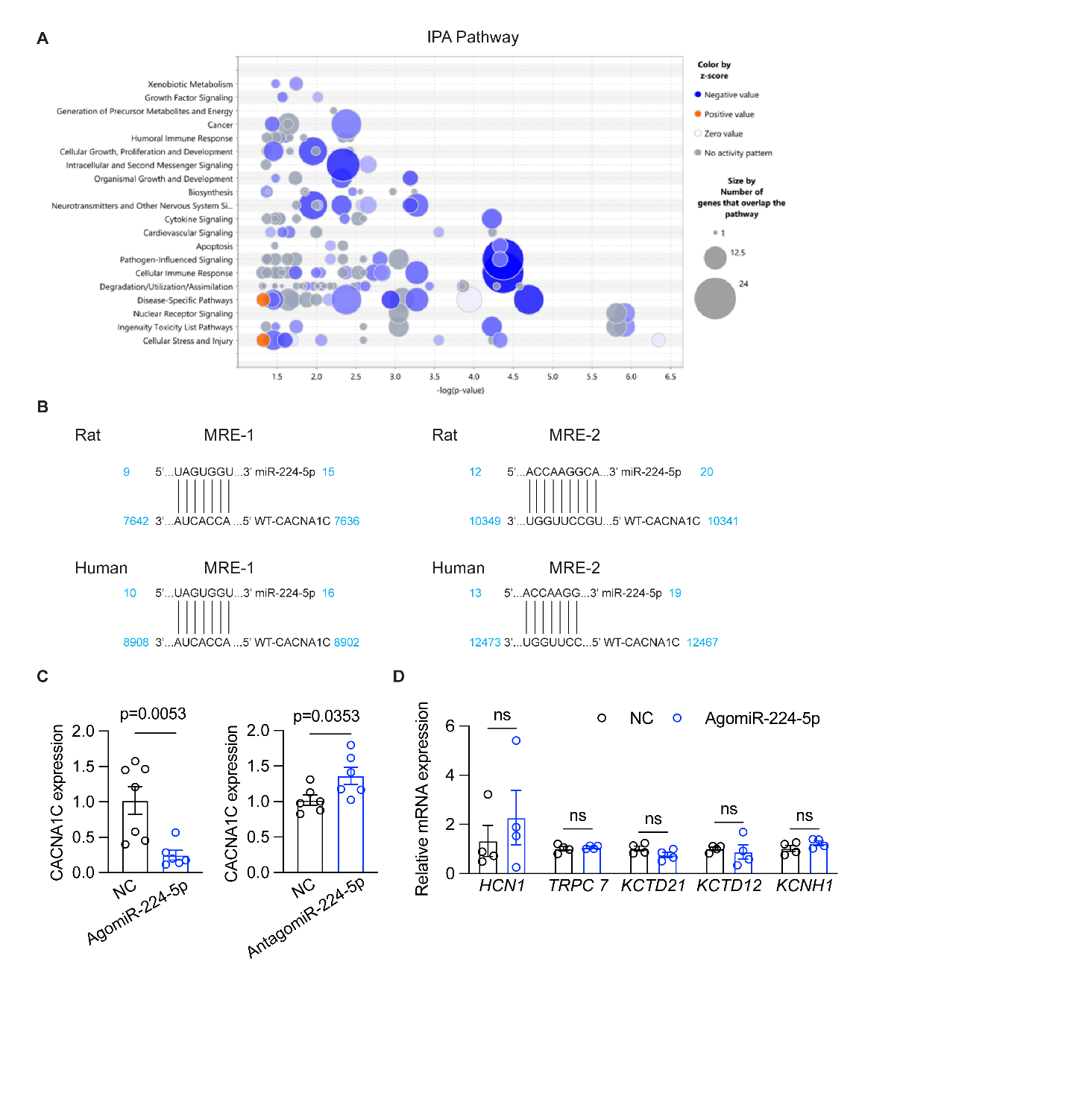


**Supplementary Figure 6. miR-224-5p inhibits the transcription of CACNA1C not potassium channel.**

(**A**) IPA analysis of differential downstream genes in atria of NC and AntagomiR-224-5p rats (n=3 per group). (**B**) Predicted MRE between miR-224-5p and CACNA1C in human and rat. (**C**) (Left) miR-224-5p expression in atrial tissues in NC and AgomiR-224-5p rats (n=6 or 7 per group, p=0.0053). (Right) miR-224-5p expression in atrial tissues in NC and AntagomiR-224-5p rats (n=6 per group, p=0.0353). (**D**) Potassium channel related gene expressions in NC and AgomiR-224-5p administered rat atria (n=4 per group, ns. p>0.05). NC, negative control; AgomiR-224-5p, agomiR of miR-224-5p; AntagomiR-224-5p, antagomiR of miR-224-5p. The bar graph data are mean±SEM with individual values. p values were determined with two-tailed unpaired Student’s *t*-test in **C**. p-values were determined with two-tailed unpaired Student’s *t*-test in **D**.

**Supplementary Figure 7**


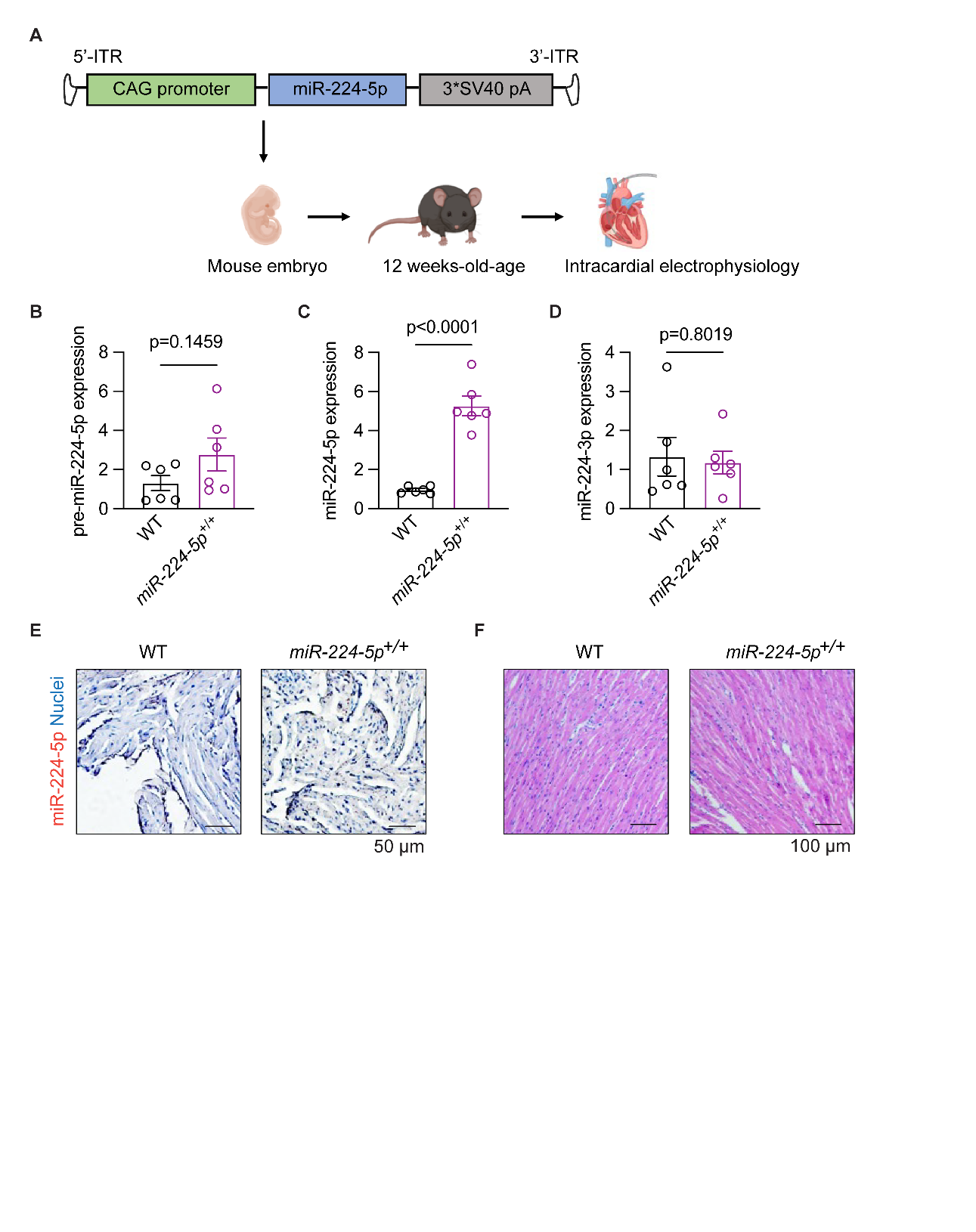


**Supplementary Figure 7. Global *miR-224-5p^+/+^* mouse model and atrial miR-224-5p expressions.**

(**A**) Construction of miR-224-5p conventional overexpression mice. Intracardiac programmed stimulation was employed to detect AF incidence of WT and global *miR-224-5p^+/+^* mice at 12 weeks-old-age. (**B-D**) PremiR-224-5p (n=6 per group, p=0.1459), miR-224-5p (n=6 per group, p<0.0001) and miR-224-3p (n=6 per group, p=0.8019) expressions in the atria of WT and global *miR-224-5p^+/+^* mice. (**E**) Mouse atria stained with miR-224-5p-probe (red) and Nuclei by Hematoxylin (Blue) respectively (n=3 per group). Scale bar: 50μm. (**F**) Representative of HE images of atrial tissues in WT and *miR-224-5p^+/+^* mice (n=3 per group). Scale bar: 100μm. WT, wildtype; *miR-224-5p^+/+^*, global miR-224-5p knock-in mice. The bar graph data are mean±SEM with individual values. p values were determined with unpaired Student’s *t*-test in **B, C** and **D**.

**Supplementary Figure 8**


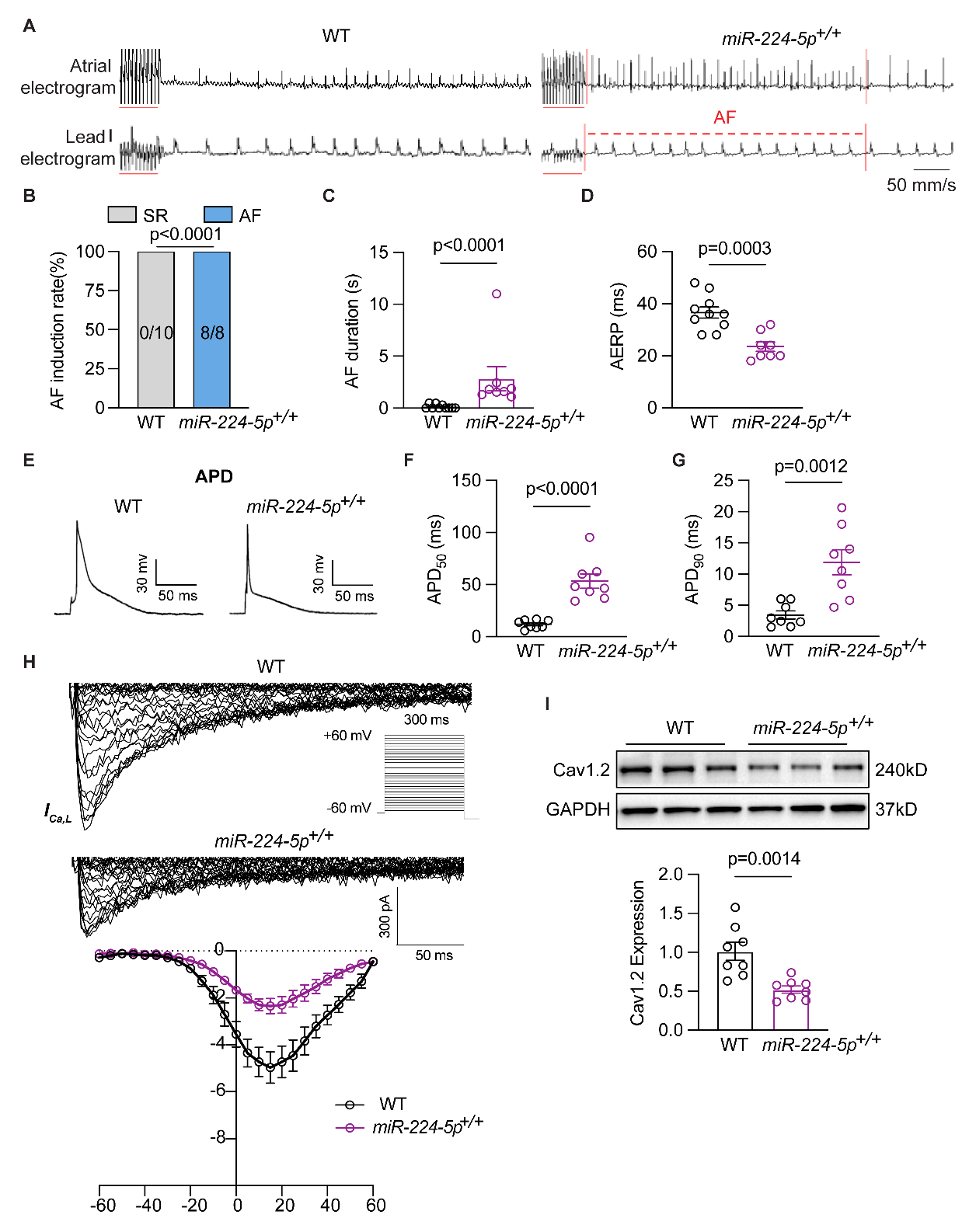


**Supplementary Figure 8**. **Global** **miR-224-5p knock-in mice increases AF susceptibility.**

(**A**-**C**) Representative traces of simultaneous recordings of surface ECG (lead I) and intracardiac electrograms in WT and *miR-224-5p^+/+^* mice after programmed intracardiac stimulation (red line), and incidence rate (n=8 or 10 per group, p<0.0001) and duration of AF (n=8 or 10 per group, p<0.0001). (**D**) Quantification of AERP in WT and *miR-224-5p^+/+^* mice (n=8 or 10 per group, p=0.0003). (**E**-**G**) Representative recordings of APD and quantification of APD_50_ (n=8 per group, p<0.0001) and APD_90_ in atrial myocytes isolated from WT and *miR-224-5p^+/+^* mice (n=8 per group, p=0.0012). (**H**) Representative recordings and I-V curve of *I*_Ca,L_ in isolated atrial myocytes of WT and *miR-224-5p^+/+^* mice (n=9 per group). (**I**) Western blot testing Cav1.2 protein level in the atria of WT and *miR-224-5p^+/+^* mice (n=8 per group, p=0.0014). The bar graph data are mean±SEM with individual values. p values were determined with wo-tailed Fisher’s exact test in **B**, and two-tailed Mann-Whitney test in **C**. p values were determined with two-tailed unpaired Student’s *t*-test in **D**, **F**, **G**, **H** and **I**.

**Supplementary Figure 9**


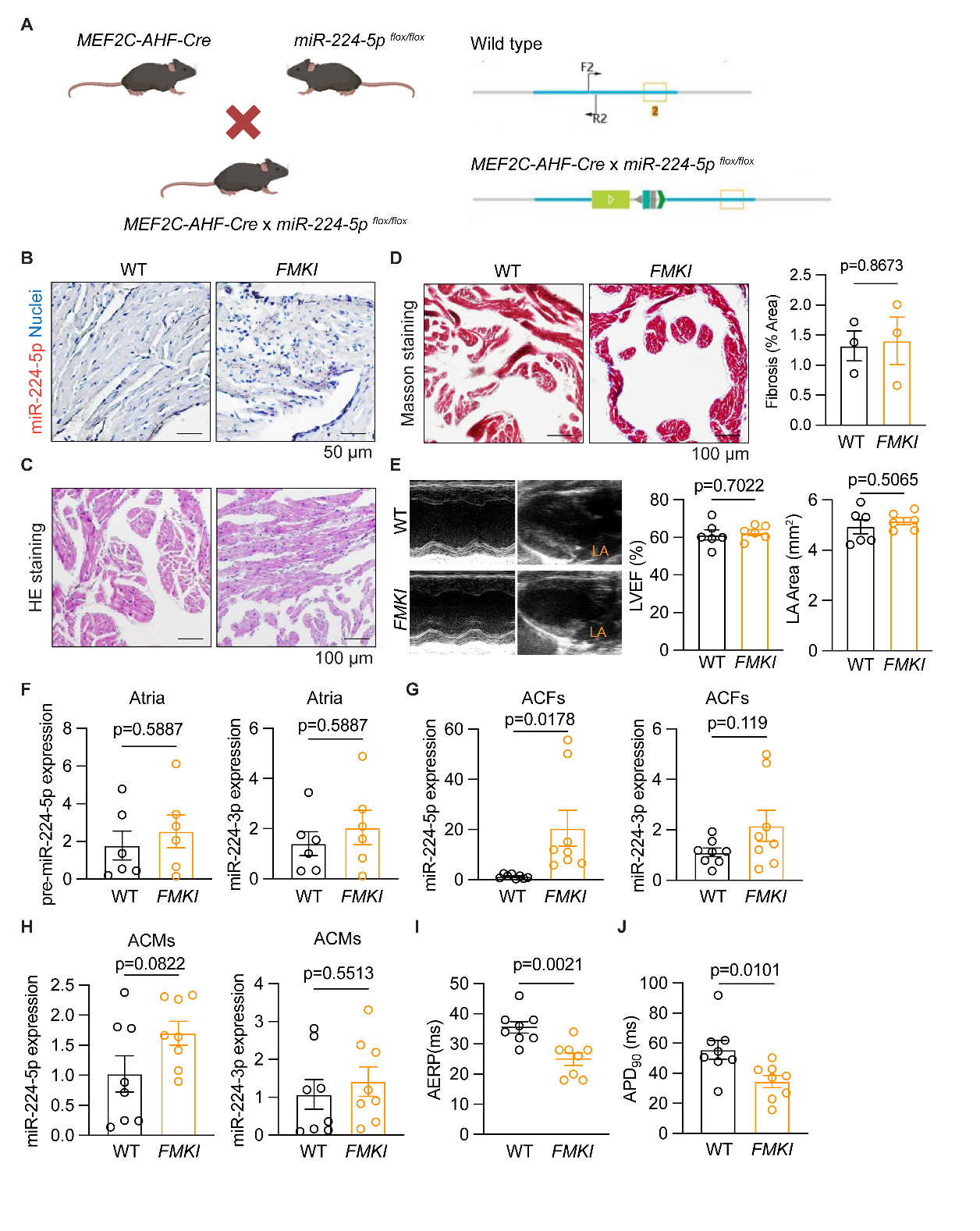


**Supplementary Figure 9**. **Fibroblast-specific miR-224-5p knock-in mice induced electrical remodeling.**

(**A**) Construction of fibroblast-specific miR-224-5p knock-in mice. (**B**) Atrial samples labeled with miR-224-5p-probe (red) and Nuclei probe (Blue) respectively (n=3 per group). Scale bar: 50μm. (**C**) Representative HE images of atria in WT and *FMKI* mice (n=3 per group). Scale bar: 50μm. (**D**) Representative Masson staining images of atria in WT and *FMKI* mice (n=3 per group, p=0.8673). Scale bar: 50μm. (**E**) Representative cardiac echography images and quantification of LVEF (n=6 per group, p=0.7022) and LA area in WT and *FMKI* mice (n=6 per group, p=0.5065). (**F**) PremiR-224-5p (n=6 per group, p=0.5887) and miR-224-3p expressions in atria of WT and *FMKI* mice (n=6 per group, p=0.5887). (**G**) miR-224-5p (n=8 per group, p=0.0178) and miR-224-3p expressions in ACFs isolated from WT and *FMKI* mice (n=8 per group, p=0.119). (**H**) miR-224-5p (n=8 per group, p=0.0822) and miR-224-3p expression in isolated ACMs from WT and *FMKI* mice (n=8 per group, p=0.5513). (**I** and **J**) Quantification of AERP (n=8 per group, p=0.0021) and APD_90_ in WT and *FMKI* mice (n=8 per group, p=0.0101). WT, wildtype; *FMKI*, fibroblast-specific miR-224-5p knock-in mice. The bar graph data are mean±SEM with individual values. p values were determined with two-tailed unpaired Student’s *t*-test in **D**, **E**, **F**, **G**, **H**, **I** and **J**.

**Supplementary Figure 10**


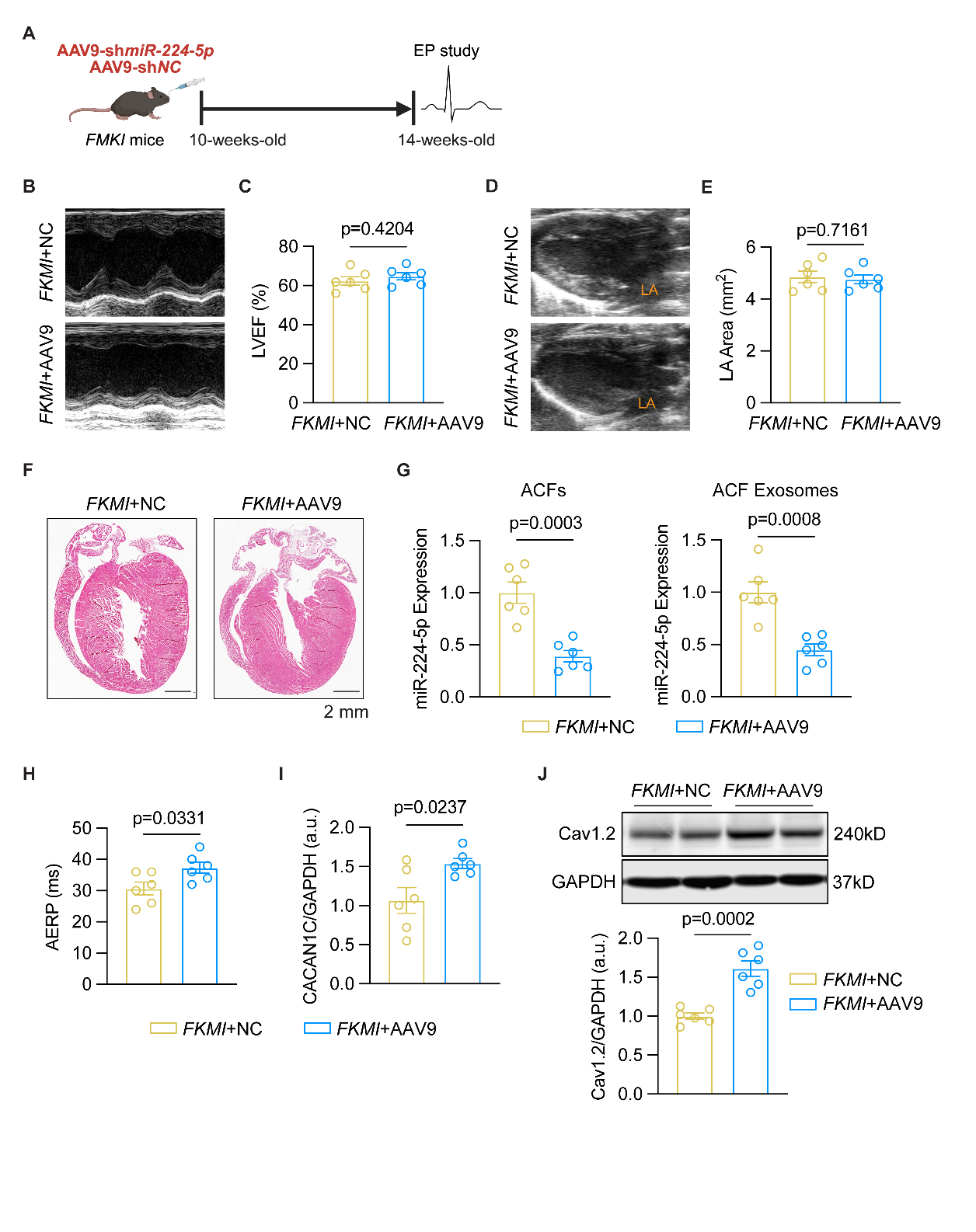


**Supplementary Figure 10**. **Heart function and structure in *FMKI* mice treated with AAV9-sh*miR-224-5p*.**

(**A**) Study design for *FKMI* mice injected with NC or AAV9-sh*miR-224-5p*. (**B**) Representative cardiac echography images and (**C**) quantification of LVEF in *FMKI*+NC and *FMKI*+AAV9 mice (n=6 per group, p=0.4204). (**D**) Representative cardiac echography images and (**E**) quantification of LA in *FMKI*+NC and *FMKI*+AAV9 mice (n=6 per group, p=0.7161). (**F**) Representative HE images of whole heart in *FMKI*+NC and *FMKI*+AAV9 mice (n=3 per group). Scale bar: 2mm. (**G**) miR-224-5p expressions in ACFs (n=6 per group, p=0.0003) and ACFs-derived exosomes isolated from *FMKI*+NC and *FMKI*+AAV9 mice (n=6 per group, p=0.0008). (**H**) Quantification of AERP in *FMKI*+NC and *FMKI*+AAV9 mice (n=6 per group, p=0.0331). (**I**) qRT-PCR testing CACNA1C expression in the left atrium from *FMKI*+NC and *FMKI*+AAV9 mice (n=6 per group, p=0.0237). (**J**) Western blot testing Cav1.2 protein level in the left atrium from *FMKI*+NC and *FMKI*+AAV9 mice (n=6 per group, p=0.0002). *FMKI*+NC, *FMKI* mice treated with negative control virus; *FMKI*+AAV9, *FMKI* mice treated with AAV9-sh*miR-224-5p* virus for four weeks. The bar graph data are mean±SEM with individual values. p values were determined with two-tailed unpaired Student’s *t*-test in **C**, **E**, **G**, **H**, **I** and **J**.

**Supplementary Figure 11**


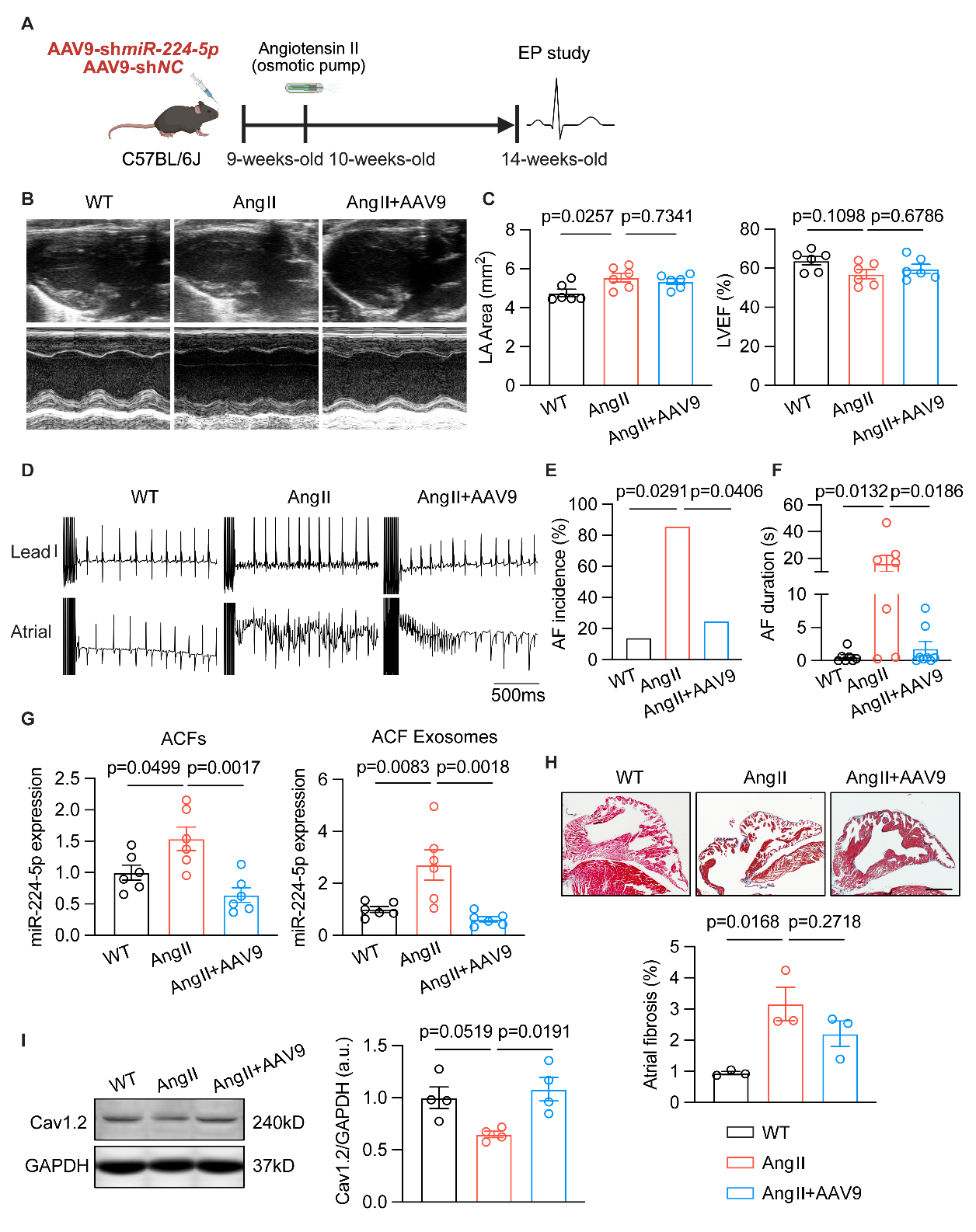


**Supplementary Figure 11**. **Knockdown CFs-miR-224-5p attenuated AF susceptibility in chronic Ang II-treated mice.**

(**A**) Study design for chronic Ang II-treated mice injected with NC or AAV9-sh*miR-224-5p*. (**B**) Representative cardiac echography images and (**C**) quantification of LA area (n=6 per group, WT vs Ang II p=0.0257, Ang II vs Ang II+AAV9 p=0.7341) and LVEF in WT, Ang II and Ang II+AAV9 mice (n=6 per group, WT vs Ang II p=0.1098, Ang II vs Ang II+AAV9 p=0.6786). (**D**) Representative simultaneous recordings of surface ECG (lead I) and intracardiac electrograms and (**E**) quantification of AF incidence (n=7 or 8 per group, WT vs Ang II p=0.0291, Ang II vs Ang II+AAV9 p=0.0406) and (**F**) duration in WT, Ang II and Ang II+AAV9 mice (n=7 or 8 per group, WT vs Ang II p=0.0132, Ang II vs Ang II+AAV9 p=0.0186). (**G**) miR-224-5p expressions in ACFs (n=6 per group, WT vs Ang II p=0.0499, Ang II vs Ang II+AAV9 p=0.0017) and ACFs-derived exosomes isolated from WT, Ang II and Ang II+AAV9 mice (n=6 per group, WT vs Ang II p=0.0083, Ang II vs Ang II+AAV9 p=0.0018). (**H**) Representative Masson images and quantification of atria fibrosis in WT, Ang II and Ang II+AAV9 mice (n=3 per group, WT vs Ang II p=0.0168, Ang II vs Ang II+AAV9 p=0.2718). Scale bar: 0.5mm. (**I**) Western blot testing Cav1.2 protein level in the left atrium of three groups of mice (n=4 per group, WT vs Ang II p=0.0519, Ang II vs Ang II+AAV9 p=0.0191). Ang II, Ang II treatment mice injected with negative control virus; Ang II+AAV9, Ang II treatment mice injected with AAV9-sh*miR-224-5p* virus. The bar graph data are mean±SEM with individual values. p values were determined with Fisher’s exact test in **E**, and Mann-Whitney test in **F**. p values were determined with two-tailed unpaired Student’s *t*-test in **C**, **G**, **H** and **I**.

**Supplementary Figure 12**


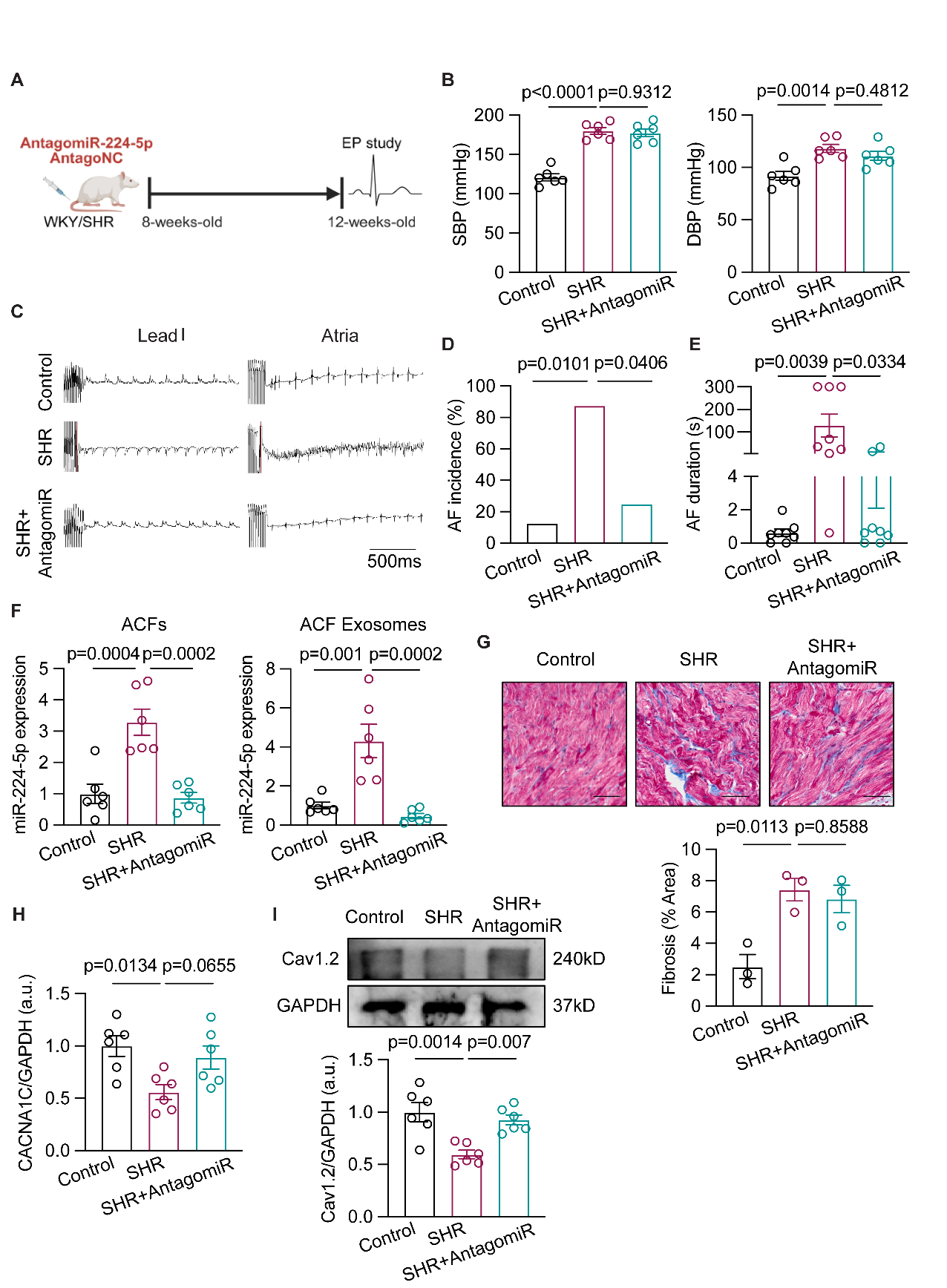


**Supplementary Figure 12**. **AntaogomiR-224-5p ameliorated AF susceptibility in chronic hypertension rats.**

(**A**) Study design for chronic hypertension rats (SHR) injected with NC or AntagomiR-224-5p. (**B**) Systolic blood pressure (n=6 per group, Control vs SHR p<0.0001, SHR vs SHR+AntagomiR p=0.9312) and diastolic blood pressure in Control, SHR and SHR+AntagomiR-224-5p rats (n=6 per group, Control vs SHR p=0.0014, SHR vs SHR+AntagomiR p=0.4812). (**C**) Representative simultaneous recordings of surface ECG (lead I) and intracardiac electrograms and (**D**) quantification of AF incidence (n=8 per group, Control vs SHR p=0.0101, SHR vs SHR+AntagomiR p=0.0406) and (**E**) duration in Control, SHR and SHR+AntagomiR-224-5p rats (n=8 per group Control vs SHR p=0.0039, SHR vs SHR+AntagomiR p=0.0334). (**F**) miR-224-5p expressions in ACFs (n=6 per group, Control vs SHR p=0.0004, SHR vs SHR+AntagomiR p=0.0002) and ACFs-derived exosomes isolated from Control, SHR and SHR+AntagomiR-224-5p rats (n=6 per group, Control vs SHR p=0.001, SHR vs SHR+AntagomiR p=0.0002). (**G**) Representative Masson images and quantification of atrial fibrosis in Control, SHR and SHR+AntagomiR-224-5p rats (n=3 per group, Control vs SHR p=0.0113, SHR vs SHR+AntagomiR p=0.8588). Scale bar: 50μm. (**H**) CACNA1C expression of atrial tissues in Control, SHR and SHR+AntagomiR-224-5p rats (n=6 per group, Control vs SHR p=0.0134, SHR vs SHR+AntagomiR p=0.0655). (**I**) Western blot testing Cav1.2 protein level in the left atria of rats (n=6 per group, Control vs SHR p=0.0014, SHR vs SHR+AntagomiR p=0.007). SHR, spontaneous hypertension rats injected with negative control antagomiR; SHR+AntagomiR-224-5p, spontaneous hypertension rats treated with antagomiR-224-5p. The bar graph data are mean±SEM with individual values. p values were determined with Fisher’s exact test in **D**, and Mann-Whitney test in **E**. p values were determined with two-tailed unpaired Student’s *t*-test in **B**, **F**, **G,** **H** and **I**.

**Supplementary Figure 13**

**
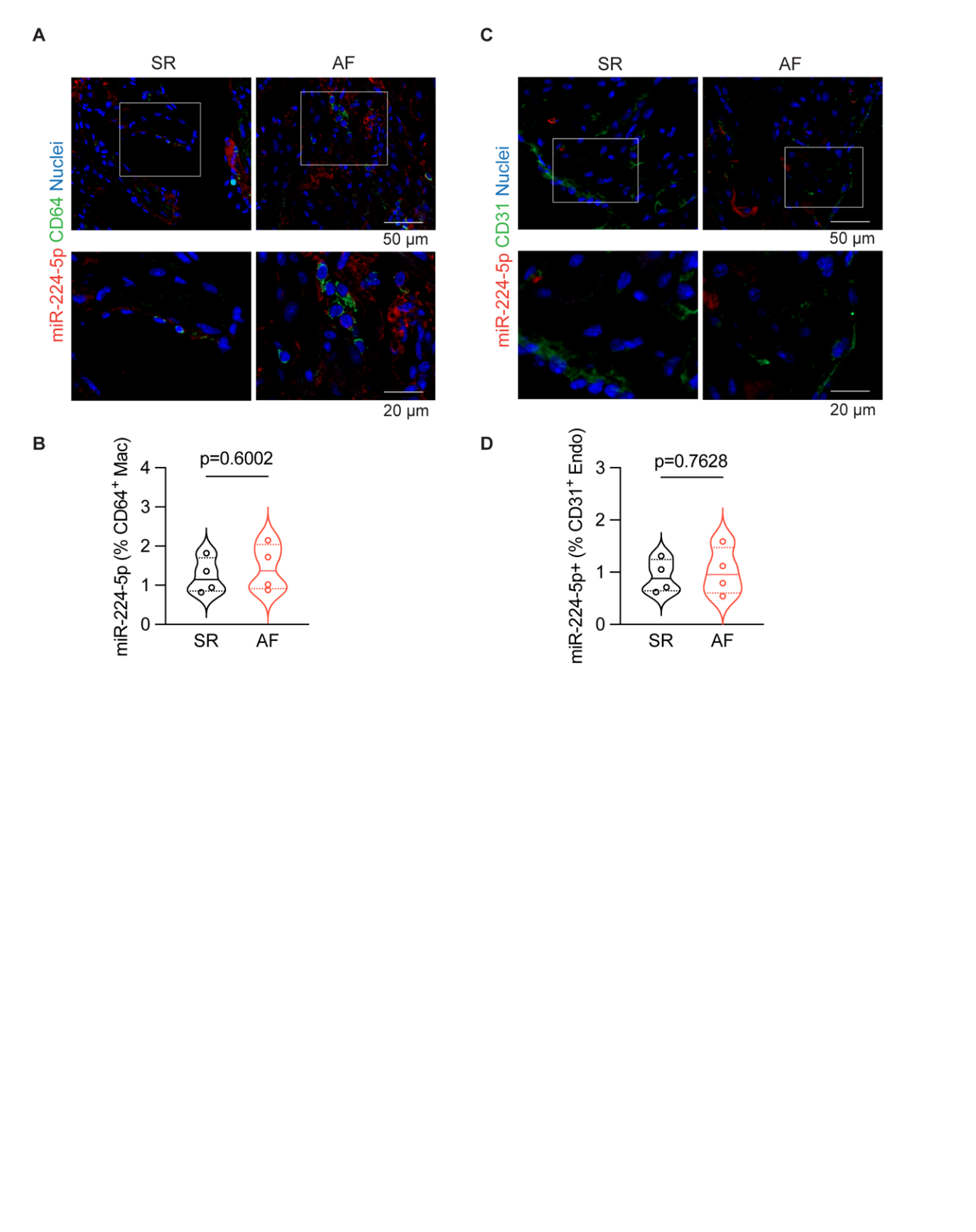
**

**Supplementary Figure 13**. **miR-224-5p expression of cardiac macrophages and endothelial cells in AF patient atria.**

(**A** and **B**) miR-224-5p positive and quantification of CD64^+^ macrophages in SR and AF patient atria (n=4 per group, p=0.6002), Scale bar: 50 or 20 μm. (**C** and **D**) miR-224-5p positive and quantification of CD31^+^ endothelial cells in SR and AF patient atria (n=4 per group, p=0.7628), Scale bar: 50 or 20 μm. The bar graph data are mean±SEM with individual values. p values were determined with two-tailed unpaired Student’s *t*-test in **B** and **D**.

**Supplementary Figure 14**


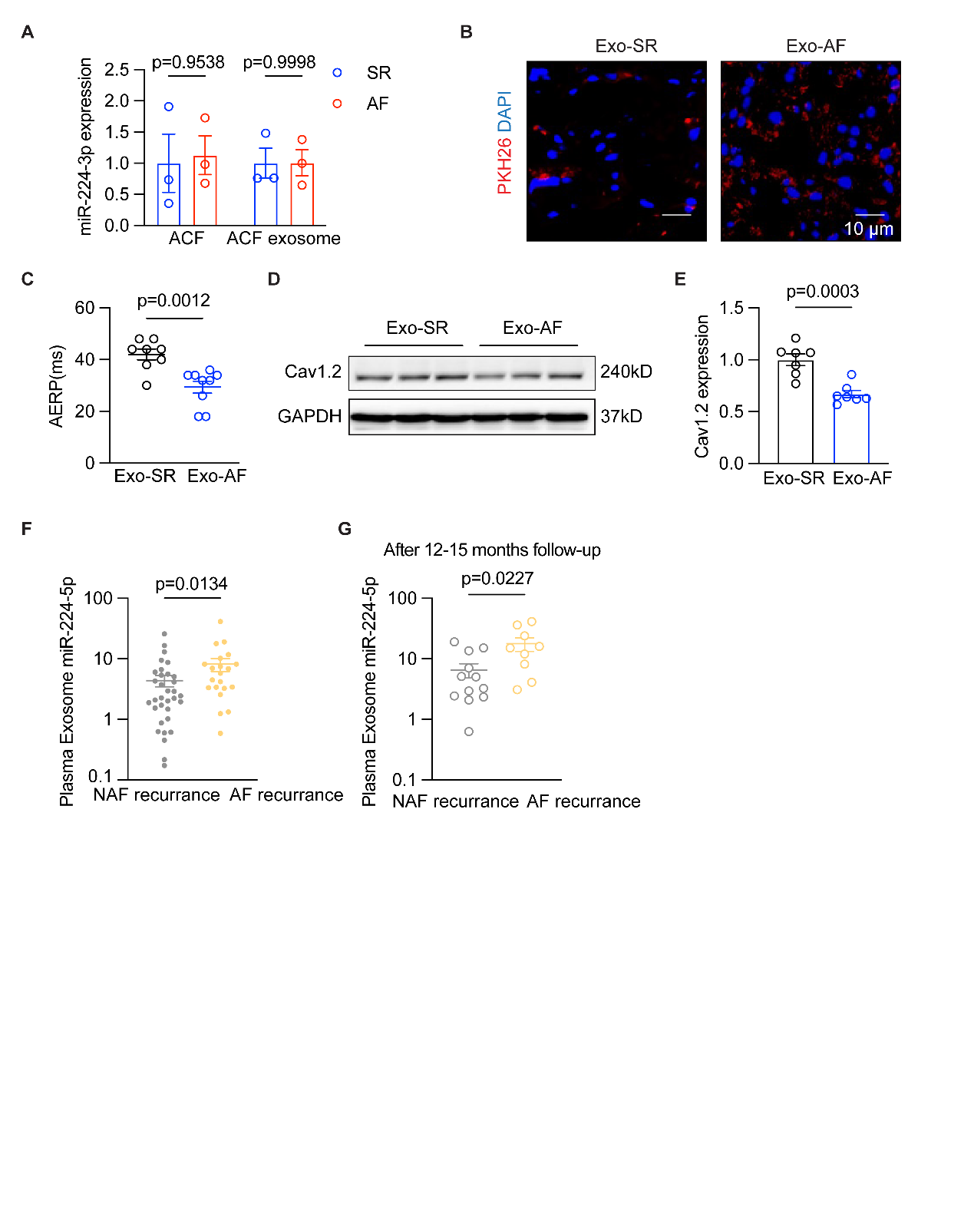


**Supplementary Figure 14**. **Plasma exosomes from AF patients resulted in shortened AERP in rats.**

(**A**) Quantification of miR-224-3p expressions in ACFs (n=3 per group, p=0.9538) and ACFs-derived exosomes from SR and AF patients (n=3 per group, p=0.9998). (**B**) PKH-26 label staining in atrial tissues after exosomes transfer into rats. Scale bar: 10μm (n=3 per group). (**C**) Quantification of AERP in Exo-SR and Exo-AF rat (n=8 or 9 per group, p=0.0012). (**D**) Representative Western blot images and (**E**) quantification of Cav1.2 protein level in rat atria (n=7 per group, p=0.0003). (**F**) Plasma levels of exosome miR-224-5p in NAF recurrence and AF recurrence patients at baseline (NAF recurrence n=33, AF recurrence n=21, p=0.0134) and after 12-15 months follow-up (NAF recurrence n=12, AF recurrence n=9, p=0.0227). Exo-SR, plasma exosomes of SR individuals; Exo-AF, plasma exosomes of AF patients. The bar graph data are mean±SEM with individual values. p values were determined with two-tailed unpaired Student’s *t*-test in **A**, **C**, **E** and **F**.

**Supplementary Table**

**Supplementary Table 1. Clinical Characteristics of 255 individuals (Figure 8H)**

| Characteristics | SR group (n=125) | AF group (n=130) | p value |
| --- | --- | --- | --- |
| Age | 62.66±9.49 | 65.9±11.20 | 0.0134^**^ |
| Gender, n (%) |  |  | 0.3708 |
| Male | 79 (63.2) | 74 (56.9) |  |
| Female | 46 (36.8) | 56 (43.1) |  |
| BMI (kg/m^2^) | 24.49±3.09 | 25.21±3.03 | 0.0631 |
| Smoking, n (%) | 68 (54.4) | 43 (33.1) | 0.0007^***^ |
| Hypertension, n (%) | 66 (52.8) | 64 (49.2) | 0.6169 |
| Diabetes, n (%) | 35 (28.0) | 29 (22.3) | 0.3147 |
| CHD, n (%) | 66 (52.8) | 58 (44.6) | 0.2112 |
| HF, n (%) | 24 (19.2) | 37 (28.5) | 0.1060 |
| CKD, n (%) | 13 (10.4) | 26 (20.0) | 0.0375^*^ |
| Aspirin | 64 (53.6) | 29 (22.3) | <0.0001^****^ |
| NOAC | 2 (1.54) | 93 (71.5) | <0.0001^****^ |
| Glucose (mmol/L) | 6.21±2.26 | 5.50±1.48 | 0.0033^**^ |
| TG (mmol/L) | 1.81±1.00 | 1.54±0.83 | 0.0192^*^ |
| CHOL (mmol/L) | 4.49±1.30 | 4.14±1.04 | 0.0176^*^ |
| Cr (mmol/L) | 73.55±48.55 | 80.08±38.74 | 0.2353 |
| UA (mmol/L) | 323.5±108.7 | 361.9±109.4 | 0.0054^**^ |
| NT-proBNP (pg/L) | 878.1±148.6 | 1216±160.6 | 0.1246 |
| LAD (mm) | 36.47±4.28 | 42.21±6.41 | <0.0001^****^ |
| RAD (mm) | 43.21±3.64 | 49.96±7.53 | <0.0001^****^ |
| LVEF (%) | 58.51±10.58 | 57.87±11.17 | 0.6377 |

BMI, body mass index; CHD, Coronary heart disease; CHOL, cholesterol; CKD; chronic kidney disease; Cr, creatinine; HF, Heart Failure; LAD, left atrial diameter; LVEF, left ventricle ejection fraction; NOAC, novel oral anticoagulant; NT-proBNP, N-terminal pro-B-type natriuretic peptide; RAD, right atrial diameter; TG, triglyceride; UA, uric acid. (*p<0.05, ** p<0.01, *** p<0.001, **** p<0.0001)

**Supplementary Table 2. Clinical Characteristics of 20 individuals provided left atrial tissues**

| Characteristics | SR group (n=10) | AF group (n=10) | p value |
| --- | --- | --- | --- |
| Age | 61.8±3.02 | 60.6±2.95 | 0.7795 |
| Gender, n (%) |  |  | >0.9999 |
| Male | 5 (50) | 5 (50) |  |
| Female | 5 (50) | 5 (50) |  |
| BMI (kg/m^2^) | 25.25±1.61 | 25.56±1.12 | 0.8795 |
| Smoking, n (%) | 6 (60) | 3 (30) | 0.3698 |
| Hypertension, n (%) | 4 (40) | 7 (70) | 0.3698 |
| Diabetes, n (%) | 2 (20) | 4 (40) | 0.6285 |
| CHD, n (%) | 6 (60) | 3 (30) | 0.3698 |
| HF, n (%) | 4 (40) | 5 (50) | >0.9999 |
| CKD, n (%) | 1 (10) | 1 (10) | >0.9999 |
| Aspirin | 4 (40) | 7 (70) | 0.3698 |
| NOAC | 1 (10) | 5 (50) | 0.1409 |
| Glucose (mmol/L) | 5.69±0.42 | 5.62±0.23 | 0.9002 |
| TG (mmol/L) | 1.65±0.22 | 1.41±0.22 | 0.45 |
| CHOL (mmol/L) | 5.05±0.66 | 4.34±0.18 | 0.3101 |
| Cr (mmol/L) | 112.0±41.81 | 76.3±5.34 | 0.4077 |
| UA (mmol/L) | 326.5±30.75 | 381.8±33.63 | 0.2406 |
| NT-proBNP (pg/L) | 280.4±63.07 | 420.4±121.0 | 0.3185 |
| LAD (mm) | 37.90±1.64 | 42.90±1.25 | 0.0259 |
| RAD (mm) | 44.70±1.63 | 49.70±2.23 | 0.0869 |
| LVEF (%) | 54.20±4.06 | 56.60±3.19 | 0.6478 |

BMI, body mass index; CHD, Coronary heart disease; CHOL, cholesterol; CKD; chronic kidney disease; Cr, creatinine; HF, Heart Failure; LAD, left atrial diameter; LVEF, left ventricle ejection fraction; NOAC, novel oral anticoagulant; NT-proBNP, N-terminal pro-B-type natriuretic peptide; RAD, right atrial diameter; TG, triglyceride; UA, uric acid. (*p<0.05, ** p<0.01, *** p<0.001, **** p<0.0001)

**Supplementary Table 3**

Baseline of Exo-Ctl and Exo-Ang II rats (**Figure 1E**)

|  | Exo-Ctl  (n=7) | Exo-Ang II  (n=7) |
| --- | --- | --- |
| Body Weight (g) | 258.1 ± 6.69 | 271.7 ± 3.77 |
| Heart Weight (mg) | 978.4 ± 9.30 | 1013 ± 9.73 |
| Left Atria Weight (mg) | 42.43 ± 0.95 | 43.86 ± 1.30 |
| Right Atria Weight (mg) | 41.14 ± 1.32 | 41.29 ± 0.87 |
| Ventricle Weight (mg) | 894.9 ± 9.24 | 927.4 ± 8.64 |

**Supplementary Table 4**

Basic ECG data of Exo-Ctl and Exo-Ang II rats (**Figure 1E**)

|  | Exo-Ctl  (n=7) | Exo- Ang II  (n=7) |
| --- | --- | --- |
| HR (bpm) | 443.7 ± 10.53 | 464.3 ± 6.95 |
| P wave (ms) | 23.43 ± 0.75 | 22.39 ± 0.55 |
| RR (ms) | 151.1 ± 5.36 | 151.8 ± 4.69 |
| PR (ms) | 46.63 ± 0.57 | 46.12 ± 0.69 |
| QRS (ms) | 17.00 ± 0.43 | 17.49 ± 0.26 |
| QT (ms) | 71.42 ± 0.43 | 71.49 ± 0.42 |

**Supplementary Table 5**

Baseline of Exo-Ctl, Exo-Ang II and Exo-siDicer+Ang II rats (**Figure 2A**)

|  | Exo-Ctl  (n=7) | Exo-Ang II  (n=8) | Exo- siDicer+Ang II  (n=8) |
| --- | --- | --- | --- |
| Body Weight (g) | 289.3 ± 6.67 | 274.3 ± 7.35 | 260.4 ± 8.16 |
| Heart Weight (mg) | 995.0 ± 8.20 | 994.6 ± 12.87 | 987.6 ± 11.11 |
| Left Atria Weight (mg) | 43.14 ± 1.01 | 40.25 ± 0.84 | 40.38 ± 1.54 |
| Right Atria Weight (mg) | 42.14 ± 1.32 | 42.00 ± 0.93 | 41.25 ± 1.31 |
| Ventricle Weight (mg) | 909.7 ± 7.05 | 912.4 ± 12.45 | 906.0 ± 9.82 |

**Supplementary Table 6**

Basic ECG data of Exo-Ctl, Exo-Ang II and Exo-siDicer+Ang II rats (**Figure 2A**)

|  | Exo-Ctl  (n=7) | Exo-Pacing  (n=8) | Exo- siDicer+Pacing  (n=8) |
| --- | --- | --- | --- |
| HR (bpm) | 447.1 ± 15.49 | 461.8 ± 12.77 | 450.0 ± 10.53 |
| P wave (ms) | 23.28 ± 0.54 | 22.67 ± 0.76 | 23.43 ± 0.43 |
| RR (ms) | 142.5 ± 6.60 | 140.1 ± 6.35 | 142.1 ± 5.02 |
| PR (ms) | 45.10 ± 0.64 | 42.01 ± 3.74 | 45.83 ± 0.53 |
| QRS (ms) | 17.97 ± 0.62 | 17.06 ± 0.43 | 16.52 ± 0.35 |
| QT (ms) | 71.62 ± 0.54 | 71.49 ± 0.57 | 71.05 ± 0.41 |

**Supplementary Table 7**

Baseline of Exo-NC and Exo-Mimic rats (**Figure 3D**)

|  | Exo-NC  (n=8) | Exo-Mimic  (n=8) |
| --- | --- | --- |
| Body Weight (g) | 282.4 ± 6.94 | 273.1 ± 9.18 |
| Heart Weight (mg) | 989.0 ± 12.31 | 991.8 ± 25.69 |
| Left Atria Weight (mg) | 42.75 ± 1.32 | 39.88 ± 1.84 |
| Right Atria Weight (mg) | 44.88 ± 1.93 | 41.75 ± 1.39 |
| Ventricle Weight (mg) | 901.4 ± 12.86 | 910.1 ± 23.25 |

**Supplementary Table 8**

Basic ECG data of Exo-NC and Exo-Mimic rats (**Figure 3D**)

|  | Exo-NC  (n=8) | Exo-Mimic  (n=8) |
| --- | --- | --- |
| HR (bpm) | 444.8 ± 15.84 | 452.0 ± 13.68 |
| P wave (ms) | 23.40 ± 0.68 | 22.91 ± 0.73 |
| RR (ms) | 143.1 ± 5.80 | 142.5 ± 6.66 |
| PR (ms) | 46.04 ± 0.71 | 45.35 ± 0.76 |
| QRS (ms) | 17.01 ± 0.65 | 16.98 ± 0.44 |
| QT (ms) | 72.23 ± 0.66 | 71.40 ± 0.58 |

**Supplementary Table 9**

Baseline of Exo-Ang II and Exo-Inhibitor+Ang II rats (**Figure 3J**)

|  | Exo- Ang II  (n=7) | Exo-Inhibitor+Ang II  (n=8) |
| --- | --- | --- |
| Body Weight (g) | 257.9 ± 8.43 | 263.8 ± 8.14 |
| Heart Weight (mg) | 982.4 ± 11.25 | 989.8 ± 7.00 |
| Left Atria Weight (mg) | 39.86 ± 1.72 | 40.13 ± 1.11 |
| Right Atria Weight (mg) | 41.86 ± 1.47 | 42.00 ± 0.68 |
| Ventricle Weight (mg) | 900.7 ± 9.6 | 907.6 ± 6.66 |

**Supplementary Table 10**

Basic ECG data of Exo-Ang II and Exo-Inhibitor+Ang II rats (**Figure 3J**)

|  | Exo-Ang II  (n=7) | Exo-Inhibitor+Ang II  (n=8) |
| --- | --- | --- |
| HR (bpm) | 457.9 ± 11.10 | 448.3 ± 13.13 |
| P wave (ms) | 23.19 ± 0.44 | 23.36 ± 0.59 |
| RR (ms) | 143.8 ± 5.45 | 141.3 ± 5.65 |
| PR (ms) | 46.20 ± 0.66 | 45.36 ± 0.80 |
| QRS (ms) | 16.54 ± 0.39 | 16.79 ± 0.36 |
| QT (ms) | 71.11 ± 0.44 | 70.67 ± 0.47 |

**Supplementary Table 11**

Baseline of NC and AgomiR-224-5p rats (**Figure 4A**)

|  | NC  (n=8) | AgomiR-224-5p  (n=8) |
| --- | --- | --- |
| Body Weight (g) | 271.8 ± 8.38 | 270.8 ± 7.50 |
| Heart Weight (mg) | 993.3 ± 14.28 | 999.8 ± 11.67 |
| Left Atria Weight (mg) | 42.50 ± 1.21 | 41.00 ± 0.63 |
| Right Atria Weight (mg) | 43.13 ± 1.48 | 43.00 ± 1.31 |
| Ventricle Weight (mg) | 907.6 ± 12.60 | 915.8 ± 10.48 |

**Supplementary Table 12**

Basic ECG data of NC and AgomiR-224-5p rats (**Figure 4A**)

|  | NC  (n=8) | AgomiR-224-5p  (n=8) |
| --- | --- | --- |
| HR (bpm) | 460.6 ± 9.75 | 455.9 ± 10.12 |
| P wave (ms) | 20.70 ± 0.50 | 20.51 ± 0.61 |
| RR (ms) | 152.0 ± 5.98 | 155.0 ± 4.01 |
| PR (ms) | 46.58 ± 0.46 | 46.14 ± 0.56 |
| QRS (ms) | 17.96 ± 0.33 | 17.31 ± 0.29 |
| QT (ms) | 71.13 ± 0.42 | 71.49 ± 0.32 |

**Supplementary Table 13**

Baseline of NC and AntagomiR-224-5p rats (**Figure 4H**)

|  | NC  (n=7) | AntagomiR-224-5p  (n=7) |
| --- | --- | --- |
| Body Weight (g) | 264.9 ± 11.33 | 269.9 ± 12.42 |
| Heart Weight (mg) | 993.7 ± 16.41 | 989.7 ± 17.45 |
| Left Atria Weight (mg) | 41.14 ± 1.03 | 41.71 ± 1.25 |
| Right Atria Weight (mg) | 41.86 ± 0.86 | 41.71 ± 1.34 |
| Ventricle Weight (mg) | 910.7 ± 16.00 | 906.3 ± 15.30 |

**Supplementary Table 14**

Basic ECG data of NC and AntagomiR-224-5p rats (**Figure 4H**)

|  | NC  (n=7) | AntagomiR-224-5p  (n=7) |
| --- | --- | --- |
| HR (bpm) | 459.0 ± 9.51 | 466.3 ± 10.51 |
| P wave (ms) | 20.63 ± 0.48 | 20.37 ± 0.58 |
| RR (ms) | 158.8 ± 5.82 | 159.0 ± 5.83 |
| PR (ms) | 45.98 ± 0.55 | 46.34 ± 0.51 |
| QRS (ms) | 17.47 ± 0.17 | 17.54 ± 0.20 |
| QT (ms) | 71.55 ± 0.34 | 71.04 ± 0.40 |

**Supplementary Table 15**

Baseline of WT and global *miR-224-5p^+/+^* mice (**Supplementary** **Figure 8A**)

|  | WT  (n=10) | *miR-224-5p^+/+^*  (n=8) |
| --- | --- | --- |
| Body Weight (g) | 23.68 ± 1.51 | 23.63 ± 2.57 |
| Heart Weight (mg) | 113.8 ± 6.55 | 109.0 ± 9.44 |
| Atria Weight (mg) | 6.8 ± 0.92 | 6.5 ± 0.53 |
| Ventricle Weight (mg) | 107.0 ± 6.00 | 102.5 ± 9.64 |
| Tibia Length (mm) | 16.24 ± 0.33 | 16.00 ± 0.27 |
| Atria Weight/Tibia Length (mg/mm) | 0.42 ± 0.05 | 0.41 ± 0.04 |
| LA Weight/Tibia Length (mg/mm) | 0.22 ± 0.03 | 0.21 ± 0.03 |
| RA Weight/Tibia Length (mg/mm) | 0.20 ± 0.03 | 0.20 ± 0.03 |
| Ventricle Weight/Tibia Length (mg/mm) | 6.59 ± 0.28 | 6.40 ± 0.55 |

**Supplementary Table 16**

Basic ECG data of WT and global *miR-224-5p^+/+^* mice (**Supplementary** **Figure 8A**)

|  | WT  (n=10) | *miR-224-5p^+/+^*  (n=8) |
| --- | --- | --- |
| HR (bpm) | 502.2 ± 36.33 | 507.1 ± 42.65 |
| P wave (ms) | 10.56 ± 0.90 | 10.07 ± 1.44 |
| RR (ms) | 117.90 ± 10.33 | 113.40 ± 10.96 |
| PR (ms) | 37.84 ± 2.48 | 36.31 ± 2.75 |
| QRS (ms) | 8.68 ± 1.58 | 8.69 ± 1.44 |
| QT (ms) | 20.51 ± 1.57 | 20.85 ± 1.23 |
| QTc (ms) | 20.54 ± 1.23 | 19.68 ± 1.31 |

**Supplementary Table 17**

Baseline of WT and *FMKI* mice (**Figure 6A**)

|  | WT  (n=8) | *FMKI*  (n=8) |
| --- | --- | --- |
| Body Weight (g) | 24.00 ± 1.71 | 23.21 ± 2.00 |
| Heart Weight (mg) | 118.6 ± 10.10 | 111.9 ± 9.28 |
| Atria Weight (mg) | 6.5 ± 0.53 | 6.4 ± 0.74 |
| Ventricle Weight (mg) | 112.1 ± 9.82 | 105.5 ± 8.67 |
| Tibia Length (mm) | 15.95 ± 0.31 | 16.01 ± 0.40 |
| Atria Weight/Tibia Length (mg/mm) | 0.41 ± 0.03 | 0.40 ± 0.04 |
| LA Weight/Tibia Length (mg/mm) | 0.21 ± 0.03 | 0.20 ± 0.02 |
| RA Weight/Tibia Length (mg/mm) | 0.20 ± 0.02 | 0.20 ± 0.02 |
| Ventricle Weight/Tibia Length (mg/mm) | 7.02 ± 0.51 | 6.58 ± 0.40 |

**Supplementary Table 18**

Basic ECG data of WT and *FMKI* mice (**Figure 6A**)

|  | WT  (n=8) | *FMKI*  (n=8) |
| --- | --- | --- |
| HR (bpm) | 500.3 ± 47.21 | 495.6 ± 31.19 |
| P wave (ms) | 10.44 ± 0.93 | 9.54 ± 1.42 |
| RR (ms) | 117.2 ± 10.17 | 117.0 ± 8.97 |
| PR (ms) | 39.31 ± 2.96 | 38.05 ± 2.52 |
| QRS (ms) | 8.00 ± 1.50 | 8.56 ± 1.33 |
| QT (ms) | 19.80 ± 1.9 | 20.09 ± 2.04 |
| QTc (ms) | 19.31 ± 1.72 | 19.93 ± 2.11 |

**Supplementary Table 19**

Baseline of *FMKI*+NC and *FMKI*+AAV9 mice (**Figure 6J**)

|  | *FMKI*+NC  (n=8) | *FMKI*+AAV9  (n=7) |
| --- | --- | --- |
| Body Weight (g) | 25.18 ± 0.73 | 25.07 ± 0.71 |
| Heart Weight (mg) | 114.9 ± 6.38 | 111.4 ± 7.00 |
| Atria Weight (mg) | 7.12 ± 0.39 | 6.43 ± 0.20 |
| Ventricle Weight (mg) | 107.8 ± 2.28 | 105.0 ± 2.67 |
| Tibia Length (mm) | 16.08 ± 0.28 | 16.04 ± 0.26 |
| Atria Weight/Tibia Length (mg/mm) | 0.44 ± 0.06 | 0.40 ± 0.04 |
| LA Weight/Tibia Length (mg/mm) | 0.22 ± 0.04 | 0.21 ± 0.03 |
| RA Weight/Tibia Length (mg/mm) | 0.23 ± 0.04 | 0.19 ± 0.02 |
| Ventricle Weight/Tibia Length (mg/mm) | 6.70 ± 0.35 | 6.55 ± 0.41 |

**Supplementary Table 20**

Basic ECG data of *FMKI*+NC and *FMKI*+AAV9 mice (**Figure 6J**)

|  | *FMKI*+NC  (n=8) | *FMKI*+AAV9  (n=7) |
| --- | --- | --- |
| HR (bpm) | 504.3 ± 30.84 | 512.4 ± 36.76 |
| P wave (ms) | 9.47 ± 1.04 | 10.37 ± 1.21 |
| RR (ms) | 113.2 ± 9.58 | 112.3 ± 7.07 |
| PR (ms) | 37.25 ± 2.84 | 37.11 ± 2.83 |
| QRS (ms) | 9.02 ± 1.16 | 8.51 ± 0.92 |
| QT (ms) | 19.98 ± 1.92 | 20.71 ± 1.39 |
| QTc (ms) | 20.34 ± 2.00 | 21.16 ± 1.44 |

**Supplementary Table 21**

Baseline of WT, Crem and Crem+AAV9 mice (**Figure 7A**)

|  | WT  (n=7) | Crem  (n=7) | Crem+AAV9  (n=8) |
| --- | --- | --- | --- |
| Body Weight (g) | 31.03 ± 1.86 | 32.49 ± 2.45 | 31.56 ± 1.66 |
| Heart Weight (mg) | 130.7 ± 7.27 | 141.9 ± 13.06 | 133.6 ± 10.51 |
| Atria Weight (mg) | 6.57 ± 0.53 | 13.29 ± 1.70 | 10.13 ± 1.25 |
| Ventricle Weight (mg) | 124.1 ± 6.96 | 128.6 ± 4.54 | 123.5 ± 3.54 |
| Tibia Length (mm) | 16.71 ± 0.16 | 16.73 ± 0.24 | 16.80 ± 0.20 |
| Atria Weight/Tibia Length (mg/mm) | 0.21 ± 0.03 | 0.34 ± 0.04 | 0.28 ± 0.07 |
| LA Weight/Tibia Length (mg/mm) | 0.22 ± 0.04 | 0.45 ± 0.09 | 0.32 ± 0.05 |
| RA Weight/Tibia Length (mg/mm) | 0.21 ± 0.03 | 0.34 ± 0.04 | 0.29 ± 0.07 |
| Ventricle Weight/Tibia Length (mg/mm) | 7.43 ± 0.39 | 7.68 ± 0.67 | 7.35 ± 0.55 |

**Supplementary Table 22**

Basic ECG data of WT, Ang II and Ang II+AAV9 mice (**Supplementary** **Figure 11A**)

|  | WT  (n=7) | Ang II  (n=7) | Ang II +AAV9  (n=8) |
| --- | --- | --- | --- |
| HR (bpm) | 495.7 ± 33.88 | 497.0 ± 37.20 | 504.5 ± 33.29 |
| P wave (ms) | 9.29 ± 1.42 | 10.42 ± 1.08 | 9.32 ± 1.14 |
| RR (ms) | 116.9 ± 6.33 | 114.9 ± 5.66 | 114.6 ± 7.08 |
| PR (ms) | 37.29 ± 1.97 | 37.74 ± 3.17 | 37.56 ± 2.55 |
| QRS (ms) | 8.99 ± 0.89 | 9.32 ± 1.01 | 9.48 ± 0.83 |
| QT (ms) | 19.78 ± 1.71 | 20.27 ± 0.79 | 19.84 ± 1.42 |
| QTc (ms) | 19.98 ± 1.84 | 20.54 ± 1.16 | 20.29 ± 1.46 |

**Supplementary Table 23**

Basic ECG data of SHR and SHR+AntagomiR-224-5p rats (**Supplementaary** **Figure 12**)

|  | Control  (n=8) | SHR  (n=8) | SHR+AntagomiR-224-5p (n=8) |
| --- | --- | --- | --- |
| HR (bpm) | 435.5 ± 10.64 | 434.0 ± 12.04 | 436.4 ± 11.85 |
| P wave (ms) | 21.23 ± 0.44 | 22.41 ± 0.48 | 21.91 ± 0.56 |
| RR (ms) | 151.5 ± 5.53 | 142.2 ± 5.06 | 135.4 ± 5.27 |
| PR (ms) | 44.78 ± 0.74 | 45.05 ± 0.54 | 45.12 ± 0.40 |
| QRS (ms) | 17.43 ± 0.56 | 16.50 ± 0.40 | 17.57 ± 0.15 |
| QT (ms) | 70.08 ± 0.54 | 70.16 ± 0.45 | 70.13 ± 0.32 |

**Supplementary Table 24**

Baseline of Exo-SR and Exo-AF rats (**Figure 8D**)

|  | Exo-SR  (n=8) | Exo-AF  (n=9) |
| --- | --- | --- |
| Body Weight (g) | 253.4 ± 9.26 | 263.9 ± 10.75 |
| Heart Weight (mg) | 974.9 ± 8.79 | 981.2 ± 8.83 |
| Left Atria Weight (mg) | 41.38 ± 0.92 | 42.89 ± 0.61 |
| Right Atria Weight (mg) | 41.63 ± 0.78 | 42.67 ± 1.05 |
| Ventricle Weight (mg) | 891.9 ± 8.22 | 895.7 ± 7.84 |

**Supplementary Table 25**

Basic ECG data of Exo-SR and Exo-AF rats (**Figure 8D**)

|  | Exo-SR  (n=8) | Exo-AF  (n=9) |
| --- | --- | --- |
| HR (bpm) | 452.9 ± 11.95 | 460.4 ± 8.52 |
| P wave (ms) | 21.18 ± 0.48 | 21.34 ± 0.40 |
| RR (ms) | 157.8 ± 5.77 | 155.8 ± 5.03 |
| PR (ms) | 45.81 ± 0.41 | 45.07 ± 0.38 |
| QRS (ms) | 17.65 ± 0.19 | 17.59 ± 0.20 |
| QT (ms) | 71.57 ± 0.32 | 71.16 ± 0.32 |

**Nonstandard Abbreviations and Acronyms**

| **ACF** | atrial cardiac fibroblast |
| --- | --- |
| **ACM** | atrial cardiomyocyte |
| **Ach** | acetylcholine |
| **AERP** | atrial effective refractory period |
| **AF** | atrial fibrillation |
| **Ang II** | angiotensin II |
| **APD** | action potential duration |
| **APD_50_** | action potential duration at 50% repolarization |
| **APD_90_** | action potential duration at 90% repolarization |
| **AAV** | adneo-associated virus |
| **CACNA1C** | Calcium voltage-gated channel subunit alpha 1c |
| **CVD** | Cardiovascular disease |
| **ECG** | electrocardiogram |
| **ECM** | extracellular matrix |
| **EV** | extracellular vesicle |
| **eWAT** | epididymal white adipose tissue |
| **Exo** | exosome |
| **GO** | gene ontology |
| **KEGG** | kyoto encyclopedia of genes and genome |
| **LA** | left atria |
| **LVEF** | left ventricle ejection fraction |
| **miRNA** | microRNA |
| **ncRNA** | noncoding RNA |
| **NTA** | Nanosight tracking analysis |
| **SHR** | spontaneous hypertension rat |
| **SR** | sinus rhythm |
| **TEM** | transmission electron microscope |
| **TnT** | Troponin T |
| **WGA** | Wheat Germ Agglutinin |
